# MAPK cascade gene family in *Camellia sinensis*: *In-silico* identification, expression profiles and regulatory network analysis

**DOI:** 10.1186/s12864-020-07030-x

**Published:** 2020-09-07

**Authors:** Archita Chatterjee, Abhirup Paul, G. Meher Unnati, Ruchika Rajput, Trisha Biswas, Tamalika Kar, Srijita Basak, Neelam Mishra, Ashutosh Pandey, Anurag Prakash Srivastava

**Affiliations:** 1 Department of Life Sciences, Garden City University, Bangalore, Karnataka India; 2 National Institute of Plant Genome Research, Aruna Asaf Ali Marg, New Delhi, India; 3 Department of Botany, St. Joseph’s College, Bangalore, Karnataka India

**Keywords:** *Camellia sinensis*, MAPK cascade, Expression profile, Functional interaction network, *In-silico*

## Abstract

**Background:**

Mitogen Activated Protein Kinase (MAPK) cascade is a fundamental pathway in organisms for signal transduction. Though it is well characterized in various plants, there is no systematic study of this cascade in tea.

**Result:**

In this study, 5 genes of Mitogen Activated Protein Kinase Kinase (MKK) and 16 genes of Mitogen Activated Protein Kinase (MPK) in *Camellia sinensis* were found through a genome-wide search taking *Arabidopsis thaliana* as the reference genome. Also, phylogenetic relationships along with structural analysis which includes gene structure, location as well as protein conserved motifs and domains, were systematically examined and further, predictions were validated by the results. The plant species taken for comparative study clearly displayed segmental duplication, which was a significant candidate for MAPK cascade expansion. Also, functional interaction was carried out in *C. sinensis* based on the orthologous genes in Arabidopsis. The expression profiles linked to various stress treatments revealed wide involvement of MAPK and MAPKK genes from Tea in response to various abiotic factors. In addition, the expression of these genes was analysed in various tissues.

**Conclusion:**

This study provides the targets for further comprehensive identification, functional study, and also contributed for a better understanding of the MAPK cascade regulatory network in *C. sinensis*.

## Background

Eukaryotic Mitogen Activated Protein Kinase (MAPK) cascades comprising of signalling enzymes, produce intracellular responses to environmental and developmental stimuli [1]. The increasing number of MAPK modules and their role in regulating complex cellular functions determine their significance in responsiveness of cells and organisms to their environment [1]**.** These signalling cascades have evolved into three kinases that are sequentially activated [1]. The three-kinase module has Mitogen Activated Protein Kinase Kinase Kinase (MAPKKK), Mitogen Activated Protein Kinase Kinase (MAPKK) and Mitogen Activated Protein Kinase (MAPK), which are categorized into subfamilies based on similar sequences, upstream modulation mechanism and sensitivity to the trigger-mechanisms by various Mitogen Activated Protein Kinase Kinases (also known as MEKs), which are dual specificity kinases [1, 2]. These kinases can be triggered only when both its tyrosine and threonine residues are phosphorylated [3]. MAPK activation is therefore a phosphorylation activity catalysed by MEK [1]. In this trigger-mechanism, the MAPKKKs activate MAPKK by phosphorylating serine and serine/threonine residues in the S/TxxxxxS/T motif, which in turn activates MAPK when threonine and tyrosine residues in the T-X-Y motif of T loop are phosphorylated [4, 5]. The MAPK signalling cascade is connected via a phospho-relay system to the upstream and downstream regulators [6]. These signalling cascades are clustered into complex subcellular networks that are interlinked [6, 7]. In the course of evolution, plants have come up with responsive mechanisms to perceive and transmit the stimuli in order to activate or repress a set of genes, thus coordinating the biotic and abiotic stresses in growth and developmental processes [4, 8]. Apart from the biotic and abiotic stresses, modulation of the MAPK-pathway trigger mechanism can also depend on the ROS signalling [9, 10]. These reactive oxygen species are formed when oxygen reduces partially and they can be produced intracellularly as well as apoplastically [10]. Studies suggest that multiple stresses such as salt stress, cold stress and drought stress stimulate ROS production, which in turn triggers MAPK signalling cascades [11]. Though the mechanism of activation and the downstream targets of the MAPK pathways are not recognized, ROS-induced activation of MAPKs seems to play a central role for mediating cellular responses to various stresses [11].

MAP kinases play an essential role in plant developmental processes as well as various signal transduction pathways [12]**.** Due to this significance of MAP kinases, many plants such as Arabidopsis [13]**,** rice [14–16] and maize [17] are functionally associated with a large number of MAPK genes. Meanwhile, a series of plant MAPK signalling cascades have also been studied. The first known plant MAPK signalling module, AtMEKK1-MKK4/5-MPK3/6 cascade has inborn flg22 signal transmission immunity [8, 18, 19]. In order to control the stomatal development and patterning, the complete MAPK signalling pathway of ANP3-MKK6-MPK4 and YDA-MKK4/5-MPK3/6 has been identified in Arabidopsis [8, 20]**.** It has been found that the MEKK1-MKK1/2-MPK4 module is essential to counter abiotic stress and has contributed to the freezing tolerance in Arabidopsis [8, 21, 22].

*C. sinensis* (Tea plant) is an important economic crop worldwide and is highly beneficial to humans [23, 24]. Though studies in terms of physiological stress, genomics and genetics have been carried out, MAPK genes have yet not been explored in the Tea plant genome. In this analysis, the MAPK and MAPKK family of genes were systematically defined on the basis of *in-silico* genome-wide search of tea with Arabidopsis as the reference genome. We studied the gene locations on scaffolds, their structures and their evolutionary aspect. Further, we analysed the interaction networks of proteins based on orthologous genes in Arabidopsis. The expression analysis patterns under various abiotic stresses and tissue expression profile was carried out by data mining from the publicly available TPIA database (http://tpia.teaplant.org/). This study might provide more prospects for functional analysis and also highlight the MAPK signalling cascade-mediated pathway of *C. sinensis* and beyond.

## Results

### Identification of MAPK genes in *Camellia sinensis*

In order to search for kinase domain in Tea Plant Information Archive (TPIA), the Hidden Markov Model (HMM) of the kinase domains (PF00069) (http://tpia.teaplant.org/) was utilized. The presence of kinase domains was then confirmed by using SMART and Pfam web tool. Arabidopsis has always been proven for its use as a model plant and thus the protein sequence of MKKs and MPKs obtained from it were used as reference sequences to search against the plant genomes. We used 10 MKK and 20 MPK protein sequences from *Arabidopsis thaliana* retrieved from TAIR (https://www.arabidopsis.org/index.jsp), as queries to search against the genome database of tea, Tea Plant Information Archive (http://tpia.teaplant.org/), by using the BLASTp algorithm (Additional file 1: Table S1 and S2). Further, a total of 5 MKK and 16 MPK genes were incorporated into the final dataset for the respective gene families. To perform a comparative genome wide study of the MAPK cascade genes of tea, MKK and MPK genes from Arabidopsis, rice, tomato, potato, capsicum and coffee were retrieved. Sequences from *Oryza sativa* were extracted from the Rice Genome Database (https://rapdb.dna.affrc.go.jp/) via BLAST algorithm using the MKK and MPK sequences from Arabidopsis. Similarly, MKK and MPK protein sequences of *Solanum lycopersicum*, *Solanum tuberosum*, *Capsicum annum* and *Coffea canephora* were extracted from the Solegenomics network (https://solgenomics.net/). Furthermore, the physicochemical properties of the extracted CsMKK and CsMPK protein sequences were evaluated using ExPASy (Tables 1 and 2). The length and molecular weights of the extracted MKK proteins ranged from 272 to 529 amino acids and 30.36574 to 58.67803 kDa, respectively (Table 1). While for MPKs, these values ranged from 330 to 975 amino acids and 41.67796 to 110.30164 kDa (Table 2). The theoretical pI values ranged from 5.44 to 7.66 for MKKs and 5.25 to 7.60 for MPKs indicating that most of the MKK and MPK proteins are acidic in nature with few proteins basic in nature (Tables 1 and 2). Four out five MKK proteins had more negative amino acid residues than positive ones (Table 1).

**Table 1:** Sequence characteristics and physicochemical properties of MKK genes in *C. sinensis*

Gene ID	***Arabidopsis thaliana*** ID	Locus position	Gene length (bp)	Protein length (aa)	Mol. Wt. (KD)	PI value	No. of negative residues	No. of positive residues	Hydrophobicity	Instability index
**TEA024893.1**	AT4G26070	Scaffold160: 1626938:1616882: -	10,056	354	39.33813	6.25	35	32	−0.15	45.84
**TEA012409.1**	AT5G40440	Scaffold2212: 611911:605139: -	6772	529	58.67803	5.44	63	48	−0.12	42.50
**TEA007510.1**	AT3G21220	Scaffold3886: 198586:199554: +	968	322	35.83497	7.66	32	33	−0.32	52.52
**TEA015514.1**	AT5G56580	Scaffold3508: 487640:492858 s:+	5217	272	30.36574	5.50	33	25	− 0.12	42.46
**TEA008204.1**	AT1G73500	Scaffold712: 770422:769445: -	977	325	36.09620	7.66	32	33	−0.32	53.90

**Table 2:** Sequence characteristics and physicochemical properties of MPK genes in *C. sinensis*

Gene ID	***Arabidopsis thaliana*** ID	Locus position	Gene length (bp)	Protein length (aa)	Mol. Wt. (KD)	PI value	No. of negative residues	No. of positive residues	Hydrophobicity	Instability index
**TEA016315.1**	AT1G10210	Scaffold3231: 722182:725302: -	3120	384	44.22532	6.70	47	45	−0.25	40.56
**TEA031435.1**	AT1G59580	Scaffold3918: 1637634:1641477: +	3843	421	47.94427	5.97	52	45	−0.22	38.69
**TEA032724.1**	AT2G18170	Scaffold1618: 847162:843216: -	3946	368	42.22203	7.60	41	42	−0.16	42.71
**TEA026040.1**	AT3G45640	Scaffold282: 872101:866535: -	5566	372	42.75504	5.25	50	33	−0.19	45.48
**TEA020852.1**	AT3G45640	Scaffold1757: 1196700:1204130: +	7430	365	41.67796	5.89	48	38	−0.19	39.34
**TEA006436.1**	AT4G01370	Scaffold2042: 894464:906856: +	12,392	367	42.24318	6.28	46	42	−0.35	41.82
**TEA021759.1**	AT4G01370	Scaffold214: 887906:895285: +	7379	330	38.20672	5.41	46	35	−0.28	46.69
**TEA024415.1**	AT2G43790	Scaffold706: 1260385:1247078: -	13,307	507	57.23870	6.06	55	46	−0.23	47.75
**TEA012905.1**	AT3G18040	Scaffold1154: 723171:731463: +	8292	587	66.86676	6.99	81	80	−0.56	40.45
**TEA015676.1**	AT3G18040	Scaffold3483: 338662:345558: +	6896	569	64.24032	6.43	73	68	−0.33	40.59
**TEA026883.1**	AT5G19010	Scaffold3679: 822261:838355: +	16,094	975	110.30164	9.22	120	144	−0.39	43.17
**TEA018880.1**	AT3G14720	Scaffold327: 693505:699204: +	5699	599	67.27707	9.22	65	80	−0.36	41.47
**TEA031053.1**	AT5G19010	Scaffold1571: 1385086:1378569: -	6517	587	66.61556	9.09	68	83	−0.37	41.46
**TEA022253.1**	AT3G14720	Scaffold1213: 332831:319166: -	13,665	792	90.12101	9.25	98	119	−0.41	38.29
**TEA022268.1**	AT3G18040	Scaffold568: 667443:685736: +	18,293	517	59.33552	6.75	66	64	−0.20	39.91
**TEA007103.1**	AT1G07880	Scaffold3534: 1335999:1362220: -	26,222	798	90.20825	6.18	92	82	−0.13	42.00

Similarly, for the MPK genes, 11 out of 16 proteins showed more number of negative amino acid residues than positive ones (Table 2). The grand average of hydropathy (GRAVY) in all extracted MKK and MPK proteins were negative values ranging from − 0.12 to − 0.32 and − 0.13 to − 0.39 respectively, indicating that all 21 proteins are hydrophilic in nature (Tables 1 and 2). The instability index of 17 proteins were above 40 and only 4 MPKs (TEA031435.1, TEA020852.1, TEA022253.1, TEA022268.1), had the instability index below the given level (Tables 1 and 2). This indicates that most of the screened proteins are unstable [25]. TMHMM server was used to check for the presence of transmembrane helices and it was concluded that there were none (Additional file 2: Fig. S1 and S2). The subcellular locations of all the identified 21 proteins were predicted using BaCelLo (http://gpcr.biocomp.unibo.it/bacello/) (Additional file 1: Table S3 and S4).

The locus position, gene length, protein length, molecular weight, pI value, number of negative and positive residues, hydrophobicity and instability index were analysed by using ExPASy web server.

The locus position, gene length, protein length, molecular weight, pI value, no. of negative and positive residues, hydrophobicity and instability index were analysed by using ExPASy web server.

### Phylogenetic analysis of MKKs and MPKs

**Fig. 1:**
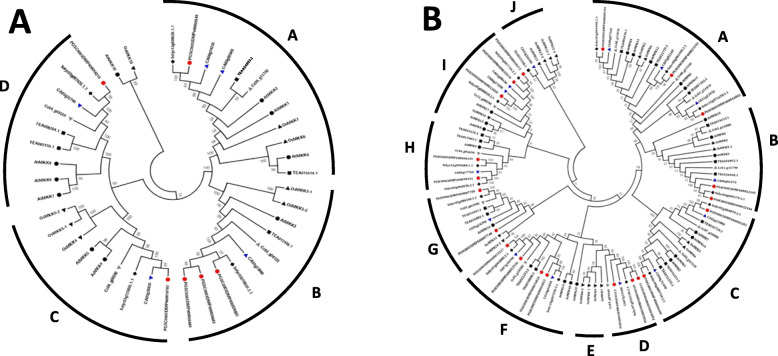
Phylogenetic analysis of MKK (**a**) and MPK (**b**) proteins. The analysis was carried out among *C. sinensis* (black square), *A. thaliana* (black circle), *O. sativa* (black triangle), *S. lycopersicum* (black rhombus), *S. tuberosum* (red circle), *C. annum* (blue triangle), and *C. canephora* (grey triangle). The full-length MKK and MPK protein sequences were aligned using MUSCLE tool, and the phylogenetic tree was constructed using MEGA 7.0.14 by the neighbor-joining (NJ) method with default parameters and 1000 bootstrap replicates

A phylogenetic analysis of MKK and MPK protein sequences from *C. sinensis, A. thaliana, O. sativa, S. lycopersicum, S. tuberosum, C. annum* and *C. canephora* was carried out to formulate the evolutionary relationship among them. We amassed 10 MKK protein sequences from *A. thaliana,* 8 sequences from *O. sativa,* 4 sequences from *S. lycopersicum*, 6 sequences from *S. tuberosum*, 5 sequences from *C. annum* and 4 sequences from *C. canephora.* Further, we performed BLASTp searches across the tea plant genome database using the Arabidopsis sequences which revealed 5 MKK proteins. Similarly, the evolutionary relationship was analysed among 20 MPK protein sequences from *A. thaliana*, 17 sequences from *O. sativa,* 14 sequences from *S. lycopersicum*, 21 sequences from *S. tuberosum*, 14 sequences from *C. annum* and 12 sequences from *C. canephora*. Further, the BLASTp searches were performed using Arabidopsis MPK sequences in TPIA database, which revealed 16 MPK proteins. Therefore, the phylogenetic trees were constructed using a total of 42 MKK and 117 MPK protein sequences by the neighbor-joining (NJ) method (Fig. 1a and b), minimum evolution (ME) method (Additional file 2: Fig. S3) and maximum likelihood (ML) method (Additional file 2: Fig. S4).

As per phylogenetic analysis, NJ tree for MKK got split into 4 distinct clades with AtMKK10 and OsMKK10 forming a separate lineage. Genes were uniformly distributed in all these 4 clades named as A, B, C and D. However, the clade B has a greater clustering of *S. tuberosum* (Potato) sequences. On the other hand, the NJ tree for MPK was stringently divided into 10 clades, namely A, B, C, D, E, F, G, H, I and J with clades D, F and I having a greater clustering of the potato MPK sequences and clade F consisting of only OsMPK genes namely, OsMPK7, OsMPK8–1, OsMPK8–3 and OsMPK9. Further, clade J showed a greater clustering of OsMPK genes. The homologs for both MKK and MPK genes could be identified from phylogenetic analysis. Also, the orthologous pair was obtained depending on phylogenetic relationships: AtMKK3 (AT5G40440.1) and TEA012409.1. The MPK tree however did not show any significant orthologous gene.

### Domain analysis of tea MKKs and MPKs

**Fig. 2:**
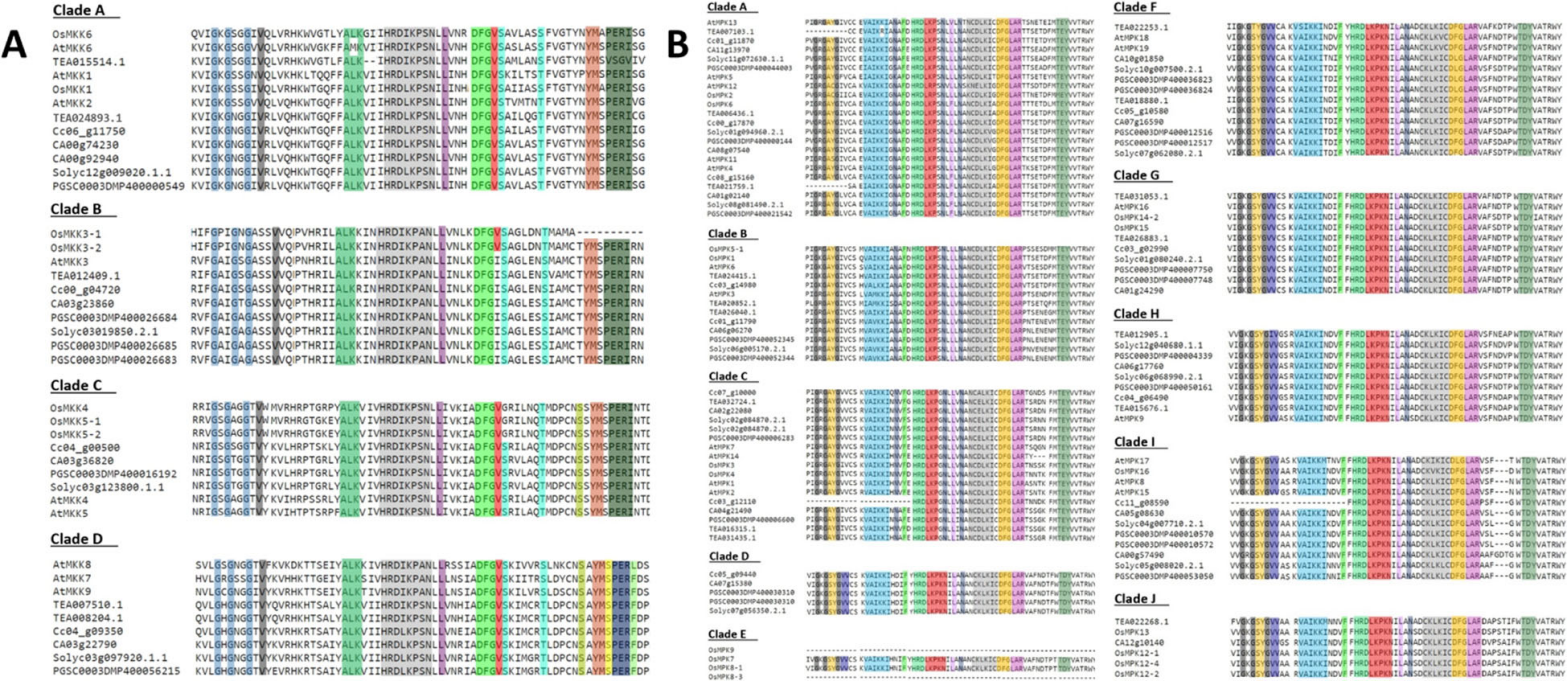
Alignment of important domains in (**a**) MKK and (**b**) MPK protein sequences. The amino acid sequences from *A. thaliana, C. sinensis, O. sativa, S. lycopersicum, S. tubersosum, C. annum and C. canephora* were aligned using MUSCLE tool (https://www.ebi.ac.uk/Tools/msa/muscle/) with default settings. The conserved domains are highlighted in different colours

All the MKKs and MPKs obtained were subjected to sequence alignment for the presence of conserved domains. The identified signature sequences are: (i) the phosphate binding P-loop, GxGxxG present in MPK and MKK genes, which is further classified as IGxGxYGxV for MPKs [26], showing variations in 1 MPK gene of clade C, and 2 of the MPK genes in clade A, clade E and clade I, found within the ATP binding signature sequence for PKs (ii) the ‘template kinase’ signature found within the PK domain, (iii) the catalytic C-loop, D(L/I/V) K found within the active site signature of S/T PK, and (iv) the activation-or T-loop, TxY. These domains are highlighted in the alignment figures for both MKK and MPK, shown in Fig. 2a and b.

The additional conserved domains are highlighted for MPKs and MKKs in Additional Files 3 and 4 respectively. Besides these sequences, in the C-terminal extensions, the two conserved domains found outside the PK domain were: (a) the common docking (CD) domain with a consensus sequence LH(D/E)xx(D/E)(D/E) PxC, found in the MPK genes of clade A and B (Additional file 3: Fig. S7), and (b) the EF-hand calcium binding protein in the MKK genes of clade B (Additional file 4: Fig. S8).

In the MPKs, the “VA” preceded the “IKKIxxxF” motif wherein “VAIKKIxxxF” motif was highly conserved in all the *C. sinensis* genes taken under research, with a few exceptions (Fig. 2a and b; Additional file 3: Fig. S7). In one case, TEA022253.1 of clade F has the sequence “VSIKKIxxxF” and TEA022268.1 of clade J, encodes the sequence “VAIKKMxxxF”. TEA021759.1 and TEA020852.1 in clades A and B, respectively have their initial amino acid residue substituted by ‘I’ for a ‘V’ (Additional file 3: Fig. S7). Considering the highly conserved nature of the “VAIKKIxxxF” motif, such sequence variations as observed in the above exceptions are probably a result of sequencing error or due to some cultivar definitive single nucleotide polymorphism [27]. This is a part of the ATP binding region, and there are no significant reports that this small variation can impair ATPase enzyme activity [27]. The motif “TEY” is only present among the MPK genes of clades A, B and C, with it being modified to “TDY” in all the MPK genes of other clades (Fig. 2b). The phosphate binding P-loop too has a significant change of two additional ‘V’ residues in all the MPK genes from clades D to J which is not the case in clades A, B and C (Fig. 2b). The catalytic C-loop domain was also highly conserved in all the plant species under study. Remarkably, instead of the S/T kinase signature that is common to the MPK subfamily, the MPK and MKK sequences of *C. sinensis* encode the canonically active signature of the Tyr kinases. A few MKK genes of clade A and D had the DLK active site within this signature (Additional file 4: Fig. S8). Only in 2 MPK genes of clade A, the DLK active site was replaced with the DLR motif within this signature (Fig. 2b; Additional file 3: Fig. S7). The FxDIYxxxELM motif was seen to be FxDVYxxxELM in MPK genes of clade A, B and AtMPK15 of clade I (Additional file 3: Fig. S7). Other specific MPK characteristic domains were also observed and were found to be conserved across all datasets.

The ATP binding region in the MPK genes of *C. sinensis* always terminated with the sequence “VAIKK”, while the same gets modified as “(F/Y/L)ALK” at the C-terminal end of tea MKK ATP binding region (Fig. 2a and b). The MAPK C-loop is referred as “D(L/I/V)K” and the sequence “HRDLKPxN” is highly conserved for tea MPKs, whereas for the MKKs, it gets modified to “HRD(L/I/V)KPxN” (Fig. 2a and b; Additional file 3: Fig.S7; Additional file 4: Fig.S8). DFGV(S/T)xxxxx(S/T)xxxxx(S/T) is a profoundly conserved sequence, and is present in all the members of clade D and clade C, excluding the OsMKK genes of clade C (Fig. 2a and b). These OsMKK genes do not contain the first S/T, which is considered to be a part of the activation loop (Fig. 2 a and b).

### Motif composition and gene structure analysis of the MKK and MPK genes

**Fig. 3:**
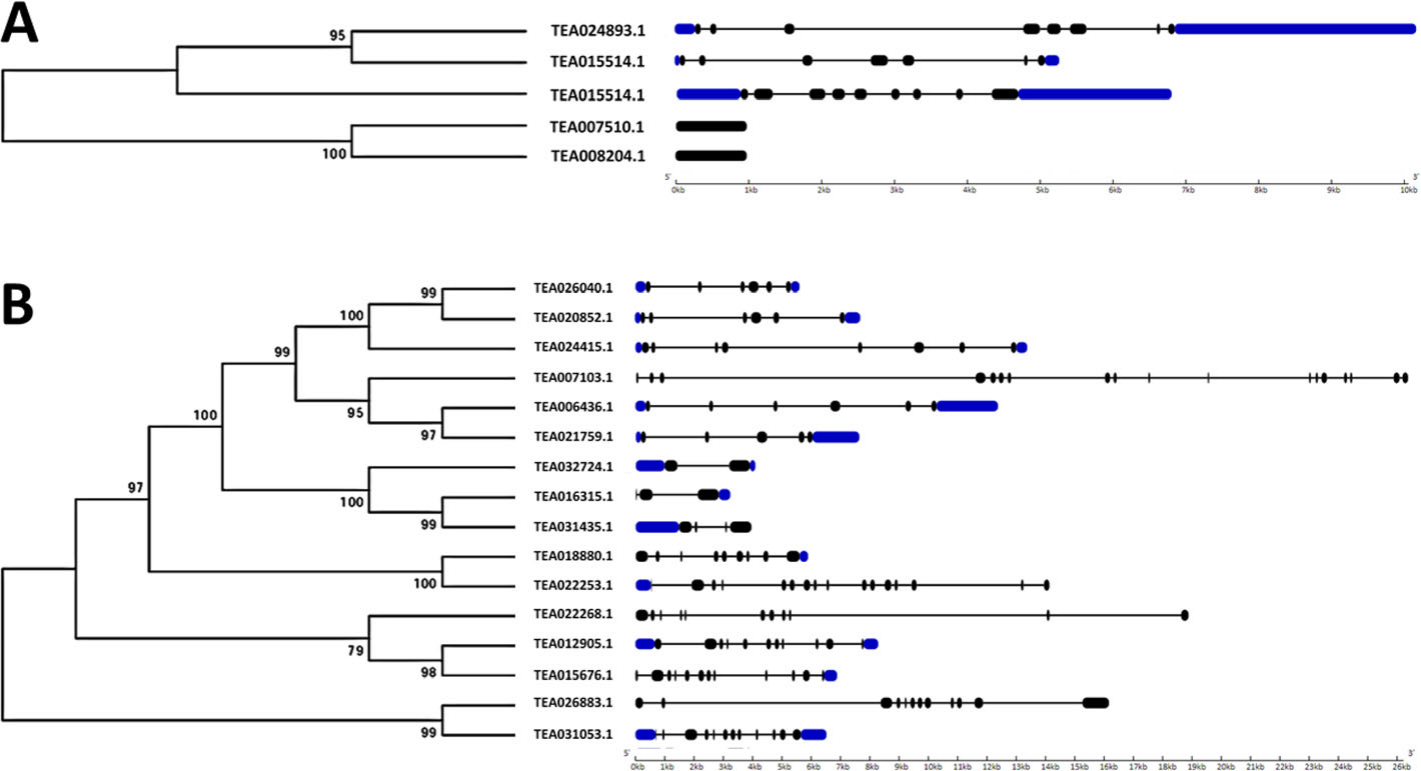
The exon/intron structure of (**a)** MKK and **(b)** MPK genes in *C. sinensis****.*** Gene structure maps were drawn with the Gene Structure Display Server 2.0. Black boxes represent exons, blue boxes represent the UTRs and black lines represent introns. The gene length can be estimated by using the scale (in kb) at the bottom

**Fig. 4:**
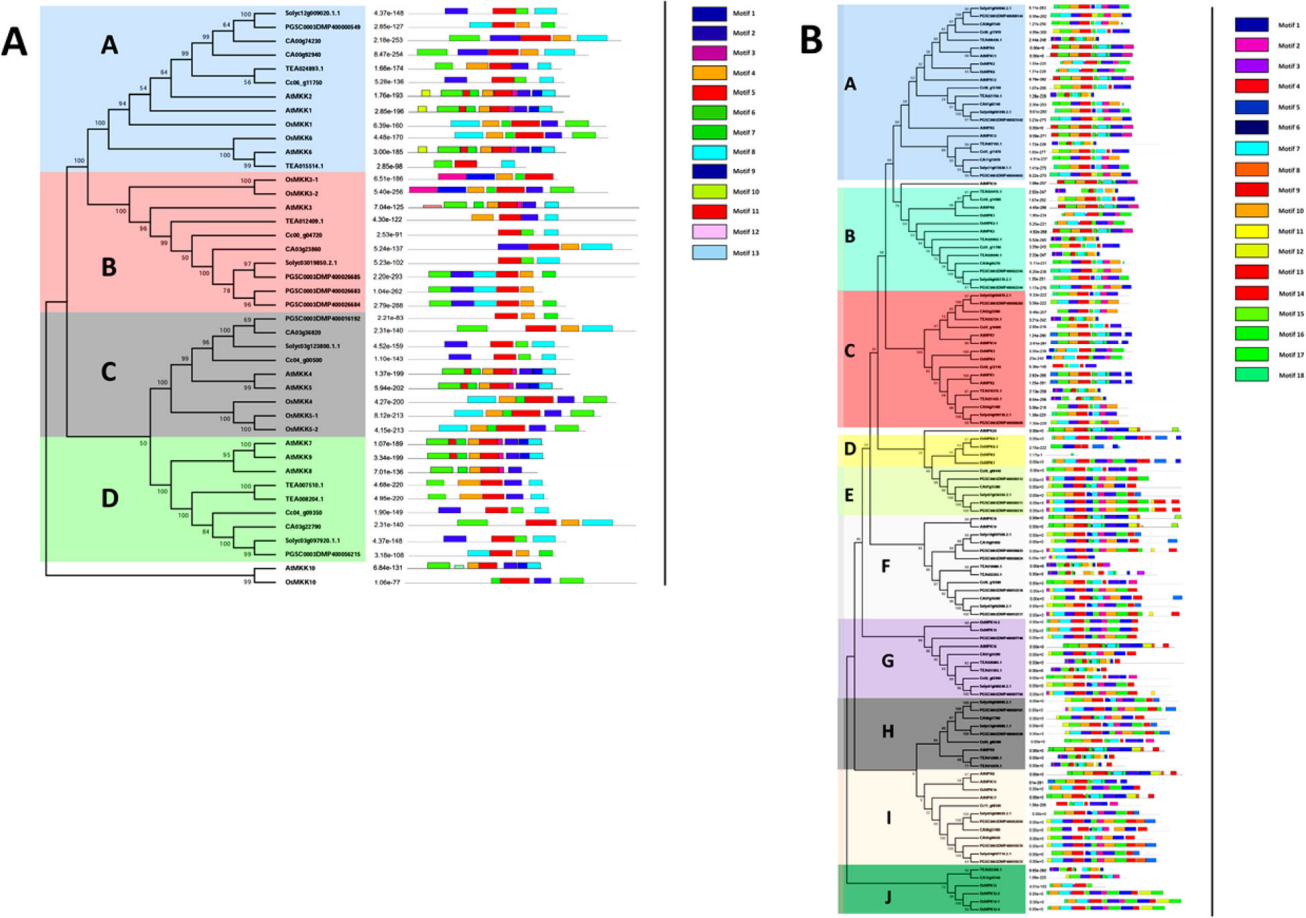
Schematic representation of motif signatures in **(a)** MKK and **(b)** MPK protein sequences**.** The motif analysis was carried out among *C. sinensis, A. thaliana, O. sativa, S. lycopersicum, S. tuberosum, C. annum* and *C. canephora*, by using MEME suite tool. 13 motifs in MKK and 18 motifs in MPK were identified

To analyse the sequential characteristics of MKK and MPK genes from tea plant, a correlative study of the conserved motifs from the protein sequences in *A. thaliana, C. sinensis, O. sativa, S. lycopersicum, S. tubersosum, C. annum* and *C. canephora*, was carried out by using MEME suite tool (Fig. 4a and b). Furthermore, we also obtained the motif logos (Additional file 2: Fig. S5 and S6). In total, 13 MKK and 18 MPK motifs were determined. Also, the visualization and analysis of intron and exon structures of MKK and MPK genes was carried out in the above-mentioned plants, by using Gene Structure Display Server 2.0 (Fig. 3a and b). MKK and MPK genes in the same clade have almost identical motifs and exon/intron architecture, and thus may have similar functions. In all the four clades of MKK genes, motif 5 is conserved (Fig. 4a). In clade A and C of this gene family, apart from motif 5, motif 8 was also fully conserved. Most of the MKK genes in all the clades had motif 8 conserved at their C terminal. Similarly, for MPK gene family, other than clade D and Cc03_g12110 of clade C, motif 15 was present in all the MPK genes (Fig. 4b). Also, except clade D, motif 7 and 13 were found to be conserved in all the other clades. Here, in clade D, only motif 18 was conserved and in clade E, motif 2 and 9 were conserved. Further, clades A, B, E, G and H, showed conservation of motif 1 and 10. Apart from these clades, motif 1 was also found to be conserved in clades C and I. Subsequently, in clades E, F, G, H and I, motif 6 and 17 were conserved. Moreover, presence of variable motifs was observed at the C-terminal end of the MPK genes that were distributed in 10 clades (Fig. 4b).

Study of gene structure indicated variations in both number and length of introns as well as exons, resulting in gene length variation. Non-coding sequences like introns, abundant within a genome, are regarded as an indicator of genome complexity [27, 28] and analysis of the intron patterns will provide insights into gene structure and evolution [27, 29–31]. The analysis of intron/exon architecture in the *C. sinensis* genes revealed that 3 out of 5 MKK genes (60% of tea MKK genes) had 6–8 introns, and the remaining 2 genes which were present in clade D of the MKK gene family, had no introns (Fig. 3a and b). Also, TEA12409.1 is found to be the longest MKK gene in *C. sinensis,* with 8 introns. The MKK genes in the same clade had similar number of introns and exons which supports their close phylogenetic relationship. Furthermore, the MPK gene family had 44% of its genes with 10 or more introns (Fig. 3b) which were members of either clade A, F, G or H. Generally, the MPK genes belonging to the same clade had similar number of exons and introns. Only TEA007103.1 showed variation with respect to number of introns and exons, in comparison to the other genes in its clade. Variation in the size of exons and introns was observed in both MKK and MPK genes. For MKK genes, TEA024893.1 with 10,056 bp was the longest gene. In addition, this gene contained the longest intron sequence of 3107 bp and had the longest UTR sequence of 3273 bp. The longest exon was found in TEA008204.1, with a length of 977 bp. TEA007510.1 was found to be the shortest gene among the 5 MKKs, comprising of 968 bp whereas the shortest UTR with 66 bp was observed in TEA015514.1. TEA012409.1 featured the smallest intron sequence, comprising of 82 bp while, TEA024893.1 and TEA015514.1 featured the smallest exons, having a sequence length of 46 bp. Similarly for MPKs, the longest gene was found to be TEA007103.1 with 26,222 bp and this gene simultaneously featured the longest intron with 10,579 bp. TEA026883.1 contained the longest exon sequence of 898 bp whereas TEA006436.1 had the longest UTR sequence of 2097 bp. TEA016315.1 was the shortest gene, with 3120 bp. The shortest intron was observed in TEA022268.1, TEA026883.1 and TEA031053.1, having a sequence length of 83 bp. TEA031053.1 contained the shortest exon, with 5 bp while TEA021759.1 had the shortest UTR sequence of 155 bp.

### The genomic distribution map and evolutionary pressure of MKK and MPK genes

**Fig. 5:**
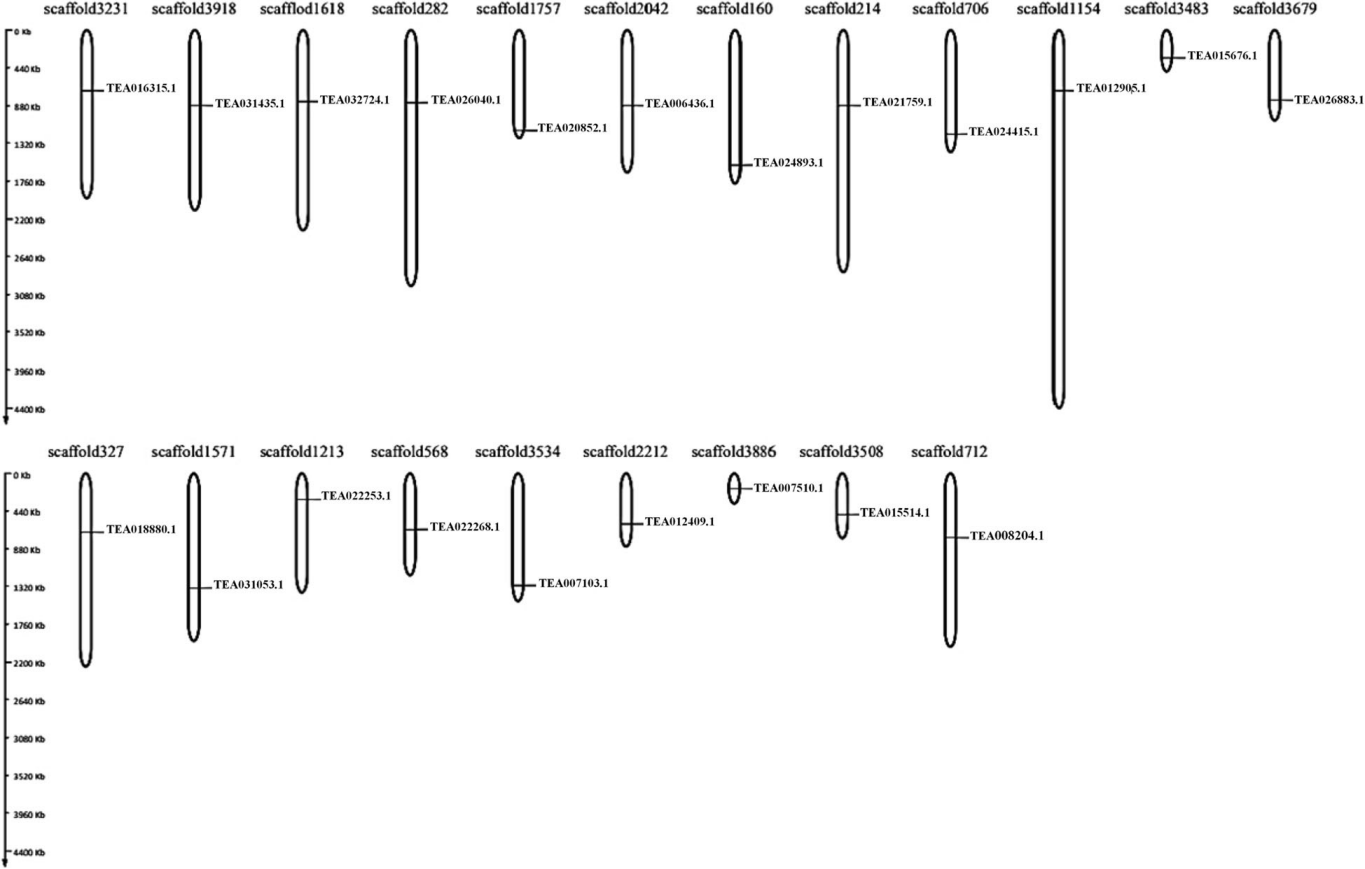
The scaffold distribution of the 21 MAPK genes in *C. sinensis*. MapGene2chromosome web v2 (MG2C) software tool was used to map genes onto the scaffolds. The scaffolds are drawn to scale and the scaffold numbers are indicated on the top

The structure of 5 MKK and 16 MPK genes in tea plant were further analysed to understand their induction under abiotic stresses and tissue expression. Due to the lack of chromosome-level assembly data for tea, their MKK and MPK gene distribution was mapped onto the scaffold instead of chromosome. Therefore, the analysis showed that the 5 MKK and 16 MPK genes were spread across 21 genomic scaffolds (Fig. 5). Across these scaffolds, individual genes were positioned such that one gene was located on a single scaffold. Thus, genes belonging to same clades were found to be located on different scaffolds. Additionally inside these 5 MKK and 16 MPK genes, we found that, 4 of the dS/dN values were < 1 in pairwise comparisons, suggesting that these gene pairs were under positive selection, which include TEA007966.1/TEA008204.1 (dS/dN of 0.8333), TEA012409.1/TEA015514.1 (dS/dN of 0.9571), TEA012409.1/TEA024893.1(dS/dN of 0.8973), TEA015514.1/TEA02893.1(dS/dN of 0.8082). The remaining 6 comparisons did not show any selection (Additional file 1: Table S5). Similarly, for MPK genes, pairwise comparative genes showed that a total of 82 values were under positive selection, 33 paired genes were under negative selection, and 7 paired genes were Nan (Additional file 1: Table S6). The outcomes of the gene distribution and the dN/dS ratios indicated the following: the 5 MKK and 16 MPK genes have been extensively distributed across the tea plant genome. There was a lack of tandem duplication in the 5 MKK and 16 MPK genes due to their close distribution (a tandem duplication event is a chromosomal region of about 200 kb of two or more genes) [25], and during the evolution, strong selective purification pressures occurred, thus enabling different factors to regulate the MKK and MPK genes in *C. sinensis*.

### Functional interaction network of the MAPK proteins in tea plant

**Fig. 6:**
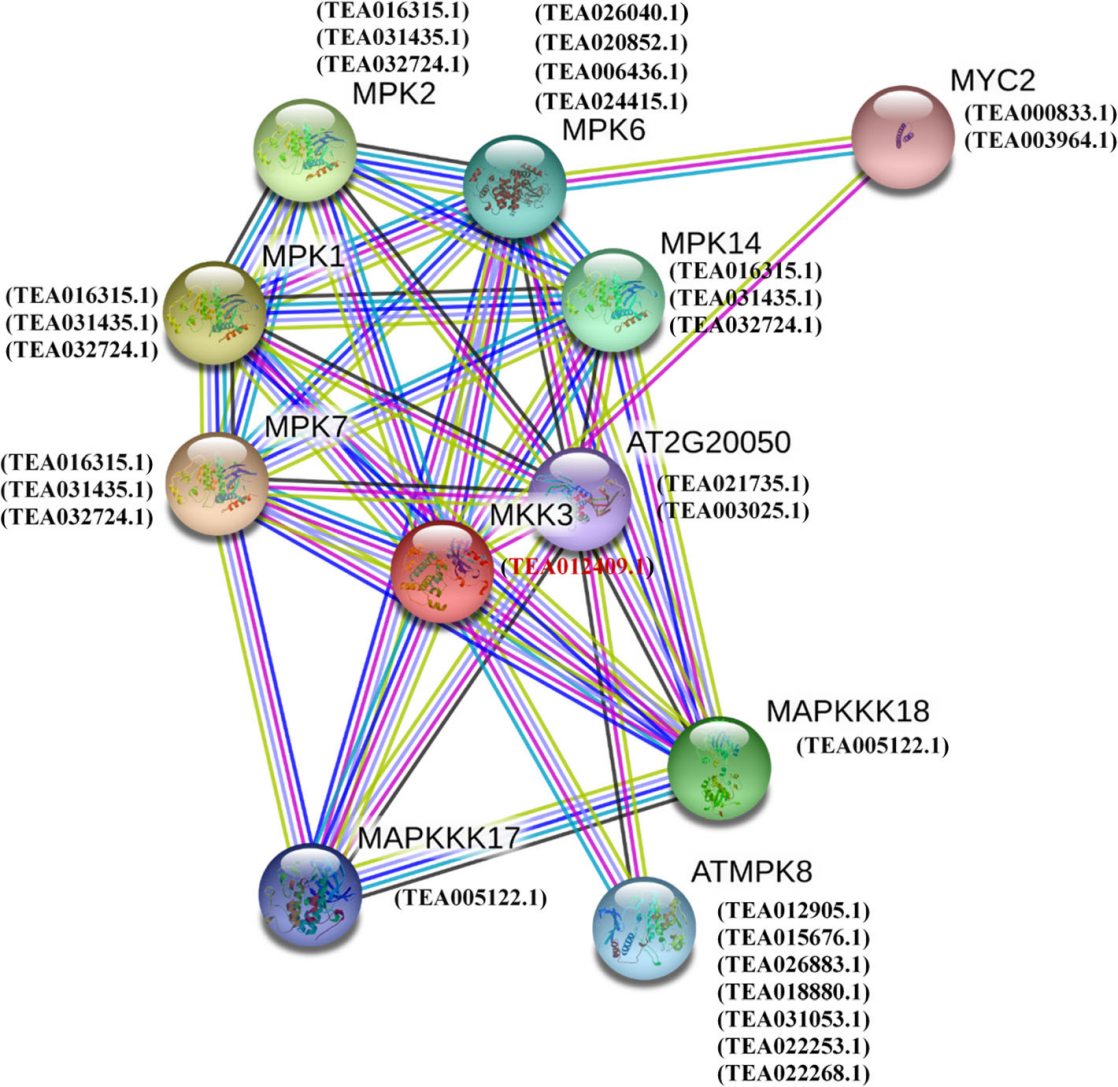
Functional interaction networks of MKK and MPK proteins. The interaction network was formed according to the ortholog in Arabidopsis. TEA012409.1 is orthologous to MKK3 in Arabidopsis. The orthologous protein (red) and homologous proteins (black) are shown within brackets

To understand the interactions of MKK and MPK genes in *C. sinensis*, an interaction network was constructed using the STRING software based on the orthologous genes in *Arabidopsis thaliana* (Fig. 6). MKK3 in Tea (TEA012409.1) is orthologous to MKK3 in Arabidopsis (AT5G40440) (http://tpia.teaplant.org/). Additionally, the tea proteins incorporated in the Fig. 6 were homologous to the Arabidopsis proteins participating in the interaction network. These homologous proteins were considered as STRING proteins and were selected based on high bit score. Widely prevalent similarity searching programs such as BLAST produce accurate statistical estimates and ensure that protein sequences sharing significant similarity also have similar structures [32]. In general, proteins with high sequence and structural similarity tend to possess similar functions [33]. MKK3 encodes for a Mitogen Activated Protein Kinase Kinase, which further activates MPK8, and is a target of MPKKK20. MKK3-MPK8 module acts as a negative regulator of ROS accumulation by regulating RBOHD gene expression. MKK3, MAPKKK17 and MAPKKK18 are a component of ABA signaling pathway which may act as an ABA signal transducer concerning abiotic stress. Also, MKK3-MPK7 module plays a vital role as a positive regulator of PR1 gene expression. MAPKKK17 and MAPKKK18 belong to Ser/Thr protein kinase family and help in the ABA-dependent activation of the MKK3-MPK7 pathway. It triggers MPK6 activation in an MKK3-independent manner and MPK1, MPK2, MPK7 and MPK14 activation in an MKK3-dependent manner. This gene is also a positive regulator of ABA responses, leads to the induction of gene expression and involved in various responses such as stomatal development movement, inhibition of germination as well as root growth, and promotes leaf senescence.

### Tissue specific developmental gene expression of tea MAPKs

**Fig. 7:**
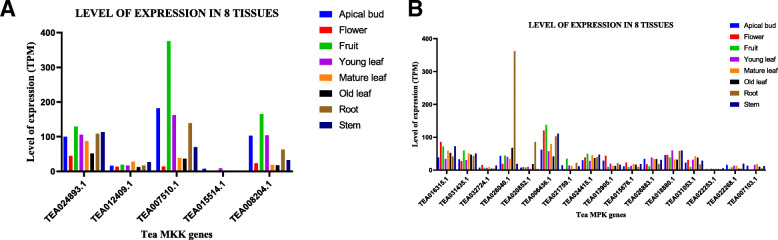
Tissue-specific expression patterns of (**a**) MKK and (**b**) MPK genes in *C. sinensis***.** The expression of these genes is shown in transcripts per million (TPM) during different developmental stages. GraphPad Prism 8 was used to generate the graphs

The gene expression pattern for MKK genes in various tissues (apical bud, flower, fruit, young leaf, mature leaf, old leaf, root, stem) were retrieved from TPIA database wherein the level of expression was evaluated using transcripts per million (TPM). Among the 5 MKK and 16 MPK genes analysed, different levels of expression were observed and transcripts were barely detectable in few cases (Fig. 7a and b). Among the 5 MKK genes, TEA007510.1 of MKK clade D was present in higher abundance in the apical bud, fruit, young leaf, and root when compared to the other MKKs (Fig. 7a). On the other hand, TEA024893.1 of MKK clade A, showed the highest level of expression in flower, mature leaf, old leaf and stem (Fig. 7a). In contrast, the expression of TEA015514.1 of clade A was barely detected in all the tissues (Fig. 7a). This MKK gene showed negligible expression in mature leaf and old leaf, and its expression was not detected in fruit (Fig. 7a). Similarly, the patterns of gene expression for MPK genes in various tissues (apical bud, flower, fruit, young leaf, mature leaf, old leaf, root, and stem) were also retrieved from TPIA database wherein again, the level of expression was evaluated using TPM (Fig. 7b). In apical bud, flower, fruit, mature leaf and stem, TEA006436.1 of MPK clade A, was expressed at higher levels than the other MPK genes (Fig. 7b). Further, the expression of TEA18880.1 of MPK clade F in young leaves, as well as TEA026040.1 of MKK clade B in old leaf and root, was observed to be significantly high (Fig. 7b). Out of the studied 16 MPK genes, TEA022253.1 of clade F, showed relatively very low expression levels in all the tissues (Fig. 7 b).

### Cold, drought and salt stress induced differential expression of tea MPKs

**Fig. 8:**
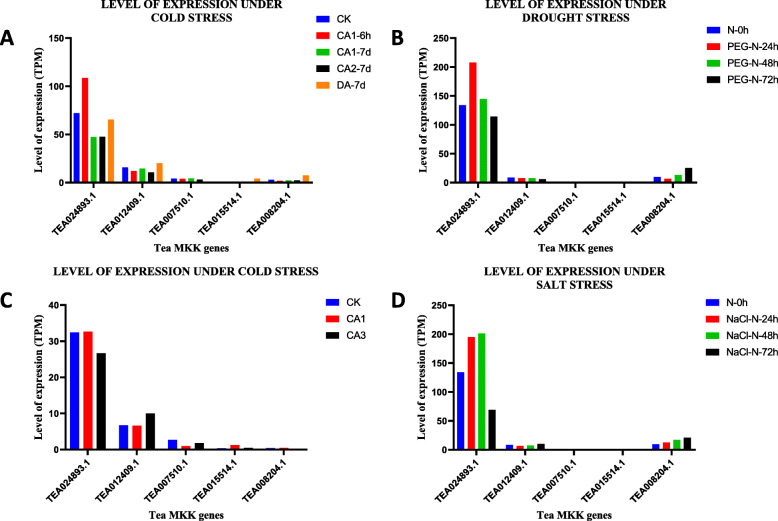
Expression pattern of MKK genes in response to (**a**) cold stress (unpublished) (**b**) drought treatment (**c**) cold stress (published) (**d**) salt treatment in *C. sinensis*. The expression of these genes is shown in transcripts per million (TPM) during different developmental stages. GraphPad Prism 8 was used to generate the graphs

**Fig. 9:**
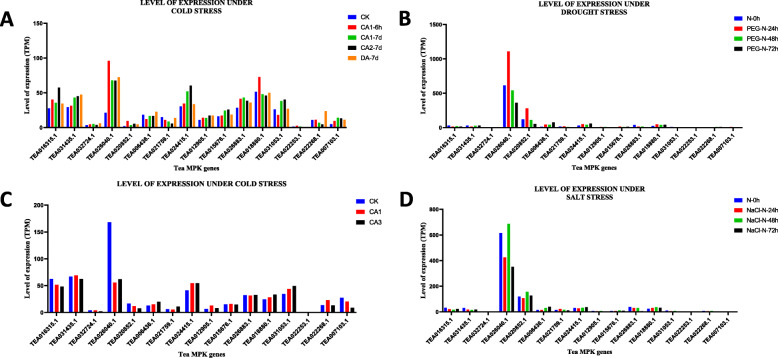
Expression pattern of MPK genes in response to (**a**) cold stress (unpublished) (**b**) drought treatment (**c**) cold stress (published) (**d**) salt treatment in *C. sinensis*. The expression of these genes is shown in transcripts per million (TPM) during different developmental stages. GraphPad Prism 8 was used to generate the graphs

To the check influence of cold, drought and salt stress on 5 MKK and 16 MPK genes, the expression data was retrieved from the TPIA database (Fig. 8 and Fig. 9) and the level of expression was found to be evaluated using TPM. The retrieved cold acclimated (published) data had 3 stages- 1. Non-acclimated (CK), 2. Fully acclimated (CA1) and 3. De-acclimated (CA3) [34]. In the MKK expression data, CK was taken as control and in CA1, the expression of only 2 out of 5 genes was up-regulated whereas the rest of the 3 MKK genes showed down-regulation. Further, under CA3 condition, out of 5 genes, only 1 gene was up-regulated (TEA012409.1), 1 did not show any expression (TEA008204.1), and the other 3 genes were down-regulated (TEA024893.1, TEA007510.1, TEA015514.1) (Fig. 8c). A similar approach was carried out for MPK genes. The results showed that out of 16 genes, the expression of 7 genes was up-regulated, 8 genes were down-regulated and negligible expression was observed by 1 gene in both CA1 and CA3 condition, wherein CK was the control (Fig. 9c).

To further demonstrate the cold tolerance in tea plant, another cold acclimated data (unpublished), which was retrieved from the TPIA database, comprised of five stages of expression: 1. 25 ~ 20 °C (CK), 2. Fully acclimated at 10 °C for 6 h (CA1–6 h) 3. 10 ~ 4 °C for 7 days (CA1-7d), 4. Cold response at 4 ~ 0 °C for 7 days (CA2-7d) and 5. Recovering under 25 ~ 20 °C for 7 days (DA-7d), where CK was the control [35]. Therefore, the expression of MKK genes under CA1–6 h expression showed that only 1 gene out of 5 was up-regulated and rest of the 4 genes were down-regulated. Further, for CA1-7d condition, the expression showed that 2 out of 5 genes showed up-regulation whereas the other 3 genes showed down-regulation. For CA2-7d, the expression of all the 5 genes was down-regulated and for DA-7d, the expression of 3 genes was seen to be up-regulated, 1 gene did not show any expression and the remaining 1 gene was down-regulated (Fig. 8a). Taking the above conditions into consideration, the expression analysis of MPK genes was carried out. Here, the expression of 13 genes was up-regulated and 3 genes were down-regulated in CA1–6 h condition. Further, under CA1-7d condition, 11 genes showed up-regulation and rest 5 genes showed down-regulation. When these MPK genes were studied in CA2-7d condition, results showed that 11 genes were up-regulated and 5 genes were down-regulated. In the DA-7d condition, 13 genes were up-regulated and rest of the genes showed down regulation (Fig. 9a).

In addition, the transcriptomic data from TPIA was analysed to show the expression of MAPK genes under drought stress. A total of 5 genes of MKK in 25% polyethylene glycol (PEG) treatment were detected to be expressed under drought stress, which included four stages: 0, 24, 48 and 72 h [36], where 0 h was taken as the control. Results showed that in PEG-N-24 h, the expression of only 2 genes was up-regulated and that of 3 genes was down-regulated. For PEG-N-48 h, the expression of 2 genes was up-regulated and that of another 3 genes was down-regulated. Further, down-regulation of 2 out of 5 genes and up-regulation of 3 genes was observed in PEG-N-72 h (Fig. 8b). To further understand the expression of MPK genes under drought stress, a similar approach was considered. In this study, the results in PEG-N-24 h condition showed that out of 16 genes, 9 genes were up-regulated, 1 gene showed negligible expression and 6 genes were down-regulated. Under PEG-N-48 h and PEG-N-72 h conditions, 7 genes were up-regulated and 8 genes were down-regulated. Further in these (PEG-N-48 h and PEG-N-72 h) conditions, 1 gene (TEA022253.1) had shown negligible expression (Fig. 9b).

Meanwhile, the expression pattern of MKK genes under salt stress was also studied, and this expression data was collected from the TPIA database. Here 0, 24, 48, 72 h of salt stress conditions in tea plants were stimulated with 200 mM NaCl. Salt stress resulted in up-regulation of 3 out of 5 genes and rest of the 2 genes were down-regulated when studied under NaCl-N-24 h condition. In the case of NaCl-N-48 h, the expression of 4 genes was up-regulated and only 1 gene was down-regulated (Fig. 8d). Similarly, under NaCl-N-72 h condition, 2 genes showed up-regulation, 1 out of 5 genes showed down-regulation and 2 genes showed negligible expression. For all the conditions, N-0 h was taken as control. Taking similar methods in consideration NaCl-N-24 h showed that 3 genes were up-regulated, 12 genes were down-regulated and 1 gene did not show any expression. Under NaCl-N-48 h, out of 16 genes, 9 genes were up-regulated, 6 genes were down-regulated, and 1 gene showed negligible expression. Finally, for NaCl-N-72 h treatment, 5 genes were up-regulated, the expression of 1 gene was barely detectable and 10 genes were down-regulated (Fig. 9d).

## Discussion

For several plants such as Arabidopsis, Brachypodium, poplar, rice, apple and so on, MAPK and MAPKK gene families have already been identified and functionally characterized [15, 37–41]. However, the MAPK and MAPKK genes in *C. sinensis* had not been studied. This study provided a detailed overview of the gene structure, phylogenetic relationship, genomic distribution and expressions at the genome level of MAPK and MAPKK genes in *C. sinensis*. The study of intron / exon architecture in the *C. sinensis* genes showed that three out of five MKK genes (60% of the Tea MKK gene) have 6 to 8 introns, and the two other genes in clade D of the MKK gene family, have no introns. The longest MKK gene in *C. sinensis* with 8 introns is also found in TEA12409.1. In fact, 44% of the MPK genes had 10 or more introns and these genes belonged to clade A, F, G, or H. Generally, the MPK genes belonging to the same clade had similar number of exons and introns. Only TEA007103.1 showed variation with respect to number of introns and exons, in comparison to the other genes in its clade. Additionally, information has been provided regarding exon/intron length, UTRs and gene length. This study of gene structure indicated variations in both number and length of introns as well as exons, resulting in gene length variation.

These MAPK modules comprise at least three protein kinases, which undergo phosphorylation sequentially and essentially require phosphorylation of both serine and threonine for complete activity [42]. These conserved serine and threonine residues are found close to kinase domain VII of all MAPKs [43, 44]. In plants, MAPKs have not evolved into different variants for the activation loops and either glutamic acid (TEY) or similar aspartic acid (TDY) is the key residue [43, 45]. In this MAP kinase trigger-mechanism, on activation of the MAPKK, signal transduction occurs through the downstream activation of MAPKK by phosphorylating serine and serine/threonine residues in the S/T X5 S/T motif, which triggers its downstream MAPK when threonine and tyrosine residues in T-X-Y motif of T loop are phosphorylated [4, 46]. In this study, the “TEY” motif of T-loop is only present among the MPK genes of clades A, B and C, with it being modified to “TDY” in all the MPK genes of other clades. The activation loop of the MKKs is preceded by the profoundly conserved “DFGx” sequence, a third S/T is also present near the activation loop and five amino acid residues downstream the S/T region.

Previous comprehensive studies in plants have revealed that MAPK cascades are extensively involved in controlling a number of biological processes including cell growth, proliferation and response to various biotic and abiotic stresses such as salt stress, cold stress and drought stress [8, 47, 48]. The AtMPK3, AtMPK4 and AtMPK6 in Arabidopsis as well as their orthologs in other plant species, are triggered majorly by various stimuli including abiotic and oxidative stress [10, 49]. MAPK proteins classified in the same clades have been reported to perform similar roles in different organisms [38, 49]. MAPKs and MAPKKs in *C. sinensis* can be categorized in ten (clade A, B, C, D, E, F, G, H, I and J) and four (clade A, B, C and D) different subfamilies respectively, on the basis of the homology of sequences. AtMKK3 has a crucial role in responsiveness to salt stress [50]. In this study, TEA021409.1 of clade B is found to be homologous and orthologous to AtMKK3. Additionally, the selected tea proteins were homologous to the Arabidopsis proteins participating in the interaction network. These homologous proteins were considered as STRING proteins because proteins with high sequence and structural similarity tend to possess similar functions. Therefore, the interaction network was built to predict the interactions of MAPK genes in tea plant.

Biochemical and genetic research suggests that MKK2 is critically significant in the response to cold stress and salt stress in Arabidopsis [51]. The expression data presented in this study suggests that TEA024893.1 among tea MKK genes, and TEA026040.1 among tea MPK genes, show the highest level of expression under cold stress (published and unpublished), salt stress as well as drought stress. The highly reactive by-products in the aerobic metabolism are the reactive oxygen species (ROS) which are the oxygen derivatives (H_2_O_2_, HO^**.**^, ^1^O_2_, O_2_^−^), reduced or triggered partially [10]**.** A complicated ROS network of ROS enzymes along with antioxidant enzymes and antioxidants enables the plant to regulate ROS levels [10]**.** Excess ROS can lead to oxidative damage and cell death as it is a toxic by-product of various chemical reactions [10]**.** To this end, the plants have built effective strategies for targeted ROS production and ROS regulating mechanisms [10]. Studies suggest that multiple stresses such as salt stress, cold stress and drought stress stimulate ROS production, which in turn triggers MAPK signalling cascades [11]. It has been revealed that MPKKK1 activates two of its highly homologous MAPKKs (MKK1 and MKK2), which operate upstream of both MPK4 and MPK6 [46, 51]. All these kinases were involved in biotic, abiotic and ROS signalling [10, 52]. The majority of plant genes in MAPK cascade responded to abiotic stress, with some genes indicating their involvement in the development. AtMKK6 plays a crucial role in cytokinesis and cell death [52]. In this study, the phylogenetic tree determines that TEA015514.1 has close phylogenetic relationship with AtMKK6. It was also revealed that AtMPK3/AtMPK6 was required in Arabidopsis, not in its development, but to direct the pollen tubes [53]. Previous research has shown that in Arabidopsis, MPK18 participates in microtubular functions [54, 55]. In our study, TEA18880.1 and TEA022253.1 belong to clade F, and are homologous to AtMPK18. And specifically, TEA18880.1 had shown significantly high expression in young leaves. Overall, this study provides the comprehensive analysis of the MKK and MPK genes in *C. sinensis*. Therefore, the increasing studies on these genes in *C. sinensis* can contribute to a deeper understanding of their various roles in developmental processes as well as their expression under abiotic stresses, which needs to be elucidated by further research.

## Conclusion

MAPK cascade genes encode a set of plant-specific ROS scavenging enzymes described and characterized in multiple plant species. There has been considerable progress in identifying the roles of MPK and MKK genes in many organisms, but these genes have not been studied in *C. sinensis*. This study aims at determining the genomic stage of the MKK and MPK genes in tea plant. In total, 5 MKK and 16 MPK genes were obtained, which were further assisted by the existence of conserved domains and signature motifs. From the phylogenetic relationship, the neighbor joining (NJ) trees were divided into 4 and 10 clades for MKK and MPK respectively. Distribution of homologous gene pairs and bootstrapping supported the pattern of grouping. The plant species taken for comparative study clearly indicated segmental duplication, which was a major candidate in the expansion of MAPK cascades. But due to their near distribution, tandem duplication was missing in the 5 MKK and 16 MPK genes.

The MKK and MPK genes were successfully mapped onto the scaffold by sourcing out the information publicly available in the TPIA database. The functionally interacting orthologous groups were also analysed. In addition, to demonstrate the possible roles of MKK and MPK genes, a comparative study of these gene expression profiles were studied in different tissues. Also, the expression profiles of these MKK and MPK genes were studied under abiotic stresses. Tea is an economic crop in which these MPK and MKK genes were not studied. Hence, this work will provide a basis to further study and explore the role of MAPK and MAPKK genes in various developmental processes and also elucidate the other potential functions of these genes in *C. sinensis*.

## Methods

### Identification of MPK and MKK genes of tea plant

The genome sequence of the tea plant was retrieved from the Tea Plant Information Archive, TPIA [35] (http://tpia.teaplant.org/). Then, the MKK and MPK genes of Arabidopsis retrieved from the TAIR database [56] (https://www.arabidopsis.org/), were used as queries to search against the tea genome database using BLASTp algorithm with an e-value of 1e-5 and identity match of 50% as the threshold. Further, the protein sequences were subjected to MUSCLE (https://www.ebi.ac.uk/Tools/msa/muscle/) [57] for Multiple Sequence alignment, in order to obtain the conserved domains. The candidate genes were then submitted to SMART [58] (http://smart.embl-heidelberg.de/) and Pfam web tool to verify the presence of kinase domains. The physicochemical properties, which include the isoelectric point (pI), molecular weight (MW) and instability index of the cascade proteins, were predicted using ProtParam tool integrated in ExPASy database [59] (https://expasy.org/). Further to predict the subcellular localization of the protein sequences, the BaCelLo online server [60] (http://gpcr.biocomp.unibo.it/bacello/index.htm) was used. Also, TMHMM server v2.0 [61] (http://www.cbs.dtu.dk/services/TMHMM/) was used to predict the Trans-membrane helices in MPK and MKK proteins.

### Phylogenetic and evolutionary analysis of MPK and MKK genes

Multiple sequence alignment of the MPK and MKK protein sequences in the selected plants was carried out using MUSCLE tool integrated in MEGA7.0.14 [62], with the default parameters. The sequence alignments were also used for the phylogenetic analysis, and the trees were generated with MEGA7.0.14 software [62] by using the neighbor joining, maximum likelihood and minimum evolution algorithms. The reliability of the obtained trees was assessed with a bootstrap value of 1000. Also, the functional interaction network of *C. sinensis* was studied based on orthologous genes in *A. thaliana* by using STRING online server [63] (https://string-db.org/). The orthologous gene was found using TPIA database and further the homologous proteins were also considered as STRING proteins based on the high bit score.

### Conserved motifs and gene structure analysis

MEME suite [64] (http://meme-suite.org/) was employed for identification of the conserved motifs with default parameters**.** Both MKK and MPK gene sequences along with corresponding coding sequences were submitted to the Gene Structure Display Server v2.0 [65] (http://gsds.cbi.pku.edu.cn/), in order to obtain the structural details of intron/exon.

### Genomic distribution of MKK and MPK genes

The MKK and MPK genes were located on the corresponding scaffolds due to the incomplete genome assembly information available in the TPIA database. The genes were mapped onto the scaffolds by using MapGene2chromosome web v2 (MG2C) software tool [66] (http://mg2c.iask.in/mg2c_v2.0/), according to their positional information which includes: scaffold length and number, gene ID, starting and ending position of the genes as well as scaffold ID present in the TPIA database.

### Estimation of synonymous (dN) and non-synomymous (dS) substitutions per site and their ratio (dS/dN)

HIV sequence data (www.hiv.lanl.gov/content/sequence/ALIGN_MULTITOOL/align_mt.html) was used to align the coding sequences of MKK and MPK genes. MKK and MPK gene pairs were compared to estimate dN, dS and their ratio, which was calculated by SNAP v2.1.1 Synonymous Non-synonymous Analysis Program [67] (https://www.hiv.lanl.gov/content/sequence/SNAP/SNAP.html). The values of dS/dN display the selective pressure of duplicate genes, and the values of dS represents the time of divergence for duplication events.

### Expression profile and co-expression networks construction

The tissue expression data (apical bud, flower, fruit, young leaf, mature leaf, old leaf, root, stem) and the abiotic expression data (cold, drought, salt) under different parameters, were retrieved from tea plant information archive (tpia.teaplant.org/) (http://tpia.teaplant.org/download.html) [35]. Then, the MAPK cascade genes were initially searched against the Tea database using the gene accession number of each of the genes. Further, the expression data was plotted using GraphPad Prism 8 to generate the graphs separately for each of the stress conditions taken into consideration, in our study.

## Supplementary information

**Additional file 1: Table S1.**. BLAST positives table for MKK genes of *Camellia sinensis.* Arabidopsis MKK sequences, retrieved from TAIR database, were used as queries to search against TPIA database to identify the putative tea MKK genes. **Table S2**. BLAST positives table for MPK genes of *Camellia sinensis.* Arabidopsis MPK sequences, retrieved from TAIR database, were used as queries to search against TPIA database to identify the putative tea MPK genes. **Table S3**. Subcellular localization of the 5 MKK proteins. BaCelLo web server was used to predict the localization. Here, the localization steps indicate where the gene is localized and expressed in the plant body. **Table S4**. Subcellular localizations of the 16 MPK proteins. BaCelLo web server was used to predict the localization. Here, the localization steps indicate where the gene is localized and expressed in the plant body. **Table S5.** The dS/dN ratio of MKK genes in *C. sinensis*. This ratio is calculated to comprehend the selection pressure. The SNAP server (https://www.hiv.lanl.gov/content/sequence/SNAP/SNAP.html) has been used to generate these values. **Table S6.** The dS/dN ratio of MPK genes in *C. sinensis*. This ratio is calculated to comprehend the selection pressure. The SNAP server (https://www.hiv.lanl.gov/content/sequence/SNAP/SNAP.html) has been used to generate these values.

**Additional file 2: Figure S1**. Transmembrane helices data for the 5 MKK proteins in *Camellia sinensis.* TMHMM Server, v.2.0 (http://www.cbs.dtu.dk/services/TMHMM/), was used to predict the presence of transmembrane helices. **Figure S2.** Transmembrane helices data for the 16 MPK genes in *Camellia sinensis.* TMHMM Server, v.2.0 (http://www.cbs.dtu.dk/services/TMHMM/), was used to predict the presence of transmembrane helices. **Figure S3.** Phylogenetic analysis of MKK (A) and MPK (B) proteins by the minimum evolution (ME) method. The analysis was carried out among *C. sinensis* (black square), *A. thaliana* (black circle), *O. sativa* (black triangle), *S. lycopersicum* (black rhombus), *S. tuberosum* (red circle), *C. annum* (blue triangle), and *C. canephora* (grey triangle). The full-length MKK and MPK protein sequences were aligned using MUSCLE tool, and the phylogenetic tree was constructed using MEGA 7.0.14 by the minimum evolution (ME) method with default parameters and 1000 bootstrap replicates. **Figure S4.** Phylogenetic analysis of MKK (A) and MPK (B) proteins by the maximum likelihood (ML) method. The analysis was carried out among *C. sinensis* (black square), *A. thaliana* (black circle), *O. sativa* (black triangle), *S. lycopersicum* (black rhombus), *S. tuberosum* (red circle), *C. annum* (blue triangle), and *C. canephora* (grey triangle). The full-length MKK and MPK protein sequences were aligned using MUSCLE tool, and the phylogenetic tree was constructed using MEGA 7.0.14 by the maximum likelihood (ML) method with default parameters and 1000 bootstrap replicates. **Figure S5**. The motif logos of for MKK protein sequences. The motif logos were generated by MEME suite. The motif logo represents the conserved amino acid residues in the protein sequences. **Figure S6**. The motif logos of for MPK protein sequences. The motif logos were generated by MEME suite. The motif logo represents the conserved amino acid residues in the protein sequences.

**Additional file 3: Figure S7**: Alignment of domains in MPKs. All the MPK protein sequences were subjected to alignment by MUSCLE tool owing to their sequence diversities. Sequences that are highlighted are ATP binding signature, marked in blue, the catalytic C loop, marked in light red colour, the activation T loop, marked in green colour, CD domain, marked in light blue colour. Clades D to J show sequence derivations from the T(E/D) Y activation loop and are marked in a lighter shade of green colour.

**Additional file 4: Figure S8.** Alignment of domains in MKKs. All the MKK protein sequences were subjected to alignment by MUSCLE tool owing to their sequence diversities. Sequences that are highlighted are ATP binding signature, marked in blue that consists the P loop consensus sequence (GxGxxG), the catalytic C loop, marked in light red colour that consists the DΨK consensus, the activation T loop, marked in green colour and NTF2 domain marked in greyish colour. Clade C and D show sequence derivations from the S/TxxxxxS/T activation loop and are marked in a lighter shade of green colour.

## Data Availability

All data generated or analysed during this study are included in this article and in its Additional files. The accession numbers of MKK and MPK genes used for this study have been listed below: **1. Tea** (http://tpia.teaplant.org/)**:** **MKK:** TEA024893.1, TEA012409.1, TEA007510.1, TEA015514.1, TEA008204.1; **MPK:** TEA016315.1, TEA031435.1, TEA032724.1, TEA026040.1, TEA020852.1, TEA006436.1, TEA021759.1, TEA024415.1, TEA012905.1, TEA015676.1, TEA026883.1, TEA018880.1, TEA031053.1, TEA022253.1, TEA022268.1, TEA007103.1; **2. Arabidopsis** (https://www.arabidopsis.org/)**:** **MKK:** AT4G26070, AT4G29810, AT5G40440, AT1G51660, AT3G21220, AT5G56580, AT1G18350, AT3G06230, AT1G73500, AT1G32320; **MPK:** AT1G10210, AT1G59580, AT3G45640, AT4G01370, AT4G11330, AT2G43790, AT2G18170, AT1G18150, AT3G18040, AT3G59790, AT1G01560, AT2G46070, AT1G07880, AT4G36450, AT1G73670, AT5G19010, AT2G01450, AT1G53510, AT3G14720, AT2G42880; **3. Rice** (https://rapdb.dna.affrc.go.jp/)**:** **MKK:** Os06t147800–01, Os06t0473200–01, Os06t0473200–02, Os02t0787300–01, Os06t0191300–01, Os06t0191300–02, Os01t0510100–01, Os02t069400–00; **MPK:** Os06t0154500–01, Os08t157000–00, Os02t148100–01, Os06t0699400–01, Os03t0285800–01, Os06t154500–01, Os05t0566400–01, Os01t0665200–01, Os01t0665200–03, Os05t0582400–01, Os06t078000–01, Os06t078000–02, Os06t078000–04, Os02t0135200–00, Os05t0143500–02, Os11t0271100–01, Os01t0643800–01; **4. Tomato** (https://solgenomics.net/)**:** **MKK:** Solyc03g097920.1.1, Solyc03g123800.1.1, Solyc03019850.2.1, Solyc12g009020.1.1; **MPK:** Solyc06g068990.2.1, Solyc12g040680.1.1, Solyc01g080240.2.1, Solyc04g007710.2.1, Solyc05g008020.2.1, Solyc07g056350.2.1, Solyc10g007500.2.1, Solyc07g062080.2.1, Solyc04g080730.2.1, Solyc02g084870.2.1, Solyc06g005170.2.1, Solyc11g072630.1.1, Solyc08g081490.2.1, Solyc01g094960.2.1; **5. Potato** (https://solgenomics.net/)**:** **MKK:** PGSC0003DMP400056215, PGSC0003DMP400016192, PGSC0003DMP400026685, PGSC0003DMP400026683, PGSC0003DMP400026684, PGSC0003DMP400000549; **MPK:** PGSC0003DMP400036823, PGSC0003DMP400036824, PGSC0003DMP400012516, PGSC0003DMP400012517, PGSC0003DMP400030312, PGSC0003DMP400030311, PGSC0003DMP400030310, PGSC0003DMP400007748, PGSC0003DMP400007750, PGSC0003DMP400010570, PGSC0003DMP400010572, PGSC0003DMP400053050, PGSC0003DMP400004339, PGSC0003DMP400050161, PGSC0003DMP400006600, PGSC0003DMP400006283, PGSC0003DMP400052344, PGSC0003DMP400052345, PGSC0003DMP400044003, PGSC0003DMP400021542, PGSC0003DMP400000144; **6. Capsicum** (https://solgenomics.net/)**:** **MKK:** CA00g74230, CA00g92940, CA03g36820, CA03g22790, CA03g23860; **MPK:** CA08g07540, CA01g02140, CA11g13970, CA06g06270, CA02g22080, CA04g21490, CA10g01850, CA07g16590, CA05g08630, CA00g57490, CA06g17760, CA07g15380, CA12g10140, CA01g24290; **7. Coffee** (https://solgenomics.net/)**:** **MKK:** Cc04_g09350, Cc04_g00500, Cc00_g04720, Cc06_g11750. **MPK:** Cc00_g17870, Cc08_g15160, Cc01_g11870, Cc03_g14980, Cc01_g11790, Cc07_g10000, Cc03_g12110, Cc04_g06490, Cc11_g08590, Cc03_g02990, Cc05_g09440, Cc05_g10580.

## Abbreviations

MAPK/MPK: Mitogen Activated protein kinase
MPKK/MKK/MEK: Mitogen Activated Protein Kinase Kinase
MPKKK/MAPKKK/MEKK: Mitogen Activated Protein Kinase Kinase Kinase
ROS: Reactive-oxygen species
TPIA: Tea Plant Information Archive
CS/Cs: *Camellia sinensis*
TAIR: The Arabidopsis Information Resource
HMM: Hidden Markov Model
pI: Isoelectric point
MW: Molecular Weight
dN: Non-synonymous substitutions
dS: Synonymous substitutions
MEME: Multiple Expectation Maximization for Motif Elucidation
NJ: Neighbor Joining
ME: Minimum Evolution
ML: Maximum Likelihood
AT/At: *Arabidopsis thaliana*
CK: Non acclimated
CA1: Fully acclimated
CA3: De-acclimated
TPM: Transcripts per million
RBOHD: Respiratory burst oxidase homologue D
UTR: Untranslated Region
