# Supplementary material for: MAPK cascade gene family in Camellia sinensis: In-silico identification, expression profiles and regulatory network analysis
Source: BMC Genomics. 2020 Sep 7;21:613. doi: 10.1186/s12864-020-07030-x (PMC7487466; doi:10.1186/s12864-020-07030-x)
Supplement: Supplementary file 1 — Additional file 1: Table S1.. BLAST positives table for MKK genes of Camellia sinensis. Arabidopsis MKK sequences, retrieved from TAIR database, were used as queries to search against TPIA database to identify the putative tea MKK genes. Table S2. BLAST positives table for MPK genes of Camellia sinensis. Arabidopsis MPK sequences, retrieved from TAIR database, were used as queries to search against TPIA database to identify the putative tea MPK genes. Table S3. Subcellular localization of the 5 MKK proteins. BaCelLo web server was used to predict the localization. Here, the localization steps indicate where the gene is localized and expressed in the plant body. Table S4. Subcellular localizations of the 16 MPK proteins. BaCelLo web server was used to predict the localization. Here, the localization steps indicate where the gene is localized and expressed in the plant body. Table S5. The dS/dN ratio of MKK genes in C. sinensis. This ratio is calculated to comprehend the selection pressure. The SNAP server (https://www.hiv.lanl.gov/content/sequence/SNAP/SNAP.html) has been used to generate these values. Table S6. The dS/dN ratio of MPK genes in C. sinensis. This ratio is calculated to comprehend the selection pressure. The SNAP server (https://www.hiv.lanl.gov/content/sequence/SNAP/SNAP.html) has been used to generate these values. [file 12864_2020_7030_MOESM1_ESM.docx]

**MAPK cascade gene family in *Camellia sinensis*: *In-silico* identification, expression profiles and regulatory network analysis**

Archita Chatterjee^1*^, Abhirup Paul^1*^, G Meher Unnati^1*^, Ruchika Rajput^3^, Trisha Biswas^1^, Tamalika Kar^1^, Srijita Basak^1^, Neelam Mishra^2^, Ashutosh Pandey^3†^, Anurag P. Srivastava^1†^

^1^Department of Life Sciences

Garden City University

Bangalore, Karnataka,

India

^2^Department of Botany

St. Joseph’s College

Bangalore, Karnataka,

India

^3^National Institute of Plant Genome Research

Aruna Asaf Ali Marg, New Delhi,

India

*These authors contributed equally to this work.

†Corresponding Author,

Phone: +91-9108720555

Anurag P. Srivastava: [anuiitkgp@gmail.com](mailto:anuiitkgp@gmail.com)

ORCID ID: 0000-0003-3493-1375

Ashutosh Pandey: [ashutosh@nipgr.ac.in](mailto:ashutosh@nipgr.ac.in)

Phone: 91-11-26741612,14,17 Ext.- 236

**Additional file 1**

**Supplementary Table 1**

**BLAST positives table for MKK genes of *Camellia sinensis.*** Arabidopsis MKK sequences, retrieved from TAIR database, were used as queries to search against TPIA database to identify the putative tea MKK genes.

| **AtMKK** | **CsMKK** | **Identities** | **Positives** | **Gaps** |
| --- | --- | --- | --- | --- |
| AT4G26070 (MKK1) | TEA024893.1 | 66% | 82% | 1% |
| AT4G29810 (MKK2) | TEA024893.1 | 69% | 81% | 2% |
| AT5G40440 (MKK3) | TEA012409.1 | 79% | 87% | 2% |
| AT1G51660 (MKK4) | TEA007510.1 | 54% | 73% | 2% |
| AT3G21220 (MKK5) | TEA007966.1 | 70% | 80% | 4% |
| AT5G56580 (MKK6) | TEA015514.1 | 77% | 86% | 4% |
| AT1G18350 (MKK7) | TEA007510.1 | 60% | 75% | 4% |
| AT3G06230 (MKK8) | TEA007510.1 | 50% | 70% | 6% |
| AT1G73500 (MKK9) | TEA008204.1 | 64% | 79% | 3% |
| AT1G32320(MKK10) | TEA007510.1 | 44% | 60% | 4% |

**Supplementary Table 2**

**BLAST positives table for MPK genes of *Camellia sinensis.*** Arabidopsis MPK sequences, retrieved from TAIR database, were used as queries to search against TPIA database to identify the putative tea MPK genes.

| **AtMPK** | **CsMPK** | **Identities** | **Positives** | **Gaps** |
| --- | --- | --- | --- | --- |
| AT1G10210 (MPK1) | TEA016315.1 | 85% | 94% | 0% |
|  | TEA031435.1 | 75% | 83% | 12% |
|  | TEA032724.1 | 79% | 91% | 0% |
| AT1G59580 (MPK2) | TEA016315.1 | 85% | 93% | 1% |
|  | TEA031435.1 | 76% | 83% | 12% |
|  | TEA032724.1 | 79% | 90% | 1% |
| AT3G45640 (MPK3) | TEA026040.1 | 80% | 91% | 0% |
|  | TEA020852.1 | 77% | 88% | 2% |
|  | TEA006436.1 | 66% | 85% | 0% |
| AT4G01370 (MPK4) | TEA006436.1 | 88% | 94% | 0% |
|  | TEA021759.1 | 82% | 92% | 0% |
|  | TEA026040.1 | 67% | 83% | 0% |
|  | TEA020852.1 | 65% | 82% | 0% |
| AT4G11330 (MPK5) | TEA006436.1 | 77% | 89% | 0% |
|  | TEA021759.1 | 78% | 90% | 0% |
|  | TEA026040.1 | 67% | 84% | 0% |
|  | TEA020852.1 | 67% | 83% | 0% |
| AT2G43790 (MPK6) | TEA026040.1 | 78% | 90% | 1% |
|  | TEA020852.1 | 73% | 87% | 1% |
|  | TEA006436.1 | 71% | 85% | 0% |
|  | TEA024415.1 | 91% | 95% | 0% |
| AT2G18170 (MPK7) | TEA016315.1 | 83% | 93% | 0% |
|  | TEA031435.1 | 73% | 80% | 12% |
|  | TEA032724.1 | 82% | 92% | 0% |
| AT1G18150 (MPK8) | TEA012905.1 | 73% | 82% | 7% |
|  | TEA015676.1 | 74% | 81% | 10% |
|  | TEA026883.1 | 74% | 83% | 3% |
|  | TEA018880.1 | 69% | 84% | 2% |
|  | TEA031053.1 | 69% | 80% | 2% |
|  | TEA022253.1 | 73% | 88% | 1% |
|  | TEA022268.1 | 74% | 82% | 12% |
| AT3G18040 (MPK9) | TEA012905.1 | 79% | 89% | 1% |
|  | TEA015676.1 | 79% | 85% | 1% |
|  | TEA026883.1 | 82% | 91% | 2% |
|  | TEA018880.1 | 72% | 82% | 2% |
|  | TEA031053.1 | 77% | 90% | 0% |
|  | TEA022253.1 | 74% | 89% | 0% |
|  | TEA022268.1 | 75% | 82% | 12% |
| AT3G59790 (MPK10) | TEA006436.1 | 64% | 82% | 0% |
| AT1G01560 (MPK11) | TEA006436.1 | 86% | 92% | 0% |
|  | TEA021759.1 | 81% | 90% | 0% |
| AT2G46070 (MPK12) | TEA006436.1 | 79% | 90% | 0% |
|  | TEA021759.1 | 76% | 88% | 0% |
| AT1G07880 (MPK13) | TEA006436.1 | 74% | 85% | 0% |
|  | TEA007103.1 | 81% | 90% | 2% |
|  | TEA026040.1 | 66% | 83% | 0% |
|  | TEA021759.1 | 76% | 87% | 0% |
| AT4G36450 (MPK14) | TEA016315.1 | 78% | 90% | 1% |
|  | TEA031435.1 | 70% | 79% | 13% |
|  | TEA032724.1 | 78% | 89% | 1% |
| AT1G73670 (MPK15) | TEA012905.1 | 71% | 83% | 3% |
|  | TEA015676.1 | 73% | 82% | 8% |
|  | TEA026883.1 | 71% | 84% | 4% |
|  | TEA018880.1 | 71% | 84% | 2% |
|  | TEA022253.1 | 73% | 88% | 0% |
|  | TEA031053.1 | 70% | 84% | 3% |
|  | TEA022268.1 | 72% | 80% | 12% |
| AT5G19010 (MPK16) | TEA026883.1 | 78% | 86% | 5% |
|  | TEA031053.1 | 71% | 78% | 9% |
|  | TEA012905.1 | 71% | 84% | 1% |
|  | TEA018880.1 | 64% | 74% | 8% |
|  | TEA015676.1 | 63% | 73% | 9% |
|  | TEA022253.1 | 77% | 90% | 0% |
|  | TEA022268.1 | 70% | 82% | 12% |
| AT2G01450 (MPK17) | TEA012905.1 | 70% | 83% | 1% |
|  | TEA015676.1 | 67% | 77% | 9% |
|  | TEA026883.1 | 64% | 79% | 8% |
|  | TEA018880.1 | 71% | 87% | 1% |
|  | TEA022253.1 | 70% | 88% | 1% |
|  | TEA031053.1 | 67% | 81% | 3% |
|  | TEA022268.1 | 70% | 79% | 13% |
| AT1G53510 (MPK18) | TEA018880.1 | 69% | 79% | 6% |
|  | TEA022253.1 | 81% | 93% | 0% |
|  | TEA026883.1 | 69% | 81% | 6% |
|  | TEA012905.1 | 73% | 88% | 0% |
|  | TEA015676.1 | 64% | 76% | 8% |
|  | TEA031053.1 | 58% | 71% | 8% |
|  | TEA022268.1 | 67% | 80% | 12% |
| AT3G14720 (MPK19) | TEA018880.1 | 71% | 80% | 4% |
|  | TEA022253.1 | 83% | 94% | 0% |
|  | TEA026883.1 | 65% | 78% | 4% |
|  | TEA012905.1 | 76% | 89% | 0% |
|  | TEA031053.1 | 61% | 73% | 5% |
|  | TEA015676.1 | 65% | 77% | 10% |
|  | TEA022268.1 | 70% | 80% | 13% |
| AT2G42880 (MPK20) | TEA018880.1 | 67% | 78% | 5% |
|  | TEA026883.1 | 68% | 79% | 5% |
|  | TEA012905.1 | 78% | 92% | 0% |
|  | TEA022253.1 | 81% | 94% | 0% |
|  | TEA015676.1 | 73% | 84% | 8% |
|  | TEA031053.1 | 66% | 77% | 6% |
|  | TEA022268.1 | 70% | 83% | 12% |

**Supplementary Table 3**

**Subcellular localizations of the 5 MKK proteins.** BaCelLo web server was used to predict the localization. Here, the localization steps indicate where the gene is localized and expressed in the plant body.

| **Gene ID** | **Localization** | **Localization steps** |
| --- | --- | --- |
| TEA024893.1 | Nucleus | (Intracellular -> Nucleus or Cytoplasm -> Nucleus) |
| TEA012409.1 | Nucleus | (Intracellular -> Nucleus or Cytoplasm -> Nucleus) |
| TEA007510.1 | Chloroplast | (Intracellular -> Organelle -> Chloroplast) |
| TEA015514.1 | Nucleus | (Intracellular -> Nucleus or Cytoplasm -> Nucleus) |
| TEA008204.1 | Chloroplast | (Intracellular -> Organelle -> Chloroplast) |

**Supplementary Table 4**

**Subcellular localizations of the 16 MPK proteins.** BaCelLo web server was used to predict the localization. Here, the localization steps indicate where the gene is localized and expressed in the plant body.

| **Gene ID** | **Localization** | **Localization steps** |
| --- | --- | --- |
| TEA016315.1 | Chloroplast | (Intracellular -> Organelle -> Chloroplast) |
| TEA031435.1 | Nucleus | (Intracellular -> Nucleus or Cytoplasm -> Nucleus) |
| TEA032724.1 | Nucleus | (Intracellular -> Nucleus or Cytoplasm -> Nucleus) |
| TEA026040.1 | Cytoplasm | (Intracellular -> Nucleus or Cytoplasm -> Cytoplasm) |
| TEA020852.1 | Cytoplasm | (Intracellular -> Nucleus or Cytoplasm -> Cytoplasm) |
| TEA006436.1 | Chloroplast | (Intracellular -> Organelle -> Chloroplast) |
| TEA021759.1 | Cytoplasm | (Intracellular -> Nucleus or Cytoplasm -> Cytoplasm) |
| TEA024415.1 | Nucleus | (Intracellular -> Nucleus or Cytoplasm -> Nucleus) |
| TEA012905.1 | Nucleus | (Intracellular -> Nucleus or Cytoplasm -> Nucleus) |
| TEA015676.1 | Nucleus | (Intracellular -> Nucleus or Cytoplasm -> Nucleus) |
| TEA026883.1 | Chloroplast | (Intracellular -> Organelle -> Chloroplast) |
| TEA018880.1 | Nucleus | (Intracellular -> Nucleus or Cytoplasm -> Nucleus) |
| TEA031053.1 | Nucleus | (Intracellular -> Nucleus or Cytoplasm -> Nucleus) |
| TEA022253.1 | Nucleus | (Intracellular -> Nucleus or Cytoplasm -> Nucleus) |
| TEA022268.1 | Nucleus | (Intracellular -> Nucleus or Cytoplasm -> Nucleus) |
| TEA007103.1 | Nucleus | (Intracellular -> Nucleus or Cytoplasm -> Nucleus) |

**Supplementary Table 5**

**The dS/dN ratio of MKK genes in *C. sinensis*.** This ratio is calculated to comprehend the selection pressure. The SNAP server (https://www.hiv.lanl.gov/content/sequence/SNAP/SNAP.html) has been used to generate these values.

| **Compare** | **Sequence names** | **Sd** | **Sn** | **S** | **N** | **ps** | **pn** | **dS** | **dN** | **ds/dn** | **ps/pn** |
| --- | --- | --- | --- | --- | --- | --- | --- | --- | --- | --- | --- |
| 0 1 | TEA007966.1 / TEA008204.1 | 166.8333 | 527.1667 | 237.1667 | 728.8333 | 0.7034 | 0.7233 | 2.0846 | 2.5016 | 0.8333 | 0.9725 |
| 0 2 | TEA007966.1 / TEA012409.1 | 165.8333 | 592.1667 | 220.8333 | 745.1667 | 0.7509 | 0.7947 | nan | nan | nan | 0.945 |
| 0 3 | TEA007966.1 / TEA015514.1 | 180.1667 | 561.8333 | 227.5 | 738.5 | 0.7919 | 0.7608 | nan | nan | nan | 1.041 |
| 0 4 | TEA007966.1 / TEA024893.1 | 163.3333 | 564.6667 | 227 | 739 | 0.7195 | 0.7641 | 2.4025 | nan | nan | 0.9417 |
| 1 2 | TEA008204.1 / TEA012409.1 | 179.8333 | 560.1667 | 209.1667 | 765.8333 | 0.8598 | 0.7314 | nan | 2.7746 | nan | 1.1754 |
| 1 3 | TEA008204.1 / TEA015514.1 | 169 | 574 | 215.5 | 759.5 | 0.7842 | 0.7558 | nan | nan | nan | 1.0377 |
| 1 4 | TEA008204.1 / TEA024893.1 | 172 | 556 | 215.3333 | 759.6667 | 0.7988 | 0.7319 | nan | 2.7931 | nan | 1.0914 |
| 2 3 | TEA012409.1 / TEA015514.1 | 775.5 | 3015.5 | 1071.5 | 4145.5 | 0.7238 | 0.7274 | 2.5144 | 2.6271 | 0.9571 | 0.995 |
| 2 4 | TEA012409.1 / TEA024893.1 | 998.1667 | 3912.833 | 1391.167 | 5379.833 | 0.7175 | 0.7273 | 2.3542 | 2.6238 | 0.8973 | 0.9865 |
| 3 4 | TEA015514.1 / TEA024893.1 | 760.5 | 3049.5 | 1061.667 | 4155.333 | 0.7163 | 0.7339 | 2.3275 | 2.8798 | 0.8082 | 0.9761 |

**Supplementary Table 6**

**The dS/dN ratio of MPK genes in *C. sinensis*.** This ratio is calculated to comprehend the selection pressure. The SNAP server (https://www.hiv.lanl.gov/content/sequence/SNAP/SNAP.html) has been used to generate these values.

| **Compare** | **Sequence name** | **Sd** | **Sn** | **S** | **N** | **ps** | **pn** | **dS** | **dN** | **ds/dn** | **ps/pn** |
| --- | --- | --- | --- | --- | --- | --- | --- | --- | --- | --- | --- |
| 0 1 | TEA006436.1/ TEA007103.1 | 1489.333 | 5612.667 | 2044.333 | 7630.667 | 0.7285 | 0.7355 | 2.6646 | 2.9616 | 0.8997 | 0.9905 |
| 0 2 | TEA006436.1/ TEA012905.1 | 1185.167 | 4731.833 | 1677.833 | 6614.167 | 0.7064 | 0.7154 | 2.1332 | 2.3074 | 0.9245 | 0.9874 |
| 0 3 | TEA006436.1/ TEA015676.1 | 1036.167 | 3997.833 | 1423 | 5471 | 0.7282 | 0.7307 | 2.6521 | 2.7462 | 0.9657 | 0.9965 |
| 0 4 | TEA006436.1/ TEA016315.1 | 469.6667 | 1788.333 | 646.6667 | 2473.333 | 0.7263 | 0.723 | 2.5906 | 2.4945 | 1.0385 | 1.0045 |
| 0 5 | TEA006436.1/ TEA018880.1 | 818.3333 | 3288.667 | 1173.667 | 4523.333 | 0.6972 | 0.727 | 1.9908 | 2.6149 | 0.7613 | 0.959 |
| 0 6 | TEA006436.1/ TEA020852.1 | 1070.5 | 4329.5 | 1504.667 | 5923.333 | 0.7115 | 0.7309 | 2.2262 | 2.7537 | 0.8084 | 0.9734 |
| 0 7 | TEA006436.1/ TEA021759.1 | 1043 | 4216 | 1495.5 | 5881.5 | 0.6974 | 0.7168 | 1.9934 | 2.3387 | 0.8524 | 0.9729 |
| 0 8 | TEA006436.1/ TEA022253.1 | 1841.667 | 7166.333 | 2572.5 | 9817.5 | 0.7159 | 0.73 | 2.3182 | 2.7166 | 0.8534 | 0.9808 |
| 0 9 | TEA006436.1/ TEA022268.1 | 1918.667 | 7094.333 | 2608.5 | 9781.5 | 0.7355 | 0.7253 | 2.9617 | 2.5594 | 1.1572 | 1.0142 |
| 0 10 | TEA006436.1/ TEA024415.1 | 1801 | 7191 | 2566.667 | 9823.333 | 0.7017 | 0.732 | 2.0568 | 2.7986 | 0.7349 | 0.9585 |
| 0 11 | TEA006436.1/ TEA026040.1 | 784.1667 | 3204.833 | 1114.5 | 4450.5 | 0.7036 | 0.7201 | 2.0871 | 2.4168 | 0.8636 | 0.9771 |
| 0 12 | TEA006436.1/ TEA026883.1 | 1841.167 | 7177.833 | 2561.167 | 9774.833 | 0.7189 | 0.7343 | 2.3866 | 2.9007 | 0.8228 | 0.979 |
| 0 13 | TEA006436.1/ TEA031053.1 | 966.3333 | 3723.667 | 1344 | 5172 | 0.719 | 0.72 | 2.3895 | 2.41033 | 0.9901 | 0.9987 |
| 0 14 | TEA006436.1/ TEA031435.1 | 564.1667 | 2255.833 | 800.3333 | 3042.667 | 0.7049 | 0.7414 | 2.1086 | 3.3512 | 0.6292 | 0.9508 |
| 0 15 | TEA006436.1/ TEA032724.1 | 573.5 | 2278.5 | 818.1667 | 3126.833 | 0.701 | 0.7287 | 2.0455 | 2.6708 | 0.7659 | 0.9619 |
| 1 2 | TEA007103.1/ TEA012905.1 | 880.6667 | 3282.333 | 1186.333 | 4390.667 | 0.7423 | 0.7476 | 3.4384 | 4.2993 | 0.7997 | 0.993 |
| 1 3 | TEA007103.1/ TEA015676.1 | 866 | 3195 | 1181.333 | 4287.667 | 0.7331 | 0.7452 | 2.8432 | 3.7825 | 0.7517 | 0.9838 |
| 1 4 | TEA007103.1/ TEA016315.1 | 473.8333 | 1829.167 | 656.3333 | 2463.667 | 0.7219 | 0.7425 | 2.4643 | 3.4496 | 0.7144 | 0.9724 |
| 1 5 | TEA007103.1/ TEA018880.1 | 875.6667 | 3137.333 | 1174.667 | 4294.333 | 0.7455 | 0.7306 | 3.8303 | 2.7401 | 1.3979 | 1.0204 |
| 1 6 | TEA007103.1/ TEA020852.1 | 851.6667 | 3179.333 | 1153.167 | 4315.833 | 0.7385 | 0.7367 | 3.1363 | 3.0224 | 1.0377 | 1.0026 |
| 1 7 | TEA007103.1/ TEA021759.1 | 849.8333 | 3172.167 | 1144.667 | 4324.333 | 0.7424 | 0.7336 | 3.4468 | 2.8654 | 1.2029 | 1.0121 |
| 1 8 | TEA007103.1/ TEA022253.1 | 1707.833 | 6290.167 | 2326.833 | 8623.167 | 0.734 | 0.7294 | 2.8844 | 2.6979 | 1.0691 | 1.0062 |
| 1 9 | TEA007103.1/ TEA022268.1 | 2377 | 8978 | 3287.833 | 12288.17 | 0.723 | 0.7306 | 2.4923 | 2.7419 | 0.909 | 0.9895 |
| 1 10 | TEA007103.1/ TEA024415.1 | 1625.333 | 6078.667 | 2249.667 | 8340.333 | 0.7225 | 0.7288 | 2.4788 | 2.6755 | 0.9265 | 0.9913 |
| 1 11 | TEA007103.1/ TEA026040.1 | 809 | 3201 | 1139.667 | 4329.333 | 0.7099 | 0.7394 | 2.1957 | 3.1926 | 0.6877 | 0.9601 |
| 1 12 | TEA007103.1/ TEA026883.1 | 2012.167 | 7642.833 | 2799 | 10524 | 0.7189 | 0.7262 | 2.3869 | 2.5887 | 0.922 | 0.9899 |
| 1 13 | TEA007103.1/ TEA031053.1 | 852.3333 | 3138.667 | 1169.833 | 4299.167 | 0.7286 | 0.7301 | 2.6673 | 2.7207 | 0.9804 | 0.998 |
| 1 14 | TEA007103.1/ TEA031435.1 | 588.6667 | 2231.333 | 819.8333 | 3023.167 | 0.718 | 0.7381 | 2.3665 | 3.1063 | 0.7618 | 0.9728 |
| 1 15 | TEA007103.1/ TEA032724.1 | 607.6667 | 2348.333 | 838 | 3107 | 0.7251 | 0.7558 | 2.5551 | nan | nan | 0.9594 |
| 2 3 | TEA012905.1/ TEA015676.1 | 1031.333 | 3964.667 | 1436 | 5458 | 0.7182 | 0.7264 | 2.3704 | 2.594 | 0.9138 | 0.9887 |
| 2 4 | TEA012905.1/ TEA016315.1 | 466.5 | 1783.5 | 651.3333 | 2468.667 | 0.7162 | 0.7225 | 2.3252 | 2.4782 | 0.9383 | 0.9914 |
| 2 5 | TEA012905.1/ TEA018880.1 | 860.5 | 3284.5 | 1188.333 | 4508.667 | 0.7241 | 0.7285 | 2.5251 | 2.6635 | 0.948 | 0.994 |
| 2 6 | TEA012905.1/ TEA020852.1 | 1082.333 | 4281.667 | 1516.167 | 5911.833 | 0.7139 | 0.7243 | 2.2745 | 2.5288 | 0.8994 | 0.9857 |
| 2 7 | TEA012905.1/ TEA021759.1 | 1110.833 | 4305.167 | 1506 | 5871 | 0.7376 | 0.7333 | 3.0771 | 2.8532 | 1.0785 | 1.0059 |
| 2 8 | TEA012905.1/ TEA022253.1 | 1171.5 | 4805.5 | 1714.833 | 6577.167 | 0.6832 | 0.7306 | 1.8133 | 2.7424 | 0.6612 | 0.935 |
| 2 9 | TEA012905.1/ TEA022268.1 | 1260.667 | 4747.333 | 1718.167 | 6573.833 | 0.7337 | 0.7222 | 2.873 | 2.4701 | 1.1631 | 1.016 |
| 2 10 | TEA012905.1/ TEA024415.1 | 1222 | 4830 | 1714.167 | 6577.833 | 0.7129 | 0.7343 | 2.2545 | 2.8991 | 0.7777 | 0.9709 |
| 2 11 | TEA012905.1/ TEA026040.1 | 817.3333 | 3200.667 | 1126.833 | 4438.167 | 0.7253 | 0.7212 | 2.5611 | 2.444 | 1.0479 | 1.0058 |
| 2 12 | TEA012905.1/ TEA026883.1 | 1230.333 | 4783.667 | 1716.333 | 6521.667 | 0.7168 | 0.7335 | 2.339 | 2.8627 | 0.8171 | 0.9773 |
| 2 13 | TEA012905.1/ TEA031053.1 | 999.5 | 3757.5 | 1357.167 | 5158.833 | 0.7365 | 0.7284 | 3.0109 | 2.6592 | 1.1322 | 1.0111 |
| 2 14 | TEA012905.1/ TEA031435.1 | 581.8333 | 2229.167 | 806.5 | 3036.5 | 0.7214 | 0.7341 | 2.4508 | 2.8914 | 0.8476 | 0.9827 |
| 2 15 | TEA012905.1/ TEA032724.1 | 615.6667 | 2266.333 | 823.5 | 3121.5 | 0.7476 | 0.726 | 4.3153 | 2.5828 | 1.6708 | 1.0297 |
| 3 4 | TEA015676.1/ TEA016315.1 | 484.8333 | 1800.167 | 656.6667 | 2463.333 | 0.7383 | 0.7308 | 3.122 | 2.7483 | 1.136 | 1.0103 |
| 3 5 | TEA015676.1/ TEA018880.1 | 873.5 | 3199.5 | 1207.167 | 4489.833 | 0.7236 | 0.7126 | 2.5099 | 2.249 | 1.116 | 1.0154 |
| 3 6 | TEA015676.1/ TEA020852.1 | 1004.5 | 3930.5 | 1426.833 | 5467.167 | 0.704 | 0.7189 | 2.0937 | 2.3878 | 0.8768 | 0.9792 |
| 3 7 | TEA015676.1/ TEA021759.1 | 1008 | 3975 | 1427.667 | 5466.333 | 0.706 | 0.7272 | 2.1277 | 2.6193 | 0.8123 | 0.9709 |
| 3 8 | TEA015676.1/ TEA022253.1 | 1068.167 | 3914.833 | 1453.833 | 5440.167 | 0.7347 | 0.7196 | 2.9204 | 2.4046 | 1.2145 | 1.021 |
| 3 9 | TEA015676.1/ TEA022268.1 | 1028.5 | 3936.5 | 1440.833 | 5453.167 | 0.7138 | 0.7219 | 2.2737 | 2.4625 | 0.9233 | 0.9888 |
| 3 10 | TEA015676.1/ TEA024415.1 | 1053.333 | 3995.667 | 1446.5 | 5447.5 | 0.7282 | 0.7335 | 2.6534 | 2.8619 | 0.9272 | 0.9928 |
| 3 11 | TEA015676.1/ TEA026040.1 | 782.5 | 3229.5 | 1145.333 | 4419.667 | 0.6832 | 0.7307 | 1.8139 | 2.7454 | 0.6607 | 0.935 |
| 3 12 | TEA015676.1/ TEA026883.1 | 1057.667 | 3940.333 | 1463.833 | 5376.167 | 0.7225 | 0.7329 | 2.4803 | 2.8369 | 0.8743 | 0.9858 |
| 3 13 | TEA015676.1/ TEA031053.1 | 994 | 3763 | 1380.167 | 5135.833 | 0.7202 | 0.7327 | 2.4192 | 2.8268 | 0.8558 | 0.983 |
| 3 14 | TEA015676.1/ TEA031435.1 | 584.5 | 2228.5 | 815.6667 | 3027.333 | 0.7166 | 0.7361 | 2.3335 | 2.9926 | 0.7798 | 0.9735 |
| 3 15 | TEA015676.1/ TEA032724.1 | 595.5 | 2272.5 | 833.1667 | 3111.833 | 0.7147 | 0.7303 | 2.2931 | 2.7287 | 0.8403 | 0.9787 |
| 4 5 | TEA016315.1/ TEA018880.1 | 463.1667 | 1790.833 | 653.3333 | 2466.667 | 0.7089 | 0.726 | 2.1786 | 2.5819 | 0.8438 | 0.9765 |
| 4 6 | TEA016315.1/ TEA020852.1 | 460.3333 | 1826.667 | 643.3333 | 2476.667 | 0.7155 | 0.7376 | 2.3103 | 3.0738 | 0.7516 | 0.9702 |
| 4 7 | TEA016315.1/ TEA021759.1 | 448.6667 | 1790.333 | 637.5 | 2482.5 | 0.7038 | 0.7212 | 2.0902 | 2.4443 | 0.8551 | 0.9759 |
| 4 8 | TEA016315.1/ TEA022253.1 | 464.6667 | 1789.333 | 651.6667 | 2468.333 | 0.713 | 0.7249 | 2.2577 | 2.5484 | 0.886 | 0.9836 |
| 4 9 | TEA016315.1/ TEA022268.1 | 458.5 | 1862.5 | 651 | 2469 | 0.7043 | 0.7544 | 2.0985 | nan | nan | 0.9336 |
| 4 10 | TEA016315.1/ TEA024415.1 | 485.6667 | 1843.333 | 663.8333 | 2456.167 | 0.7316 | 0.7505 | 2.7812 | nan | nan | 0.9748 |
| 4 11 | TEA016315.1/ TEA026040.1 | 435.5 | 1784.5 | 632.6667 | 2487.333 | 0.6884 | 0.7174 | 1.874 | 2.3526 | 0.7966 | 0.9595 |
| 4 12 | TEA016315.1/ TEA026883.1 | 467.3333 | 1770.667 | 656 | 2464 | 0.7124 | 0.7186 | 2.2448 | 2.3803 | 0.9431 | 0.9913 |
| 4 13 | TEA016315.1/ TEA031053.1 | 479.6667 | 1816.333 | 652.6667 | 2467.333 | 0.7349 | 0.7362 | 2.9307 | 2.994 | 0.9789 | 0.9983 |
| 4 14 | TEA016315.1/ TEA031435.1 | 471.5 | 1762.5 | 651.8333 | 2468.167 | 0.7233 | 0.7141 | 2.5028 | 2.2794 | 1.098 | 1.013 |
| 4 15 | TEA016315.1/ TEA032724.1 | 472.1667 | 1833.833 | 648.3333 | 2471.667 | 0.7283 | 0.7419 | 2.6563 | 3.4001 | 0.7813 | 0.9816 |
| 5 6 | TEA018880.1/ TEA020852.1 | 881.6667 | 3304.333 | 1179.333 | 4517.667 | 0.7476 | 0.7314 | 4.3077 | 2.7737 | 1.5531 | 1.0221 |
| 5 7 | TEA018880.1/ TEA021759.1 | 808.6667 | 3315.333 | 1172.5 | 4524.5 | 0.6897 | 0.7328 | 1.8905 | 2.8293 | 0.6682 | 0.9412 |
| 5 8 | TEA018880.1/ TEA022253.1 | 842.5 | 3286.5 | 1198.167 | 4498.833 | 0.7032 | 0.7305 | 2.08 | 2.7381 | 0.7596 | 0.9625 |
| 5 9 | TEA018880.1/ TEA022268.1 | 862.1667 | 3290.833 | 1186.833 | 4510.167 | 0.7264 | 0.7296 | 2.5955 | 2.7052 | 0.9595 | 0.9956 |
| 5 10 | TEA018880.1/ TEA024415.1 | 855.1667 | 3352.833 | 1195 | 4502 | 0.7156 | 0.7447 | 2.312 | 3.7204 | 0.6214 | 0.9609 |
| 5 11 | TEA018880.1/ TEA026040.1 | 819.5 | 3170.5 | 1136.833 | 4428.167 | 0.7209 | 0.716 | 2.436 | 2.3199 | 1.05 | 1.0068 |
| 5 12 | TEA018880.1/ TEA026883.1 | 857.6667 | 3219.333 | 1198.667 | 4444.333 | 0.7155 | 0.7244 | 2.3097 | 2.5322 | 0.9121 | 0.9878 |
| 5 13 | TEA018880.1/ TEA031053.1 | 845.3333 | 3281.667 | 1198 | 4499 | 0.7056 | 0.7294 | 2.1205 | 2.6969 | 0.7863 | 0.9674 |
| 5 14 | TEA018880.1/ TEA031435.1 | 600 | 2198 | 810.8333 | 3032.167 | 0.74 | 0.7249 | 3.2366 | 2.5477 | 1.2704 | 1.0208 |
| 5 15 | TEA018880.1/ TEA032724.1 | 598.1667 | 2236.833 | 827.5 | 3117.5 | 0.7229 | 0.7175 | 2.4893 | 2.3543 | 1.0573 | 1.0075 |
| 6 7 | TEA020852.1/ TEA021759.1 | 1080.5 | 4346.5 | 1502.5 | 5874.5 | 0.7191 | 0.7399 | 2.3928 | 3.2301 | 0.7408 | 0.9719 |
| 6 8 | TEA020852.1/ TEA022253.1 | 1082.333 | 4303.667 | 1538.833 | 5889.167 | 0.7033 | 0.7308 | 2.083 | 2.748 | 0.758 | 0.9625 |
| 6 9 | TEA020852.1/ TEA022268.1 | 1088.833 | 4268.167 | 1531.833 | 5896.167 | 0.7108 | 0.7239 | 2.2136 | 2.5183 | 0.879 | 0.9819 |
| 6 10 | TEA020852.1/ TEA024415.1 | 1089.333 | 4312.667 | 1535.5 | 5892.5 | 0.7094 | 0.7319 | 2.1878 | 2.7927 | 0.7834 | 0.9693 |
| 6 11 | TEA020852.1/ TEA026040.1 | 798.8333 | 3149.167 | 1115.167 | 4449.833 | 0.7163 | 0.7077 | 2.3277 | 2.1565 | 1.0794 | 1.0122 |
| 6 12 | TEA020852.1/ TEA026883.1 | 1129.5 | 4233.5 | 1546.333 | 5827.667 | 0.7304 | 0.7264 | 2.7348 | 2.5957 | 1.0536 | 1.0055 |
| 6 13 | TEA020852.1/ TEA031053.1 | 983.1667 | 3773.833 | 1350 | 5166 | 0.7283 | 0.7305 | 2.6561 | 2.7378 | 0.9702 | 0.9969 |
| 6 14 | TEA020852.1/ TEA031435.1 | 598.1667 | 2241.833 | 793.5 | 3049.5 | 0.7538 | 0.7351 | nan | 2.9414 | nan | 1.0254 |
| 6 15 | TEA020852.1/ TEA032724.1 | 581.8333 | 2255.167 | 809.8333 | 3135.167 | 0.7185 | 0.7193 | 2.3766 | 2.3972 | 0.9914 | 0.9988 |
| 7 8 | TEA021759.1/ TEA022253.1 | 1101.5 | 4265.5 | 1529 | 5848 | 0.7204 | 0.7294 | 2.4244 | 2.6959 | 0.8993 | 0.9877 |
| 7 9 | TEA021759.1/ TEA022268.1 | 1106 | 4194 | 1521 | 5856 | 0.7272 | 0.7162 | 2.6184 | 2.3245 | 1.1265 | 1.0153 |
| 7 10 | TEA021759.1/ TEA024415.1 | 1095.333 | 4234.667 | 1526.167 | 5850.833 | 0.7177 | 0.7238 | 2.3588 | 2.5149 | 0.9379 | 0.9916 |
| 7 11 | TEA021759.1/ TEA026040.1 | 791 | 3271 | 1108.667 | 4456.333 | 0.7135 | 0.734 | 2.2664 | 2.8862 | 0.7853 | 0.972 |
| 7 12 | TEA021759.1/ TEA026883.1 | 1108.667 | 4240.333 | 1536.333 | 5786.667 | 0.7216 | 0.7328 | 2.4561 | 2.8303 | 0.8678 | 0.9848 |
| 7 13 | TEA021759.1/ TEA031053.1 | 980.6667 | 3752.333 | 1348.333 | 5167.667 | 0.7273 | 0.7261 | 2.6239 | 2.5852 | 1.015 | 1.0017 |
| 7 14 | TEA021759.1/ TEA031435.1 | 584.8333 | 2233.167 | 788.6667 | 3054.333 | 0.7415 | 0.7311 | 3.3642 | 2.7626 | 1.2178 | 1.0142 |
| 7 15 | TEA021759.1/ TEA032724.1 | 590.1667 | 2228.833 | 804.6667 | 3140.333 | 0.7334 | 0.7097 | 2.8594 | 2.1936 | 1.3035 | 1.0334 |
| 8 9 | TEA022253.1/ TEA022268.1 | 2144.667 | 7856.333 | 2927.5 | 10737.5 | 0.7326 | 0.7317 | 2.8224 | 2.7838 | 1.0139 | 1.0013 |
| 8 10 | TEA022253.1/ TEA024415.1 | 2016.167 | 7717.833 | 2803.5 | 10501.5 | 0.7192 | 0.7349 | 2.3935 | 2.9304 | 0.8168 | 0.9785 |
| 8 11 | TEA022253.1/ TEA026040.1 | 807.1667 | 3216.833 | 1135.667 | 4429.333 | 0.7107 | 0.7263 | 2.2124 | 2.5896 | 0.8544 | 0.9786 |
| 8 12 | TEA022253.1/ TEA026883.1 | 2099.5 | 7949.5 | 2869 | 10742 | 0.7318 | 0.74 | 2.7885 | 3.2411 | 0.8604 | 0.9889 |
| 8 13 | TEA022253.1/ TEA031053.1 | 1005.333 | 3793.667 | 1374 | 5142 | 0.7317 | 0.7378 | 2.7842 | 3.0878 | 0.9017 | 0.9917 |
| 8 14 | TEA022253.1/ TEA031435.1 | 586.8333 | 2195.167 | 806.8333 | 3036.167 | 0.7273 | 0.723 | 2.6242 | 2.4933 | 1.0525 | 1.006 |
| 8 15 | TEA022253.1/ TEA032724.1 | 587 | 2244 | 822.3333 | 3122.667 | 0.7138 | 0.7186 | 2.2737 | 2.3803 | 0.9552 | 0.9933 |
| 9 10 | TEA022268.1/ TEA024415.1 | 2037.333 | 7665.667 | 2840.333 | 10464.67 | 0.7173 | 0.7325 | 2.3492 | 2.8196 | 0.8332 | 0.9792 |
| 9 11 | TEA022268.1/ TEA026040.1 | 818 | 3188 | 1126.167 | 4438.833 | 0.7264 | 0.7182 | 2.5928 | 2.3706 | 1.0937 | 1.0113 |
| 9 12 | TEA022268.1/ TEA026883.1 | 2500 | 9219 | 3413.667 | 12624.33 | 0.7324 | 0.7303 | 2.812 | 2.7279 | 1.0308 | 1.0029 |
| 9 13 | TEA022268.1/ TEA031053.1 | 962 | 3743 | 1359.5 | 5156.5 | 0.7076 | 0.7259 | 2.1549 | 2.5778 | 0.836 | 0.9748 |
| 9 14 | TEA022268.1/ TEA031435.1 | 611.6667 | 2237.333 | 802.1667 | 3040.833 | 0.7625 | 0.7358 | nan | 2.9732 | nan | 1.0364 |
| 9 15 | TEA022268.1/ TEA032724.1 | 566.3333 | 2297.667 | 820.5 | 3124.5 | 0.6902 | 0.7354 | 1.8972 | 2.9528 | 0.6425 | 0.9386 |
| 10 11 | TEA024415.1/ TEA026040.1 | 799 | 3207 | 1134.5 | 4430.5 | 0.7043 | 0.7238 | 2.0981 | 2.5171 | 0.8335 | 0.973 |
| 10 12 | TEA024415.1/ TEA026883.1 | 2047.833 | 7542.167 | 2783 | 10468 | 0.7358 | 0.7205 | 2.9771 | 2.4267 | 1.2268 | 1.0213 |
| 10 13 | TEA024415.1/ TEA031053.1 | 982.1667 | 3755.833 | 1367.667 | 5148.333 | 0.7181 | 0.7295 | 2.3689 | 2.7006 | 0.8772 | 0.9844 |
| 10 14 | TEA024415.1/ TEA031435.1 | 618.8333 | 2244.167 | 822.1667 | 3020.833 | 0.7527 | 0.7429 | nan | 3.4946 | nan | 1.0132 |
| 10 15 | TEA024415.1/ TEA032724.1 | 618.1667 | 2262.833 | 835.8333 | 3109.167 | 0.7396 | 0.7278 | 3.2074 | 2.6398 | 1.215 | 1.0162 |
| 11 12 | TEA026040.1/ TEA026883.1 | 781.5 | 3166.5 | 1137.667 | 4373.333 | 0.6869 | 0.724 | 1.8569 | 2.5228 | 0.736 | 0.9487 |
| 11 13 | TEA026040.1/ TEA031053.1 | 805.6667 | 3264.333 | 1135 | 4430 | 0.7098 | 0.7369 | 2.1954 | 3.0339 | 0.7236 | 0.9633 |
| 11 14 | TEA026040.1/ TEA031435.1 | 546 | 2209 | 781.6667 | 3061.333 | 0.6985 | 0.7216 | 2.009 | 2.4548 | 0.8184 | 0.968 |
| 11 15 | TEA026040.1/ TEA032724.1 | 570.5 | 2273.5 | 798.1667 | 3146.833 | 0.7148 | 0.7225 | 2.2935 | 2.4787 | 0.9253 | 0.9893 |
| 12 13 | TEA026883.1/ TEA031053.1 | 967.8333 | 3698.167 | 1377.5 | 5084.5 | 0.7026 | 0.7273 | 2.0711 | 2.6246 | 0.7891 | 0.966 |
| 12 14 | TEA026883.1/ TEA031435.1 | 556.6667 | 2260.333 | 812.6667 | 3030.333 | 0.685 | 0.7459 | 1.8341 | 3.9073 | 0.4694 | 0.9183 |
| 12 15 | TEA026883.1/ TEA032724.1 | 615.8333 | 2286.167 | 831.6667 | 3113.333 | 0.7405 | 0.7343 | 3.2751 | 2.9005 | 1.1291 | 1.0084 |
| 13 14 | TEA031053.1/ TEA031435.1 | 595.1667 | 2246.833 | 808.1667 | 3034.833 | 0.7364 | 0.7403 | 3.0097 | 3.2647 | 0.9219 | 0.9947 |
| 13 15 | TEA031053.1/ TEA032724.1 | 584.6667 | 2272.333 | 824.5 | 3120.5 | 0.7091 | 0.7282 | 2.182 | 2.6535 | 0.8223 | 0.9738 |
| 14 15 | TEA031435.1/ TEA032724.1 | 595.6667 | 2218.333 | 807 | 3036 | 0.7381 | 0.7307 | 3.1092 | 2.7441 | 1.1331 | 1.0102 |
