# Supplementary material for: MAPK cascade gene family in Camellia sinensis: In-silico identification, expression profiles and regulatory network analysis
Source: BMC Genomics. 2020 Sep 7;21:613. doi: 10.1186/s12864-020-07030-x (PMC7487466; doi:10.1186/s12864-020-07030-x)
Supplement: Supplementary file 2 — Additional file 2: Figure S1. Transmembrane helices data for the 5 MKK proteins in Camellia sinensis. TMHMM Server, v.2.0 (http://www.cbs.dtu.dk/services/TMHMM/), was used to predict the presence of transmembrane helices. Figure S2. Transmembrane helices data for the 16 MPK genes in Camellia sinensis. TMHMM Server, v.2.0 (http://www.cbs.dtu.dk/services/TMHMM/), was used to predict the presence of transmembrane helices. Figure S3. Phylogenetic analysis of MKK (A) and MPK (B) proteins by the minimum evolution (ME) method. The analysis was carried out among C. sinensis (black square), A. thaliana (black circle), O. sativa (black triangle), S. lycopersicum (black rhombus), S. tuberosum (red circle), C. annum (blue triangle), and C. canephora (grey triangle). The full-length MKK and MPK protein sequences were aligned using MUSCLE tool, and the phylogenetic tree was constructed using MEGA 7.0.14 by the minimum evolution (ME) method with default parameters and 1000 bootstrap replicates. Figure S4. Phylogenetic analysis of MKK (A) and MPK (B) proteins by the maximum likelihood (ML) method. The analysis was carried out among C. sinensis (black square), A. thaliana (black circle), O. sativa (black triangle), S. lycopersicum (black rhombus), S. tuberosum (red circle), C. annum (blue triangle), and C. canephora (grey triangle). The full-length MKK and MPK protein sequences were aligned using MUSCLE tool, and the phylogenetic tree was constructed using MEGA 7.0.14 by the maximum likelihood (ML) method with default parameters and 1000 bootstrap replicates. Figure S5. The motif logos of for MKK protein sequences. The motif logos were generated by MEME suite. The motif logo represents the conserved amino acid residues in the protein sequences. Figure S6. The motif logos of for MPK protein sequences. The motif logos were generated by MEME suite. The motif logo represents the conserved amino acid residues in the protein sequences. [file 12864_2020_7030_MOESM2_ESM.docx]

**Additional file 2**

**Supplementary Figure S1:**

**Transmembrane helices data for the 5 MKK proteins in *Camellia sinensis.*** TMHMM Server, v.2.0 (http://www.cbs.dtu.dk/services/TMHMM/), was used to predict the presence of transmembrane helices.


**Supplementary Figure S2:**

**Transmembrane helices data for the 16 MPK genes in *Camellia sinensis.*** TMHMM Server, v.2.0 (http://www.cbs.dtu.dk/services/TMHMM/), was used to predict the presence of transmembrane helices.

**Supplementary Figure S3:**

**Phylogenetic analysis of MKK (A) and MPK (B) proteins by the minimum evolution (ME) method.** The analysis was carried out among *C. sinensis* (black square), *A. thaliana* (black circle), *O. sativa* (black triangle), *S. lycopersicum* (black rhombus), *S. tuberosum* (red circle), *C. annum* (blue triangle), and *C. canephora* (grey triangle). The full-length MKK and MPK protein sequences were aligned using MUSCLE tool, and the phylogenetic tree was constructed using MEGA 7.0.14 by the minimum evolution (ME) method with default parameters and 1000 bootstrap replicates.

**Supplementary Figure S4:**

**Phylogenetic analysis of MKK (A) and MPK (B) proteins by the maximum likelihood (ML) method.** The analysis was carried out among C*. sinensis* (black square), *A. thaliana* (black circle), *O. sativa* (black triangle), *S. lycopersicum* (black rhombus), *S. tuberosum* (red circle), *C. annum* (blue triangle), and *C. canephora* (grey triangle). The full-length MKK and MPK protein sequences were aligned using MUSCLE tool, and the phylogenetic tree was constructed using MEGA 7.0.14 by the maximum likelihood (ML) method with default parameters and 1000 bootstrap replicates.

**Supplementary Figure S5:**

**The motif logos of for MKK protein sequences.** The motif logos were generated by MEME suite. The motif logo represents the conserved amino acid residues in the protein sequences.

**Supplementary Figure S6:**

**The motif logos of for MPK protein sequences.** The motif logos were generated by MEME suite. The motif logo represents the conserved amino acid residues in the protein sequences.
