# Supplementary material for: MAPK cascade gene family in Camellia sinensis: In-silico identification, expression profiles and regulatory network analysis
Source: BMC Genomics. 2020 Sep 7;21:613. doi: 10.1186/s12864-020-07030-x (PMC7487466; doi:10.1186/s12864-020-07030-x)
Supplement: Supplementary file 3 — Additional file 3: Figure S7: Alignment of domains in MPKs. All the MPK protein sequences were subjected to alignment by MUSCLE tool owing to their sequence diversities. Sequences that are highlighted are ATP binding signature, marked in blue, the catalytic C loop, marked in light red colour, the activation T loop, marked in green colour, CD domain, marked in light blue colour. Clades D to J show sequence derivations from the T(E/D) Y activation loop and are marked in a lighter shade of green colour. [file 12864_2020_7030_MOESM3_ESM.docx]

# Additional MPK domains

**Clade A**

IGxGxYGxV IKKIxxxF DAxRxLRE FxDIYxxxELM DLxxVI QxLRx


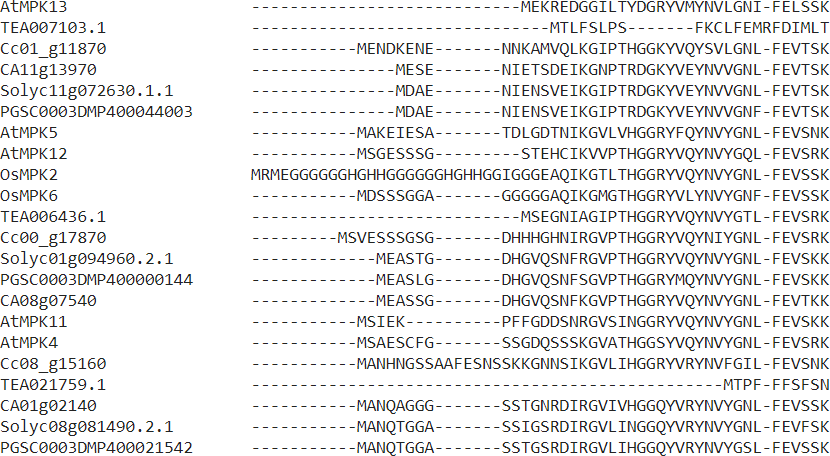

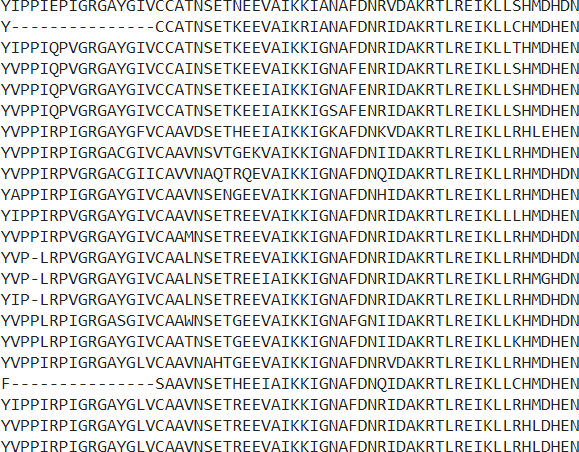

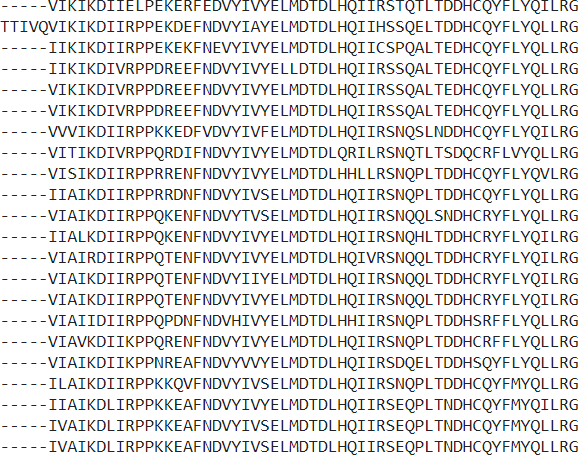


**LKYxH HRDLKPxN LxNxNCxLKIxDFGLAR TRWYRAPEL IDxWS^V^IGC KMLTFDP^K^RQKRITVE^D^EAL**


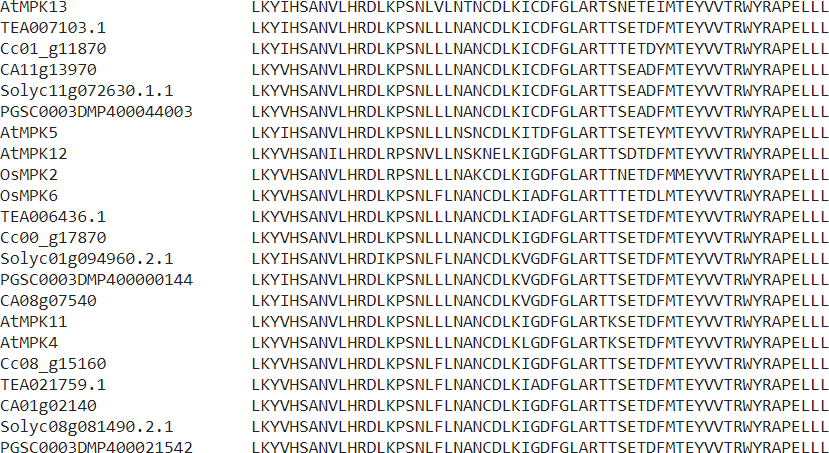

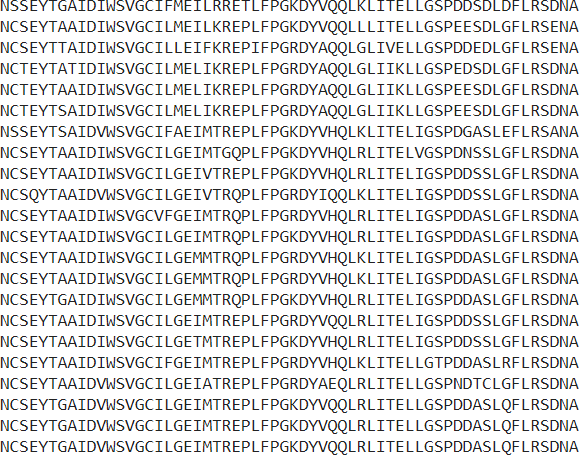

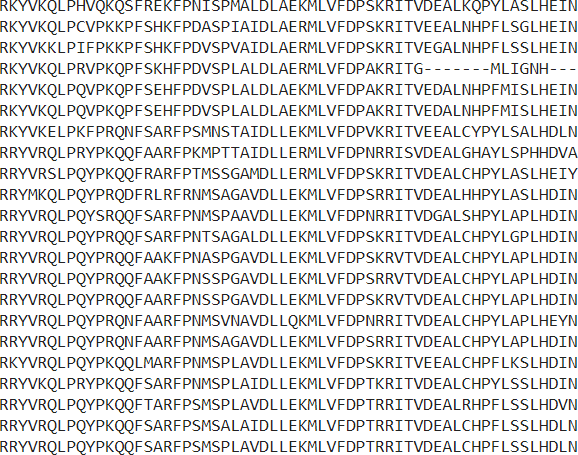

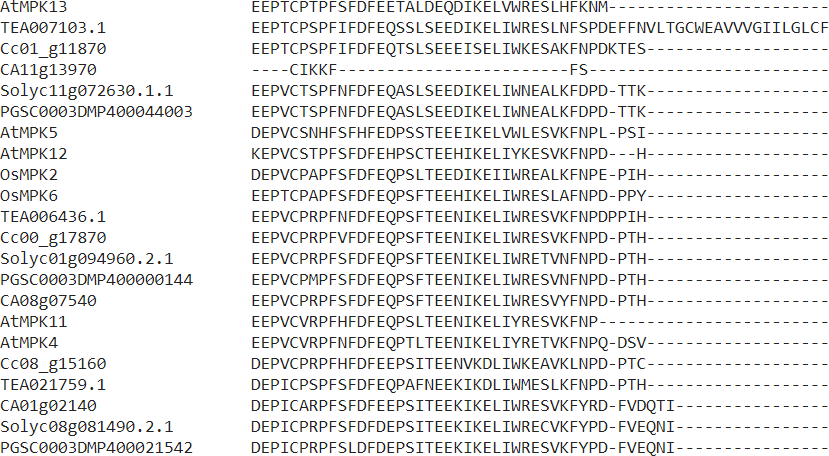

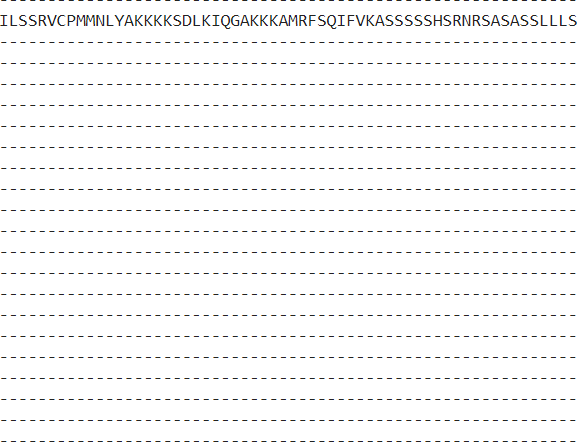

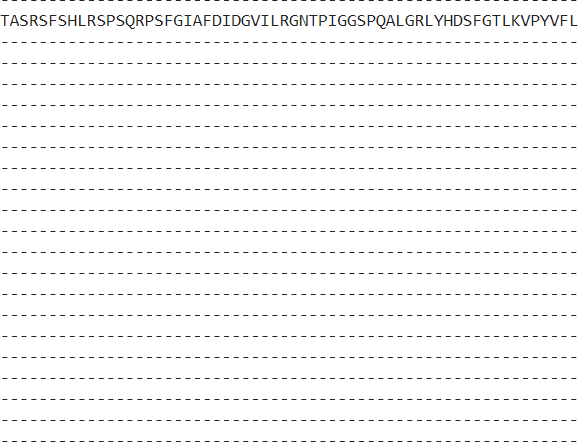

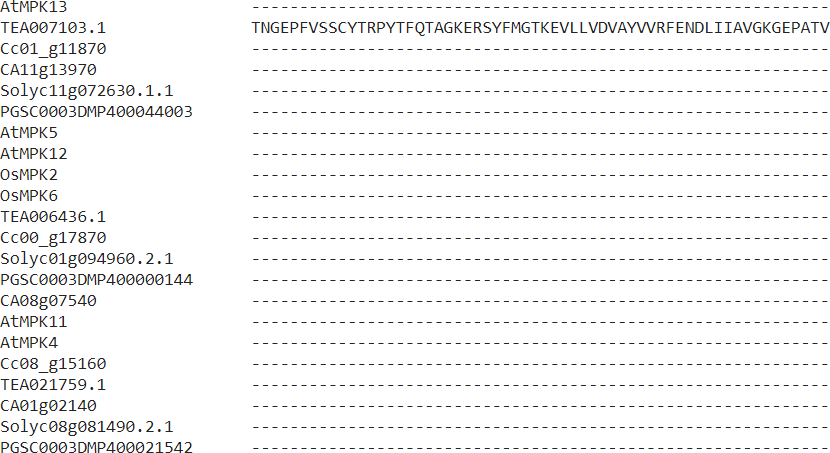

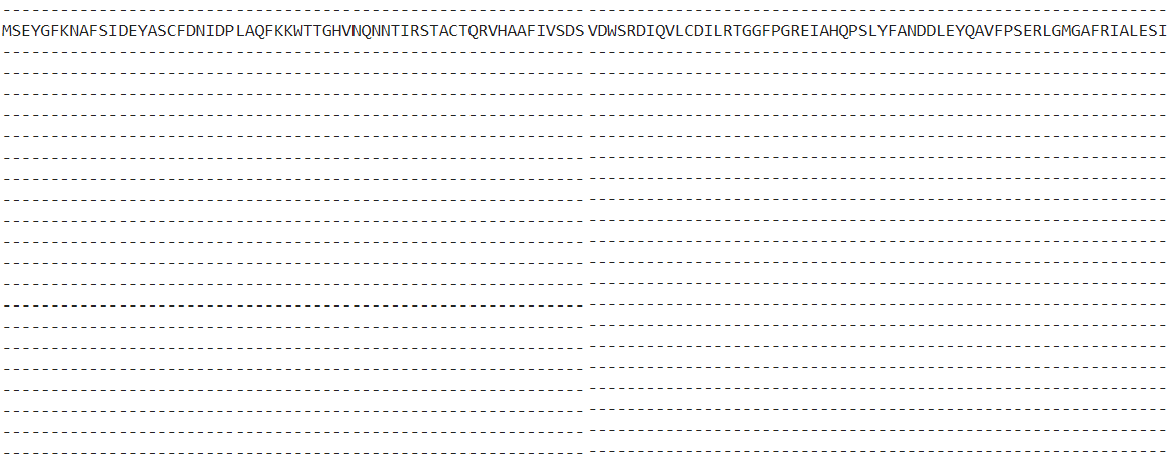


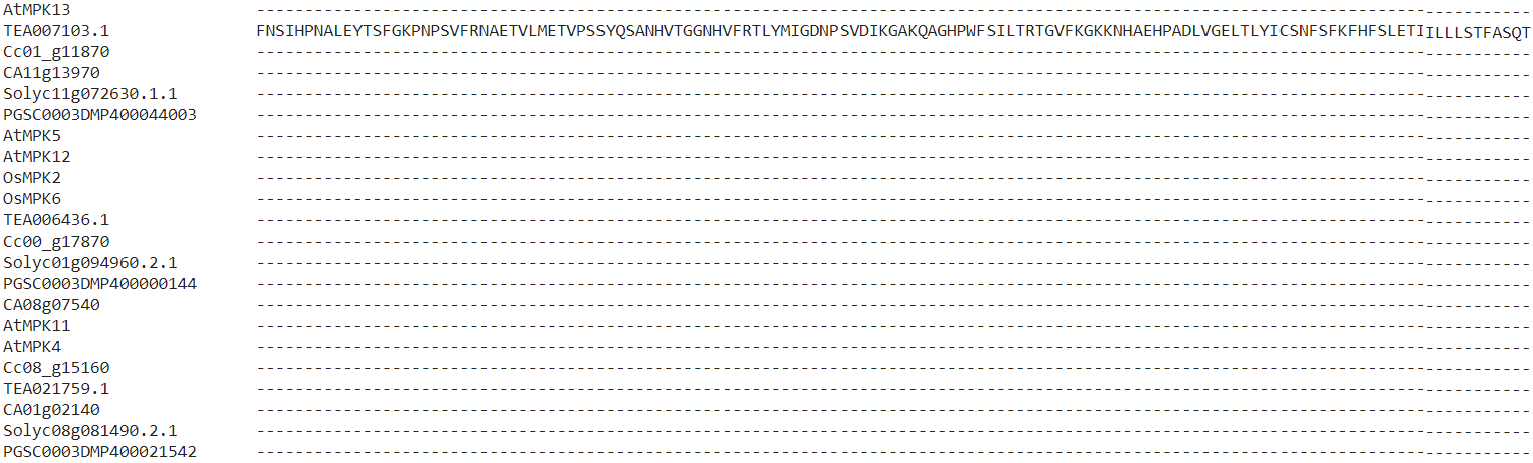


**Clade B IGxGxYGxV IKKIxxxF DAxRxLRE**


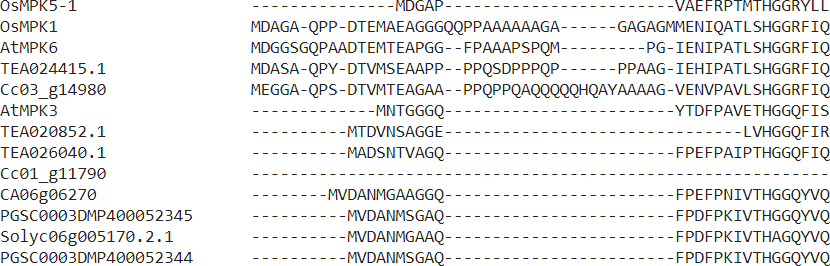

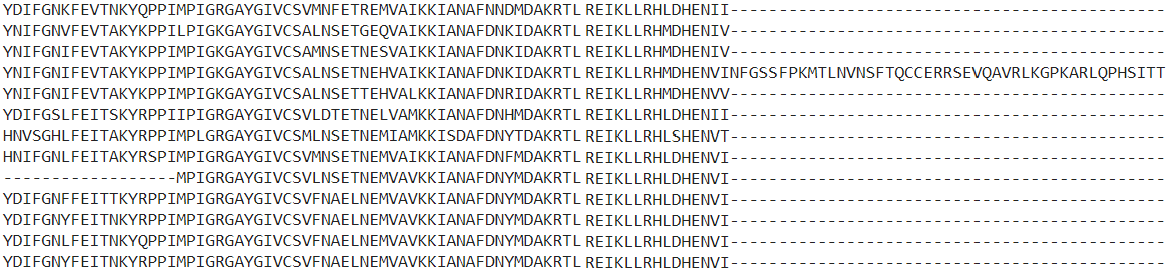


FxDIYxxxELM DLxxVI QxLRxLKYxH HRDLKPxN LxNxNCxLKIxDFGLAR TRWYRAPEL


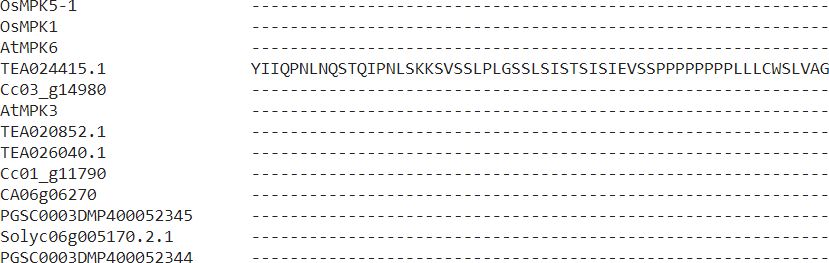

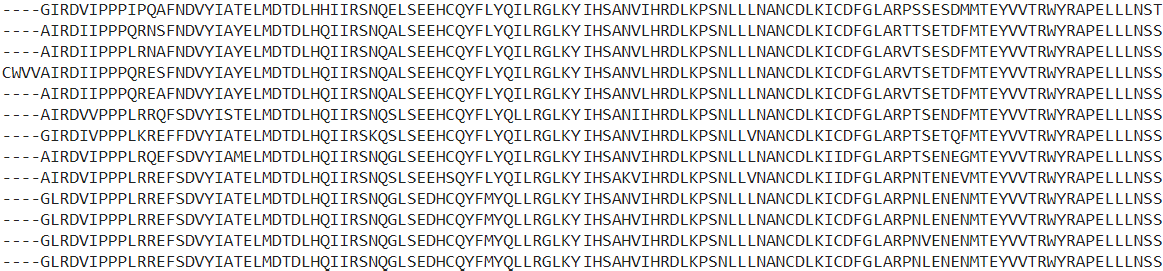


IDxWS^V^ GC

**I**

**KMLTFDP^K^ ^Q^ RITVE^D^ AL**

**R K E**


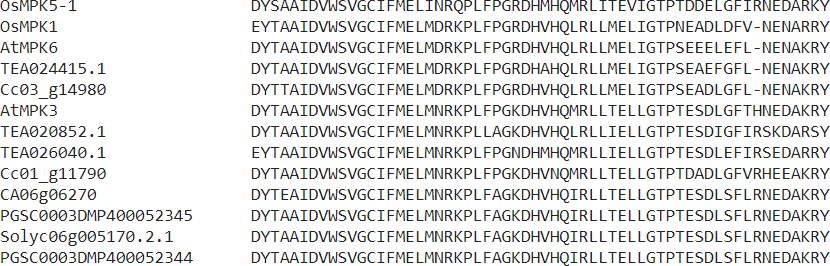

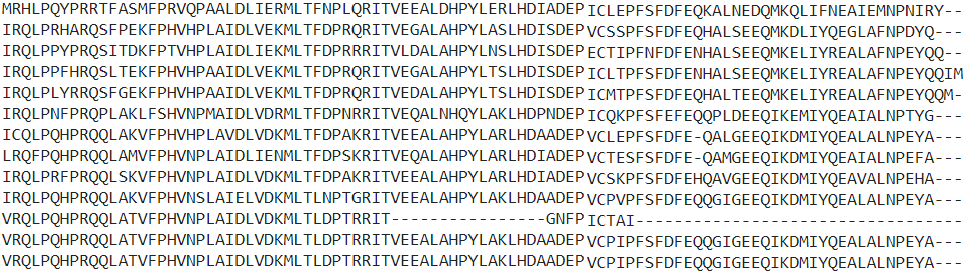


# Clade C

IGxGxYGxV IKKIxxxF DAxRxLRE FxDIYxxxELM DLxxVI


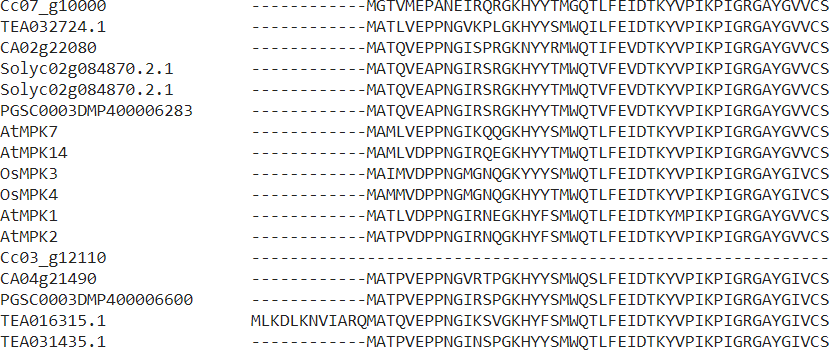

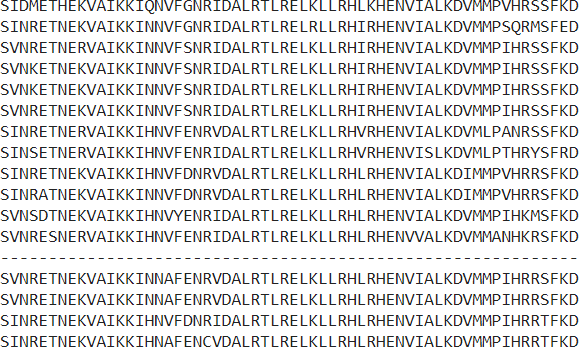

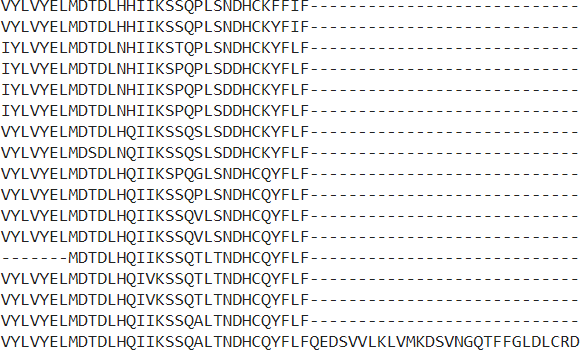


QxLRx LKYxH HRDLKPxN LxNxNCxLKIxDFGLAR TRWYRAPEL IDxWS^V^ GC K


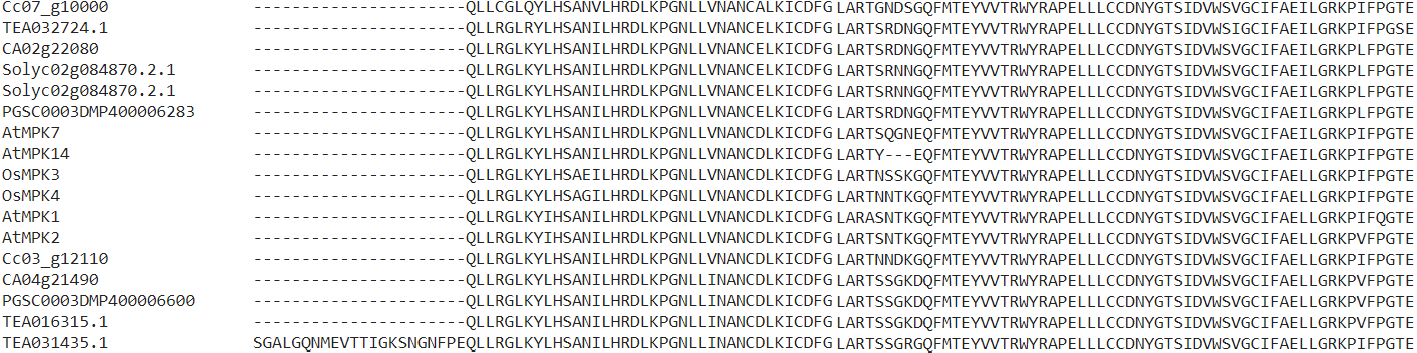

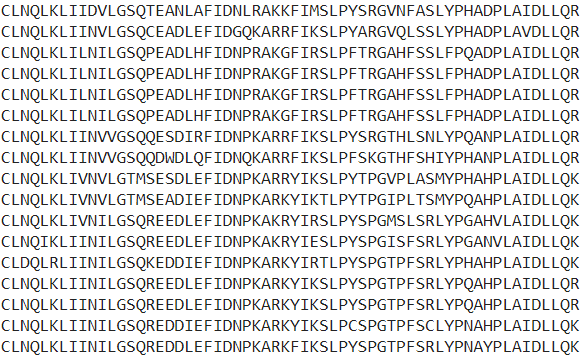


**I**

**MLTFDP^K^ ^Q^ RITVE^D^ AL**

**R K E**


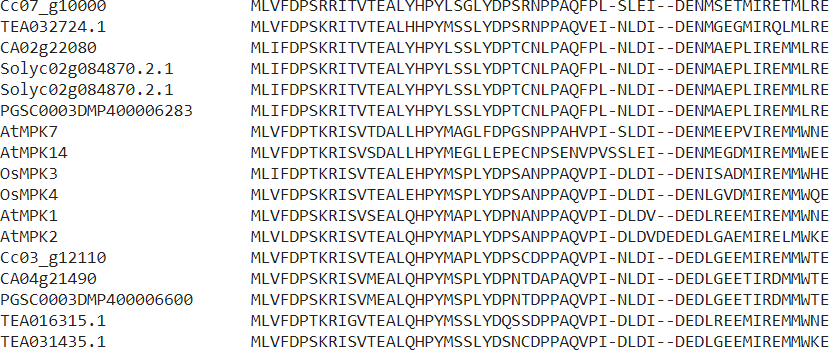

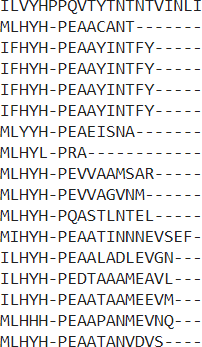


# Clade D

IGxGxYGxV IKKIxxxF DAxRxLRE FxDIYxxxELM DLxxVI QxLRxLKYxH HRDLKPxN LxNxNCxLKIxDFGLAR


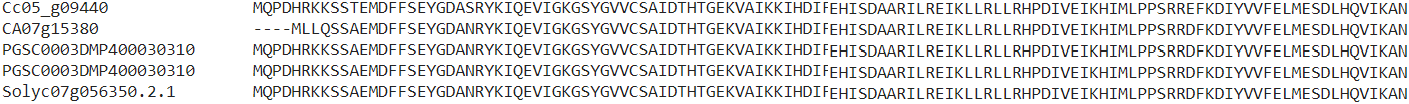

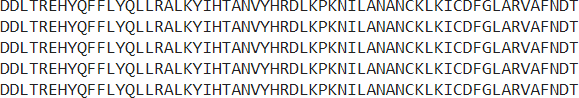


**TRWYRAPEL IDxWS^V^IGC LLE^R^KLLAFDPKDRPTAEEAL**


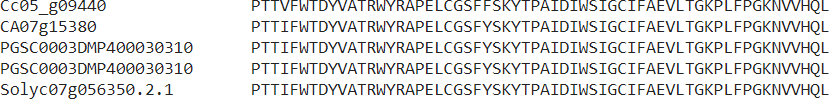

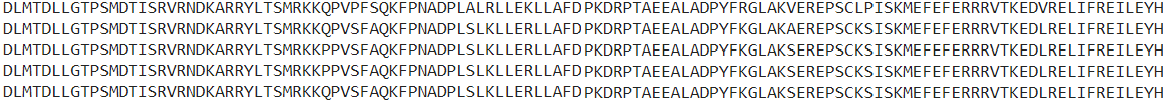

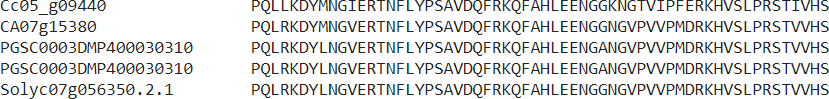

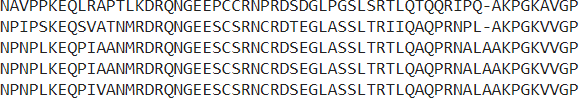

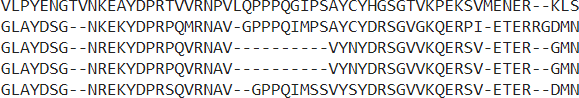

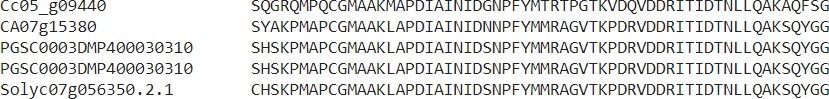

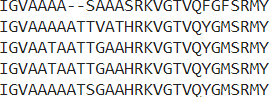


# Clade E


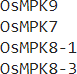


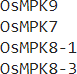


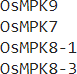


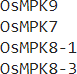


**Clade F**

IGxGxYGxV IKKIxxxF DAxRxLRE FxDIYxxxELM DLxxVI QxLRxLKYxH HRDLKPxN LxNxNCxLKIxD


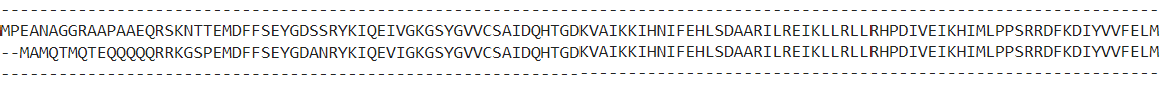

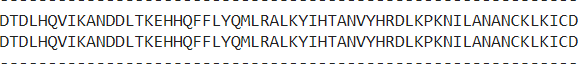


FGLAR TRWYRAPEL IDxWS IGC K


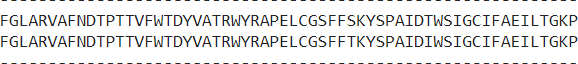

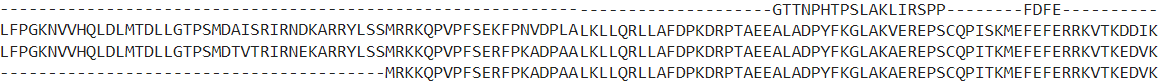


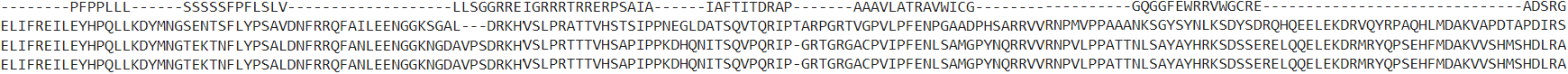


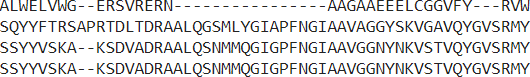


IGxGxYGxV IKKIxxxF DAxRxLRE FxDIYxxxELM DLxxVI QxLRxLKYxH HR

**V LLE^R^ LLAFDPKDRPTAEEAL**


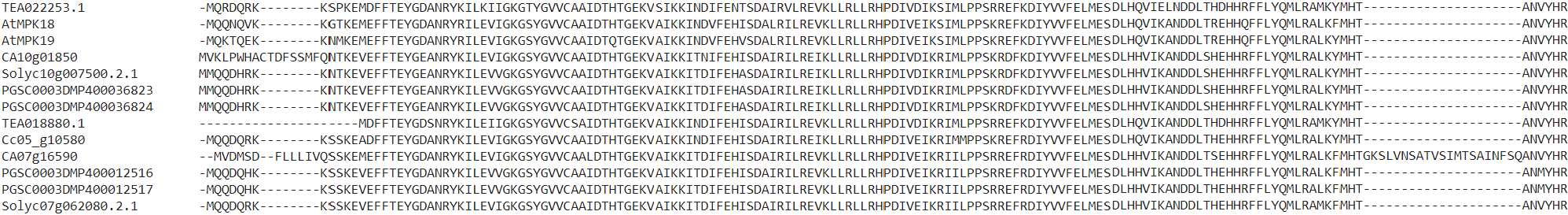


DLKPxN LxNxNCxLKIxDFGLAR TRWYRAPEL


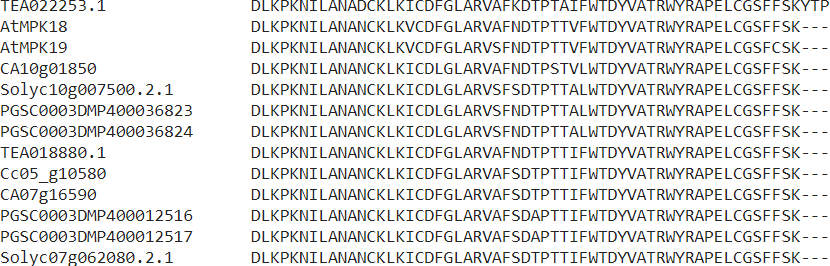

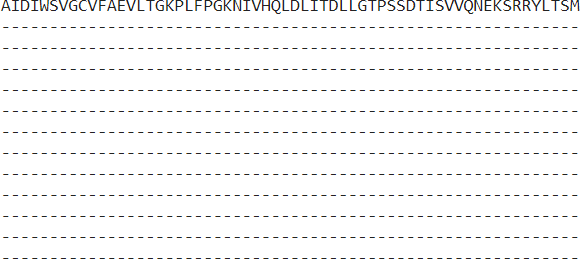

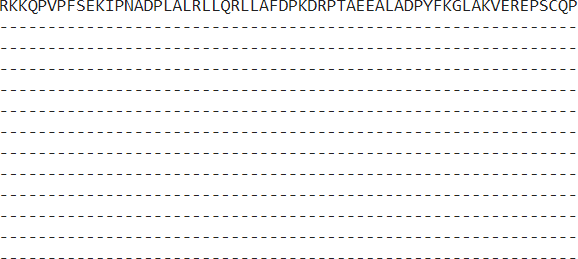


IDxWS^V^ GC

**I**


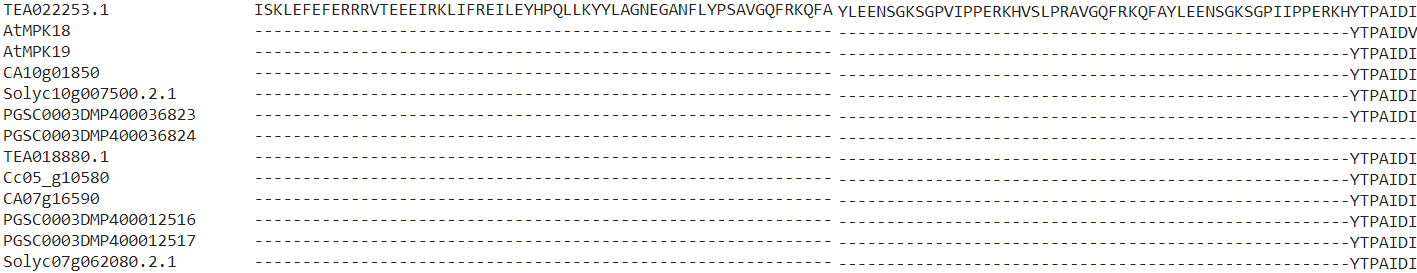

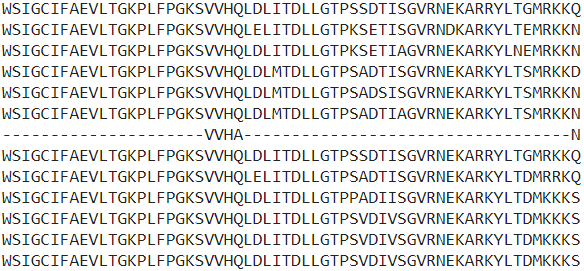


**LLE^R^KLLAFDPKDRPTAEEAL**


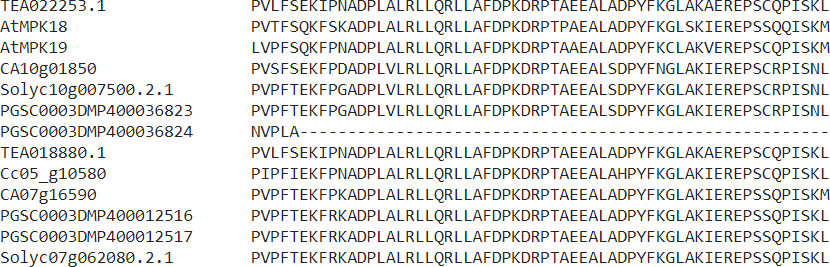

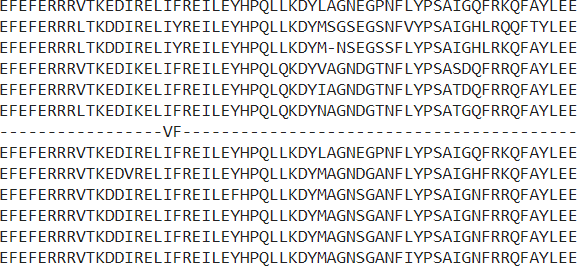

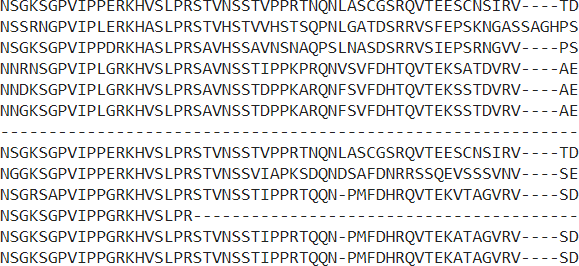


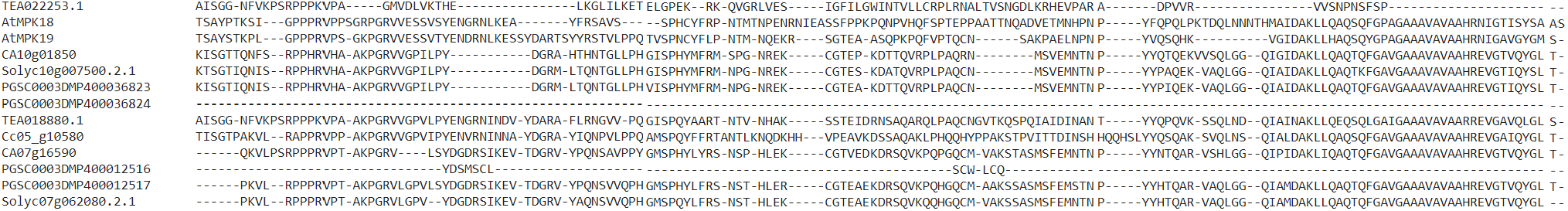


# Clade G

IGxGxYGxV IKKIxxxF


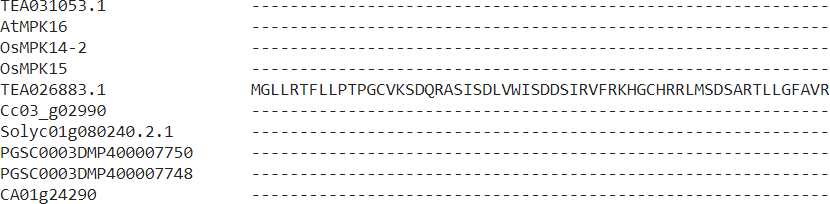

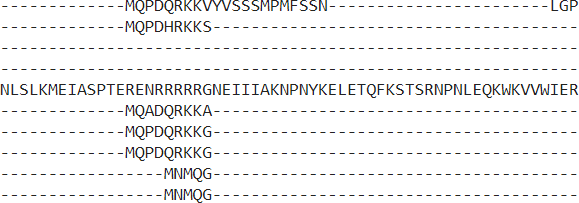

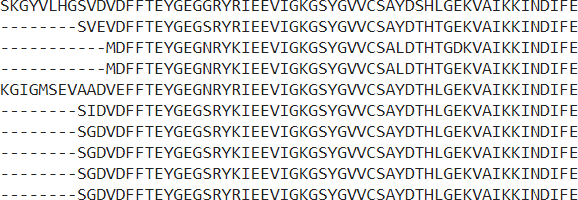


DAxRxLRE FxDIYxxxELM DLxxVI QxLRxLKYxH HRDLKPxN LxNxNCxLKIxDFGLAR TRWYRAPEL IDxWSVIGC


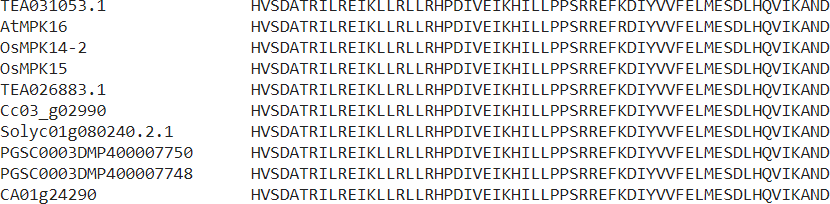

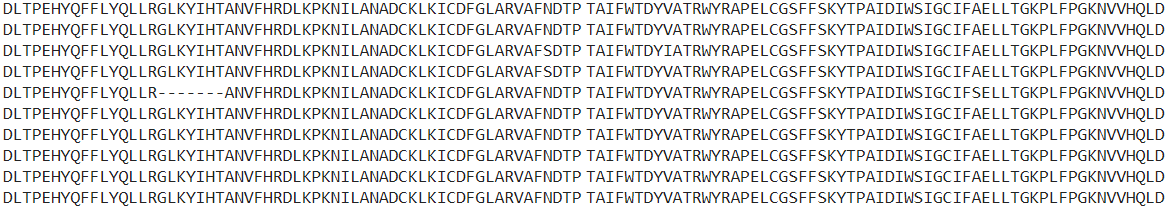


**LLE^R^KLLAFDPKDRPTAEEAL**


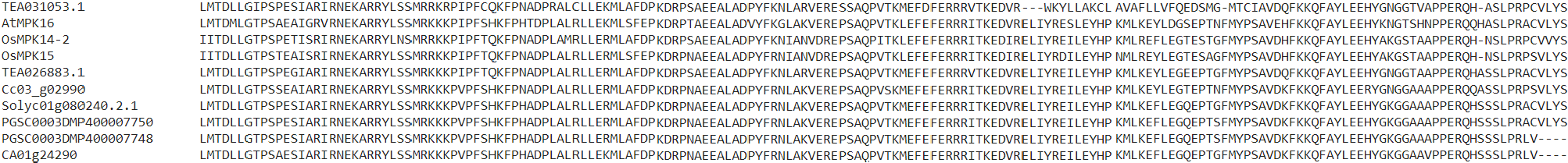

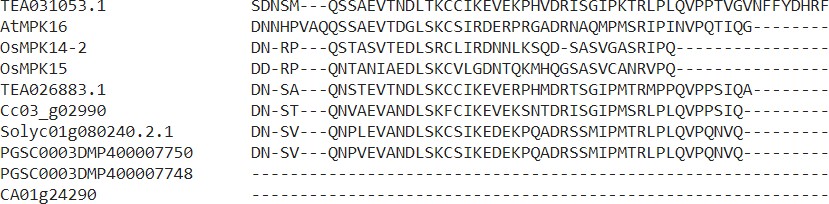

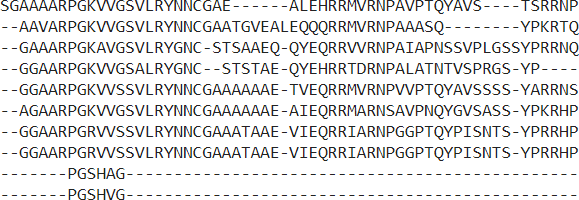

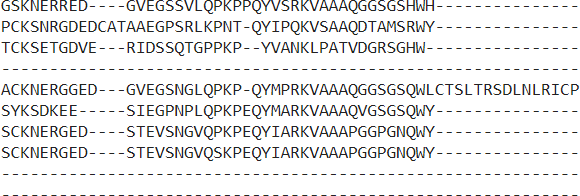


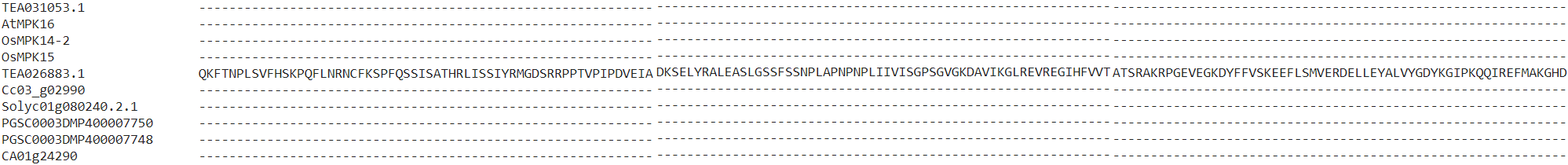


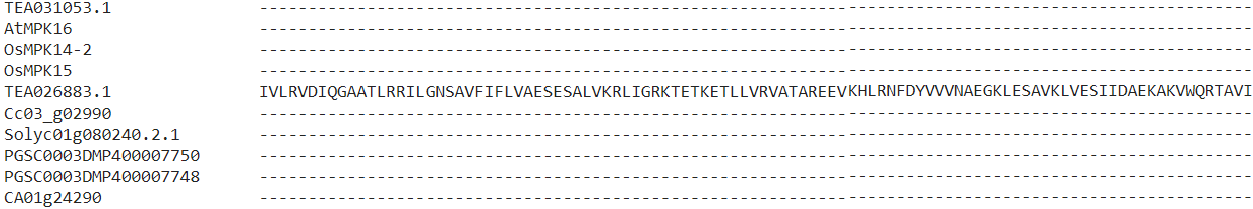


# Clade H

IGxGxYGxV IKKIxxxF DAxRxLRE


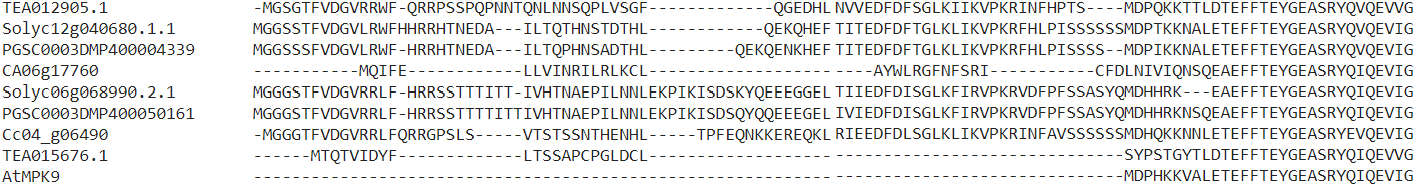

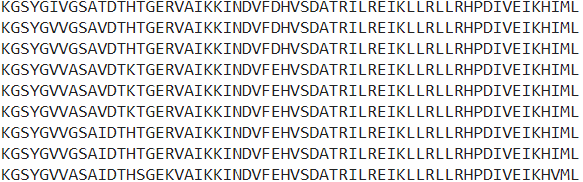


FxDIYxxxELM DLxxVI QxLRxLKYxH HRDLKPxN LxNxNCxLKIxDFGLAR TRWYRAPEL IDxWS^V^ GC

**I**


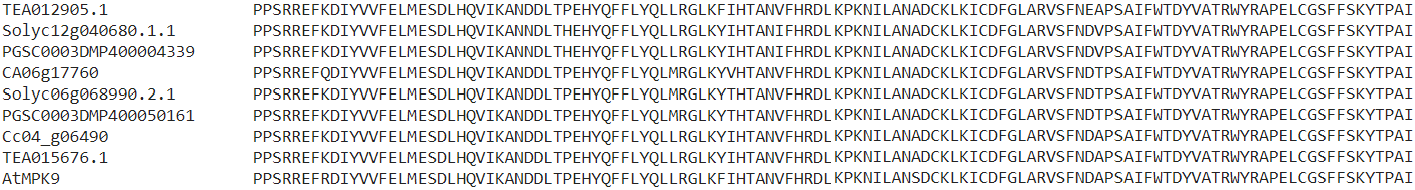

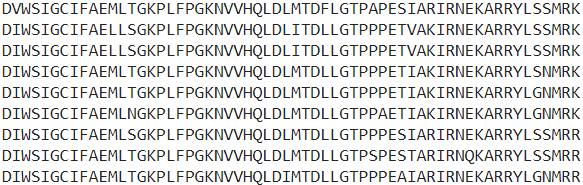


**LLE^R^KLLAFDPKDRPTAEE AL**


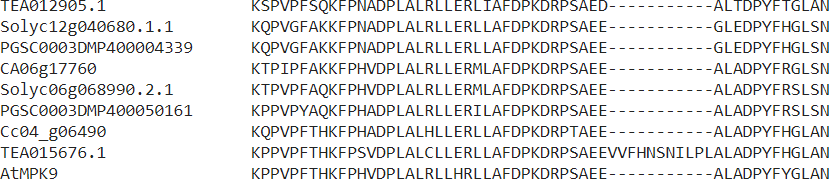

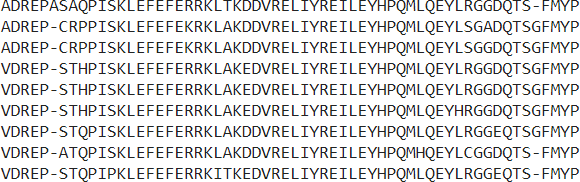

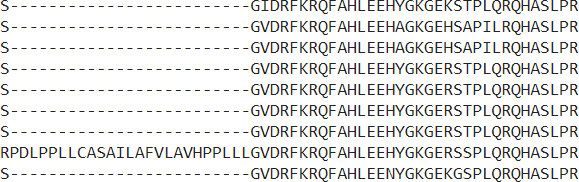


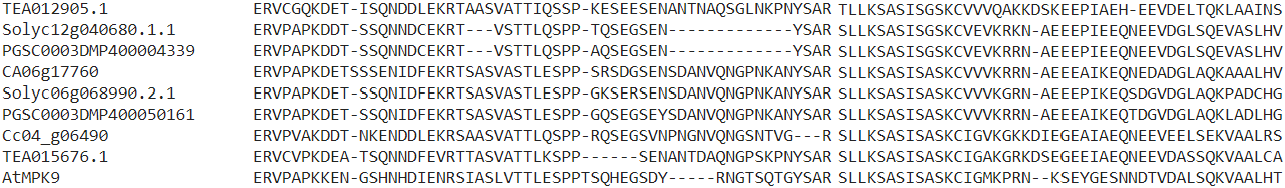


# Clade I

IGxGxYGxV IKKIxxxF DAxRxLRE F


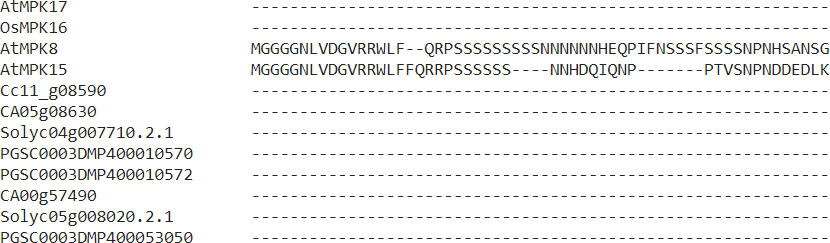

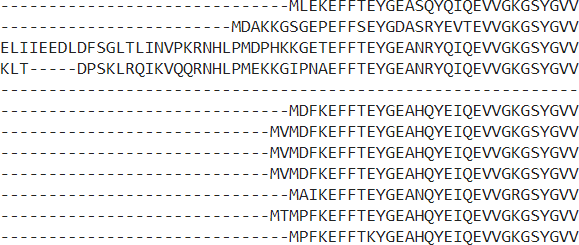

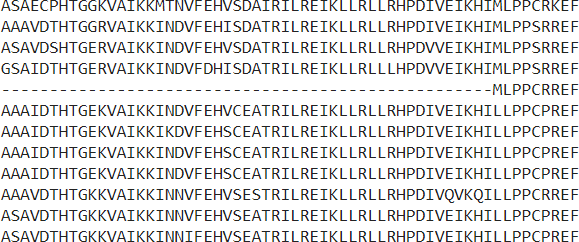


xDIYxxxELM DLxxVI QxLRxLKYxH HRDLKPxN LxNxNCxLKIxDFGLAR TRWYRAPEL IDxWS^V^ GC

**I**


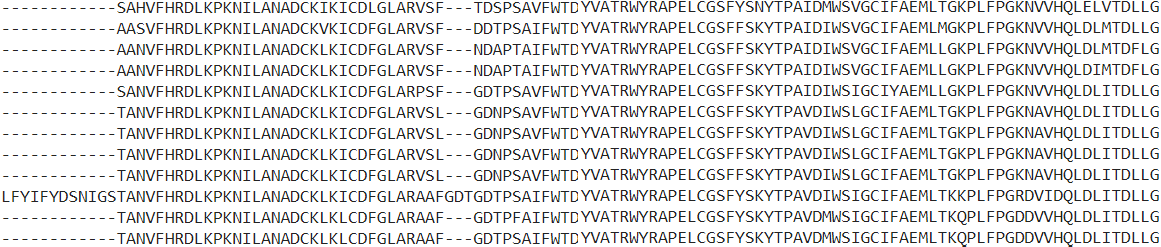

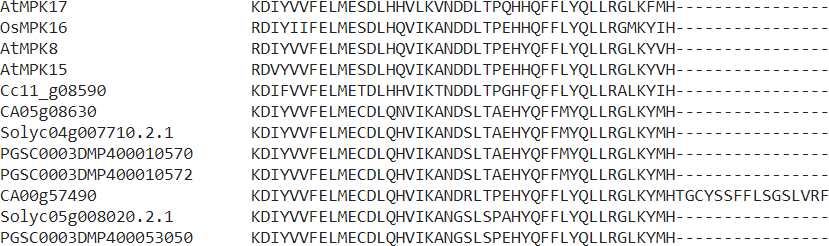


**LLE^R^KLLAFDPKDRPTAEEAL**


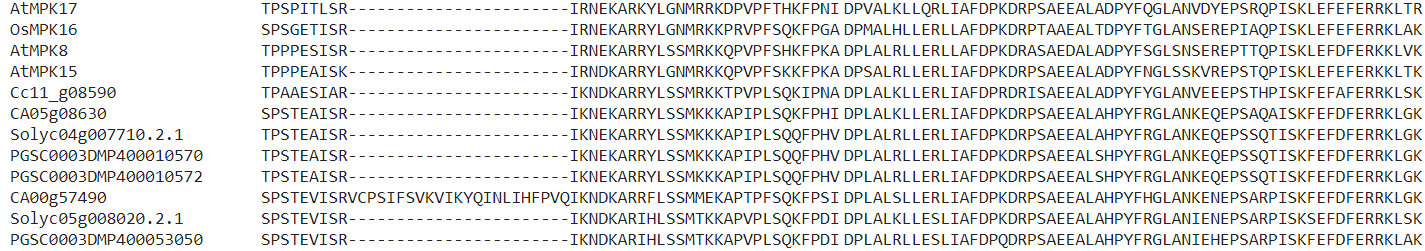

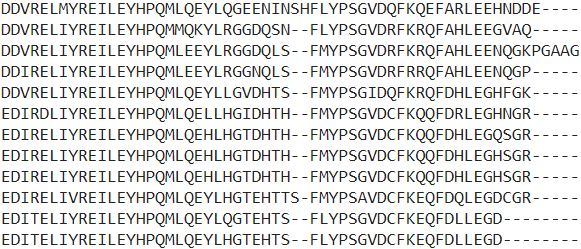

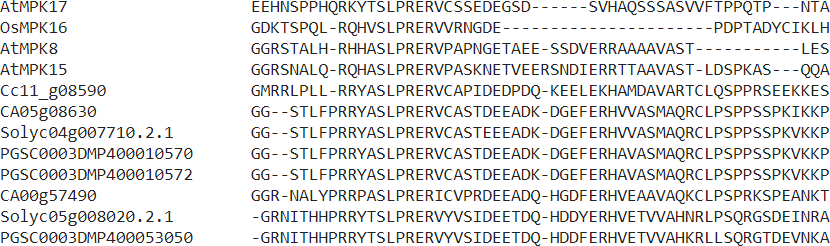

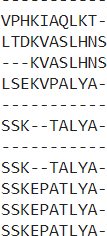

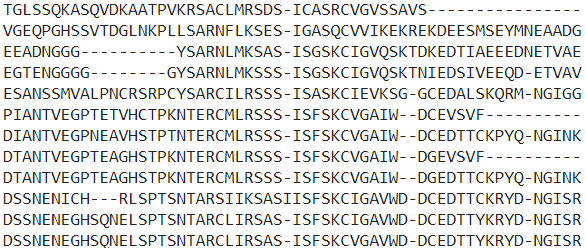


# Clade J


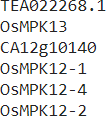


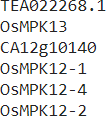


VI QxLRxLKYxH

IGxGxYGxV IKKIxxxF DAxRxLRE FxDIYxxxELM

HRDLKPxN LxNxNCxLKIxDFGLAR TRWYRAPEL IDxWS^V^ GC


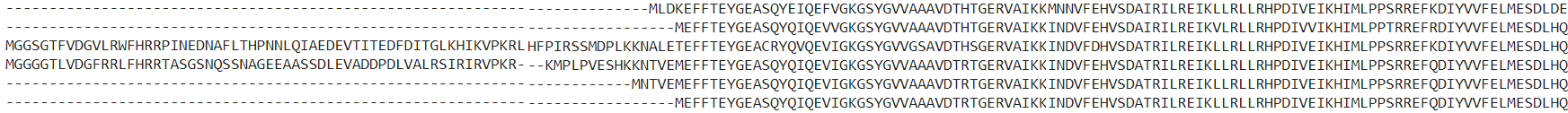

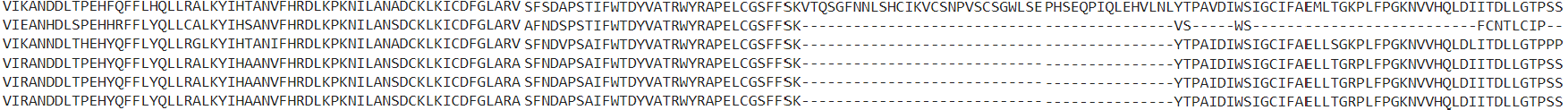


**I**

**LLE^R^ LLAFDPKDRPTAEEAL**

**K**

DLxx


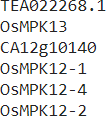


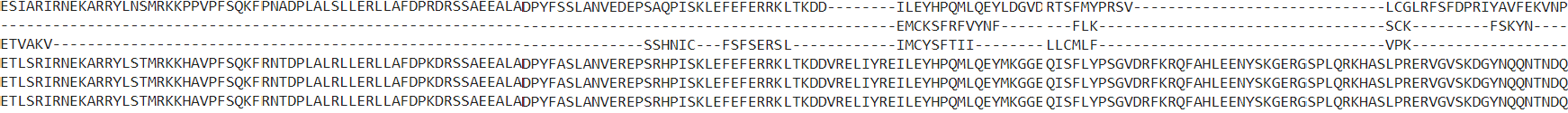


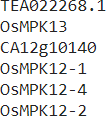


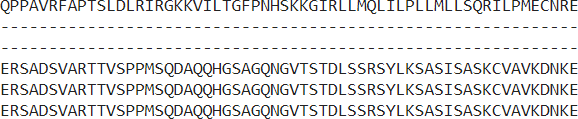

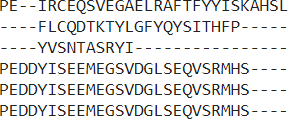


**Supplementary Fig. S7** **Alignment of domains in MPKs.** All the MPK protein sequences were subjected to alignment by MUSCLE tool owing to their sequence diversities. Sequences that are highlighted are ATP binding signature, marked in blue, the catalytic C loop, marked in light red colour, the activation T loop, marked in green colour, CD domain, marked in light blue colour. Clades D to J show sequence derivations from the T(E/D)Y activation loop and are marked in a lighter shade of green colour.
