# Supplementary material for: MAPK cascade gene family in Camellia sinensis: In-silico identification, expression profiles and regulatory network analysis
Source: BMC Genomics. 2020 Sep 7;21:613. doi: 10.1186/s12864-020-07030-x (PMC7487466; doi:10.1186/s12864-020-07030-x)
Supplement: Supplementary file 4 — Additional file 4: Figure S8. Alignment of domains in MKKs. All the MKK protein sequences were subjected to alignment by MUSCLE tool owing to their sequence diversities. Sequences that are highlighted are ATP binding signature, marked in blue that consists the P loop consensus sequence (GxGxxG), the catalytic C loop, marked in light red colour that consists the DΨK consensus, the activation T loop, marked in green colour and NTF2 domain marked in greyish colour. Clade C and D show sequence derivations from the S/TxxxxxS/T activation loop and are marked in a lighter shade of green colour. [file 12864_2020_7030_MOESM4_ESM.docx]

**Additional MKK domains**

**Clade A f**


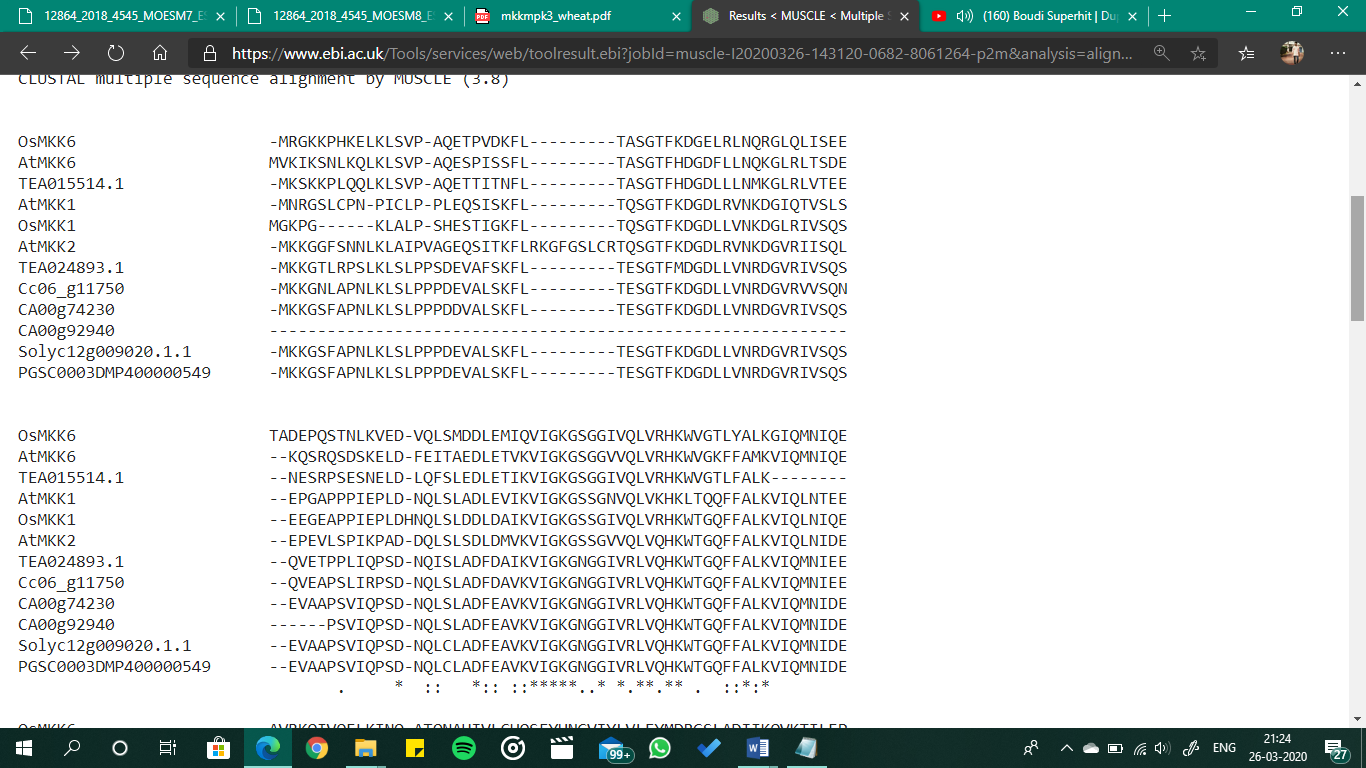

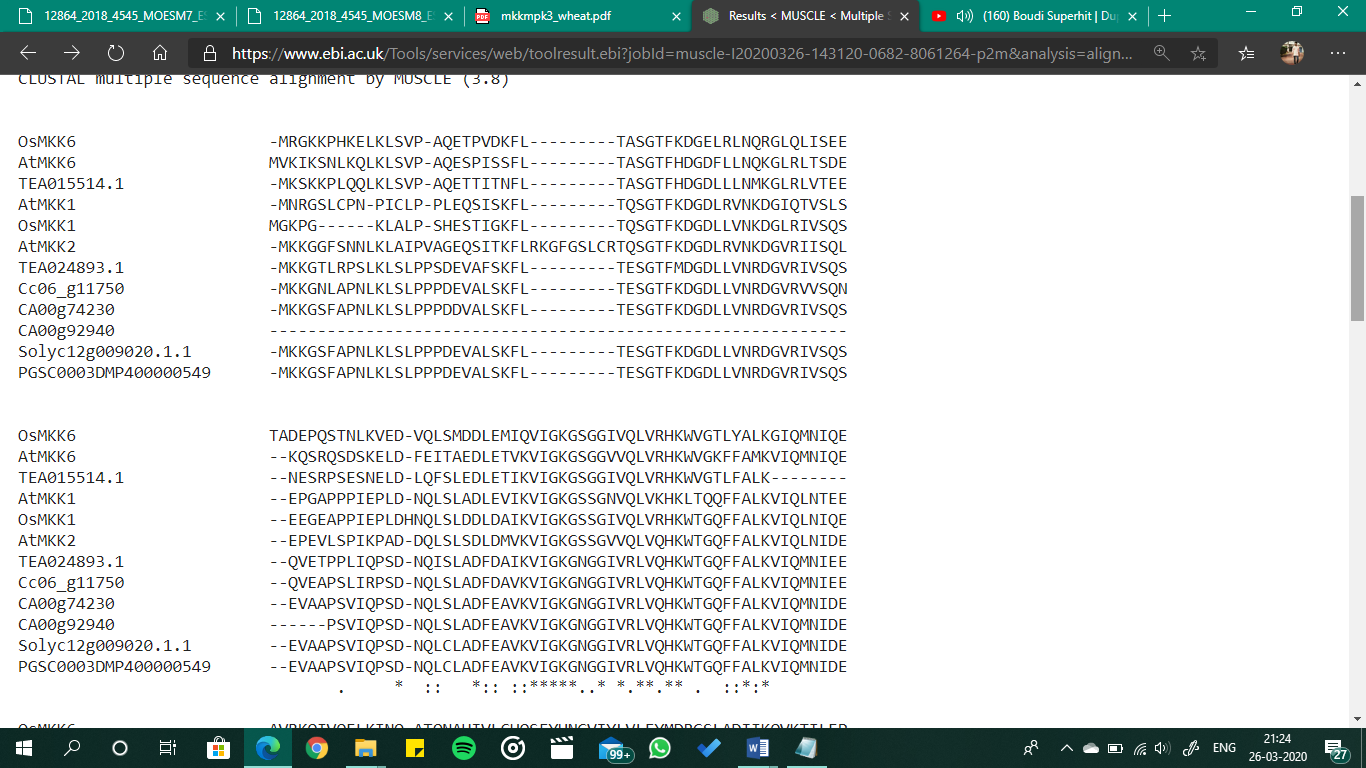

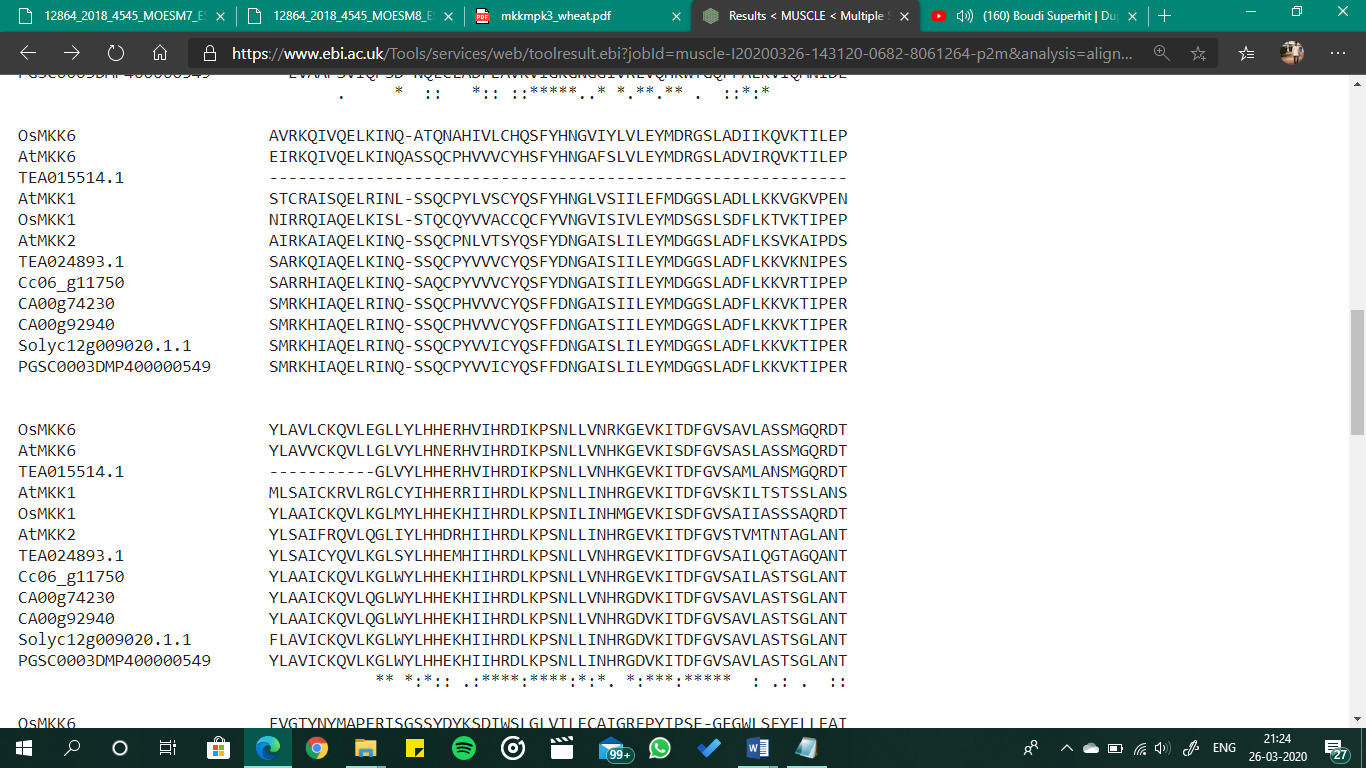

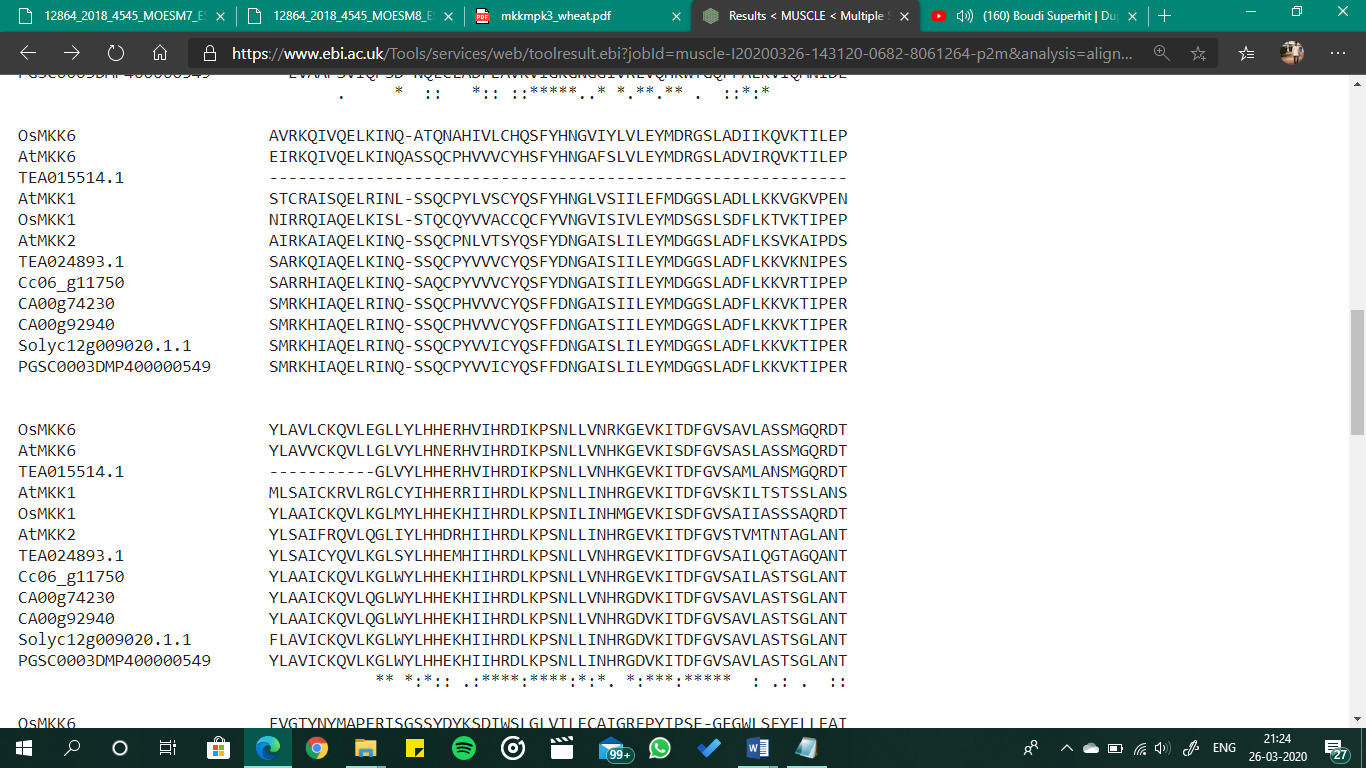

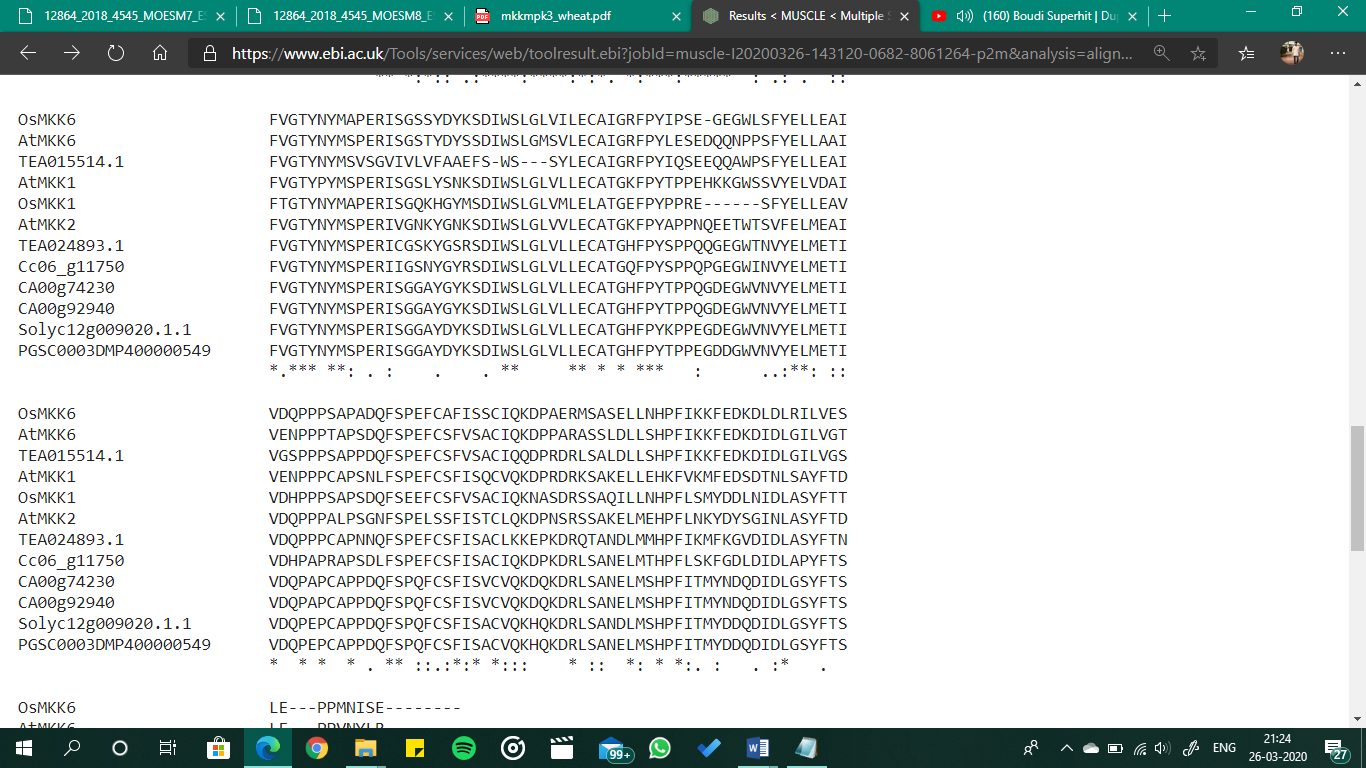

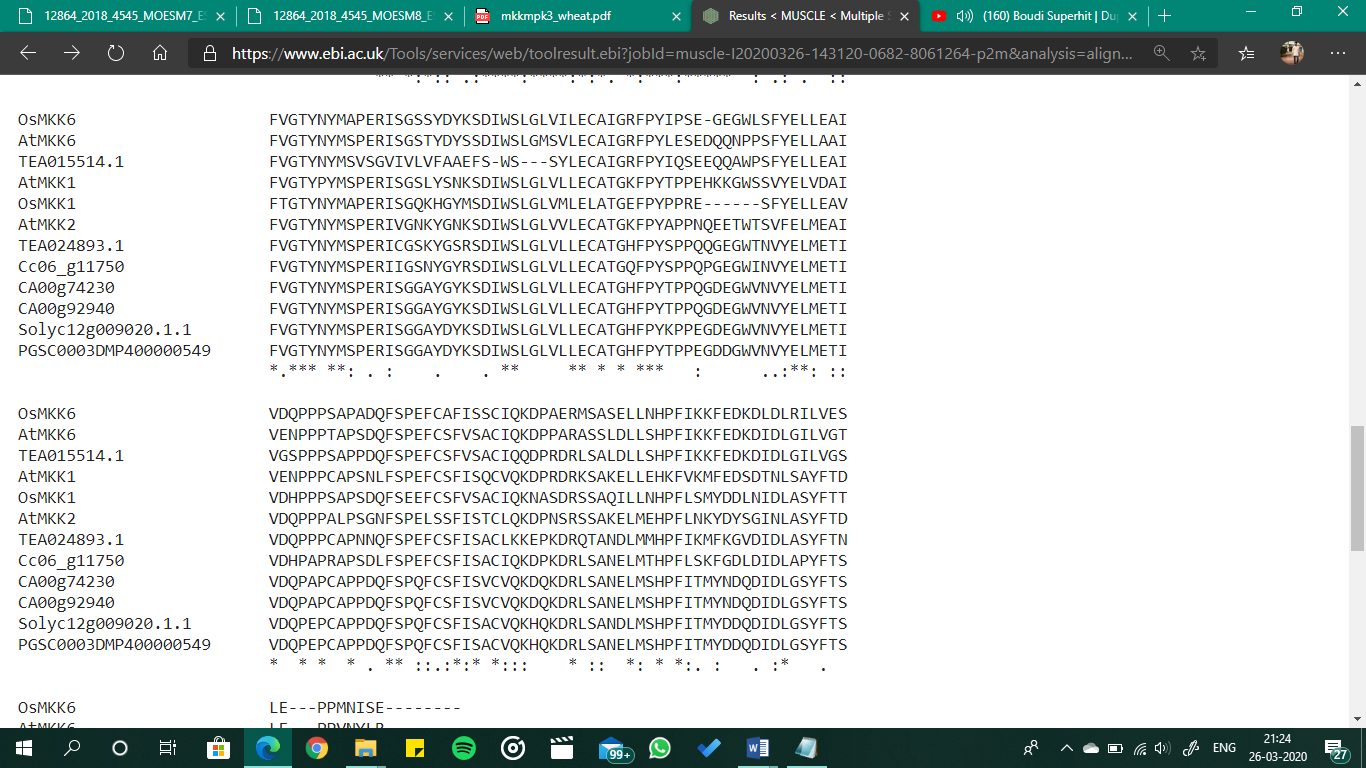


**GxGxxG**

**DΨK**


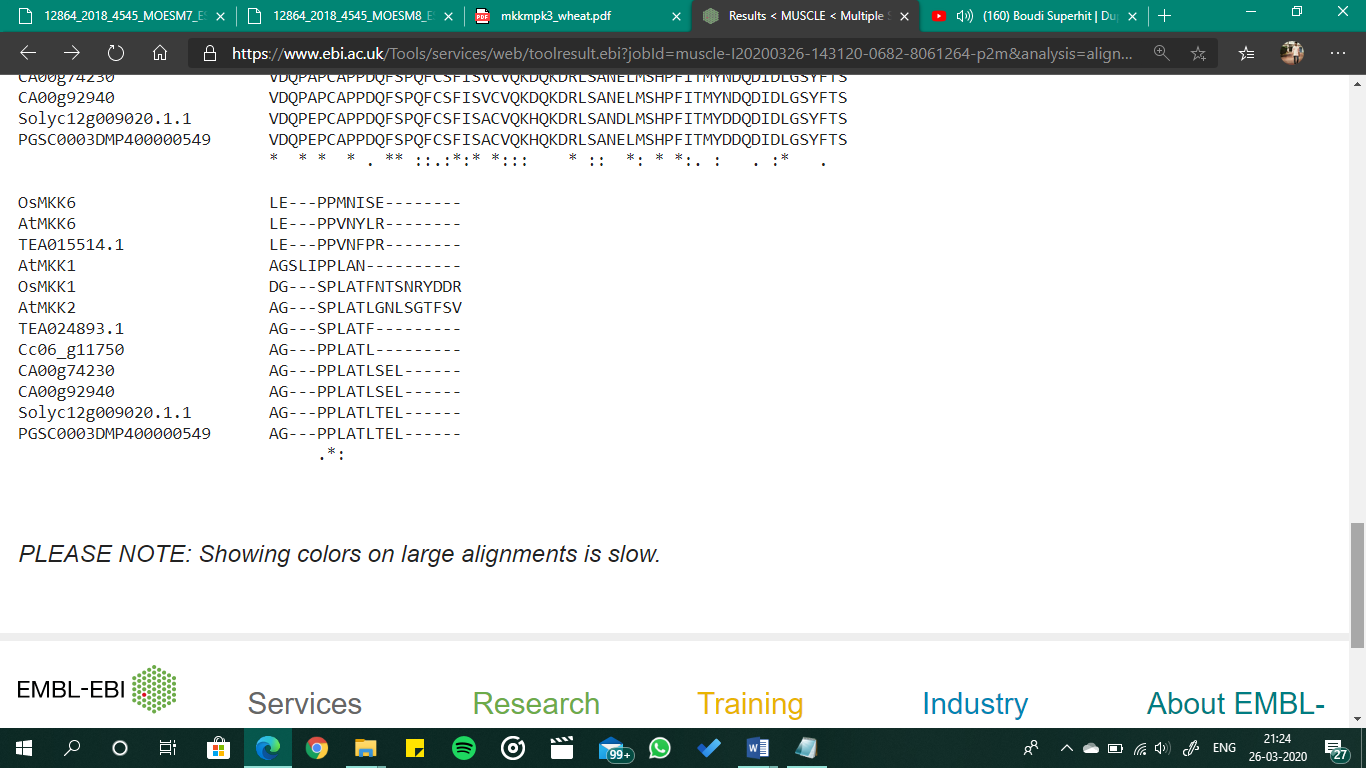


**Clade B f**


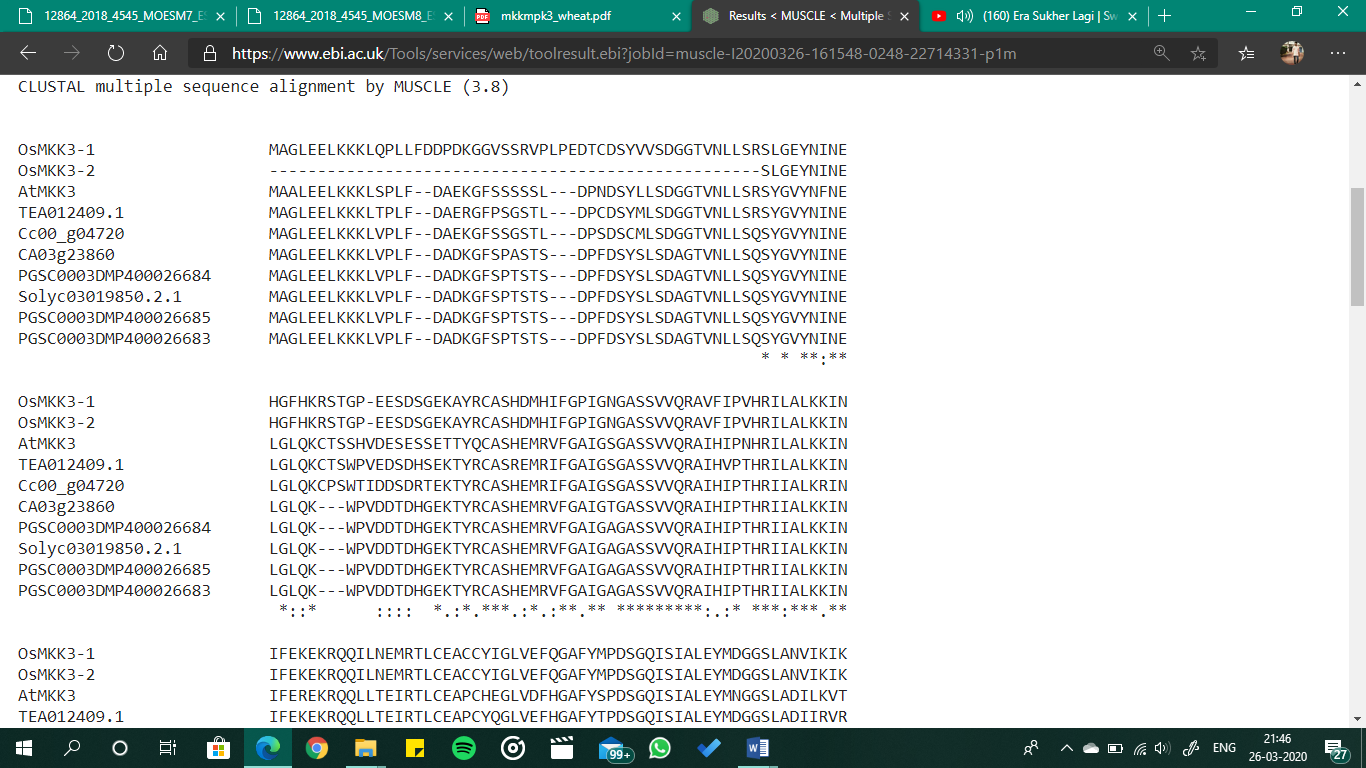

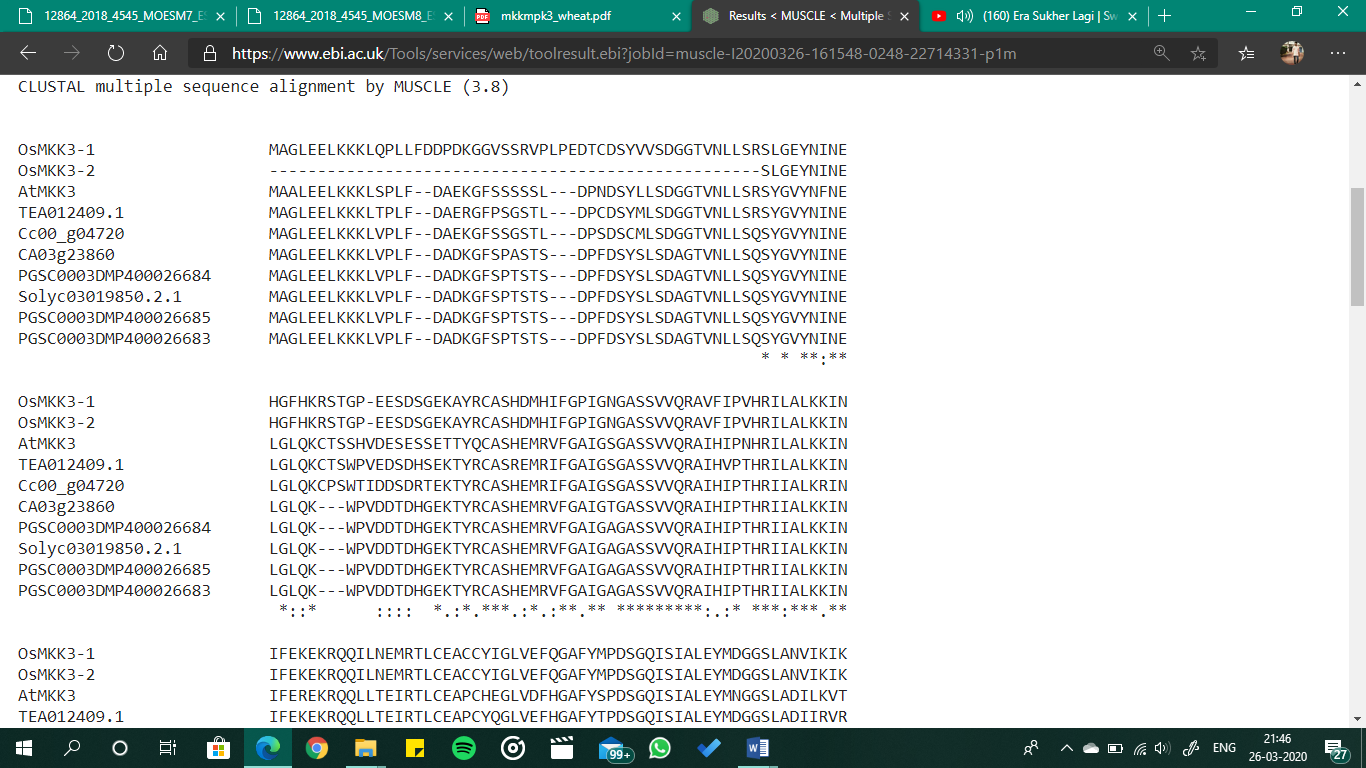

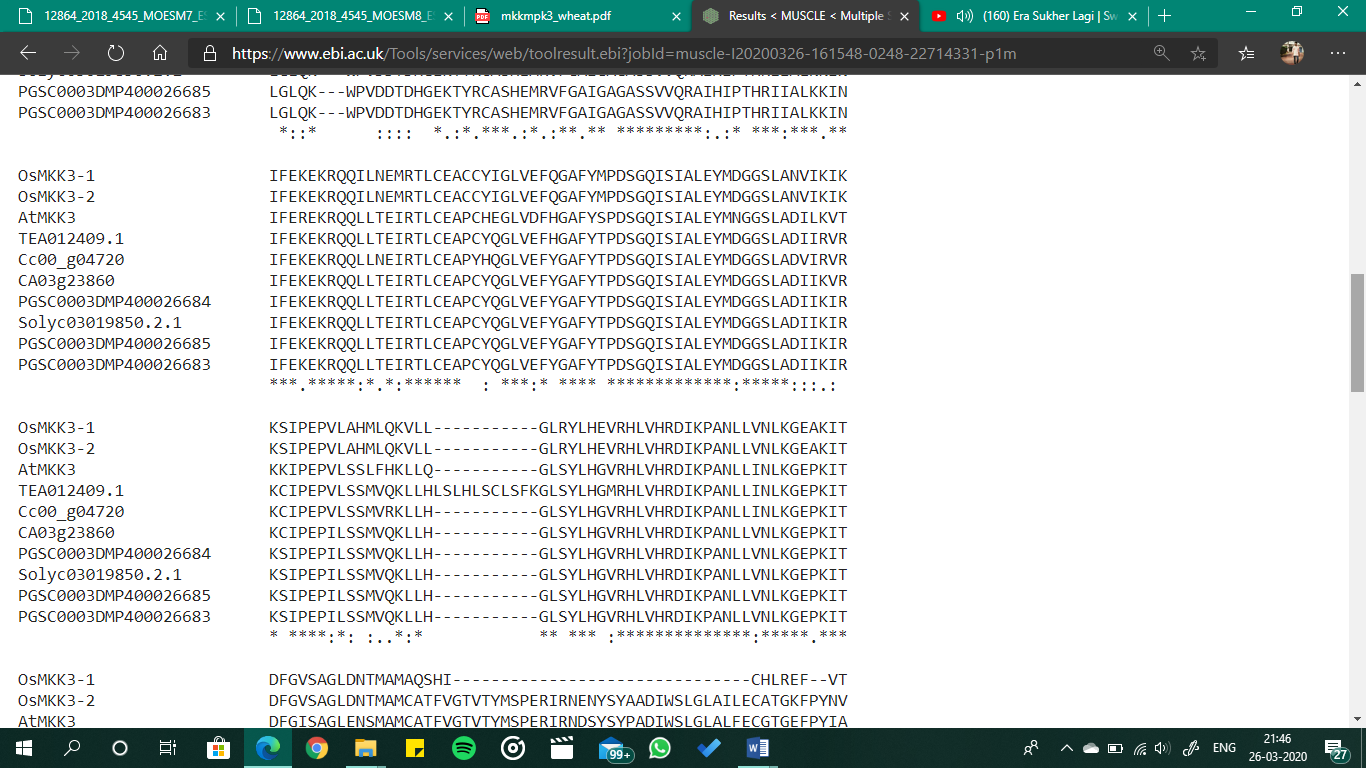

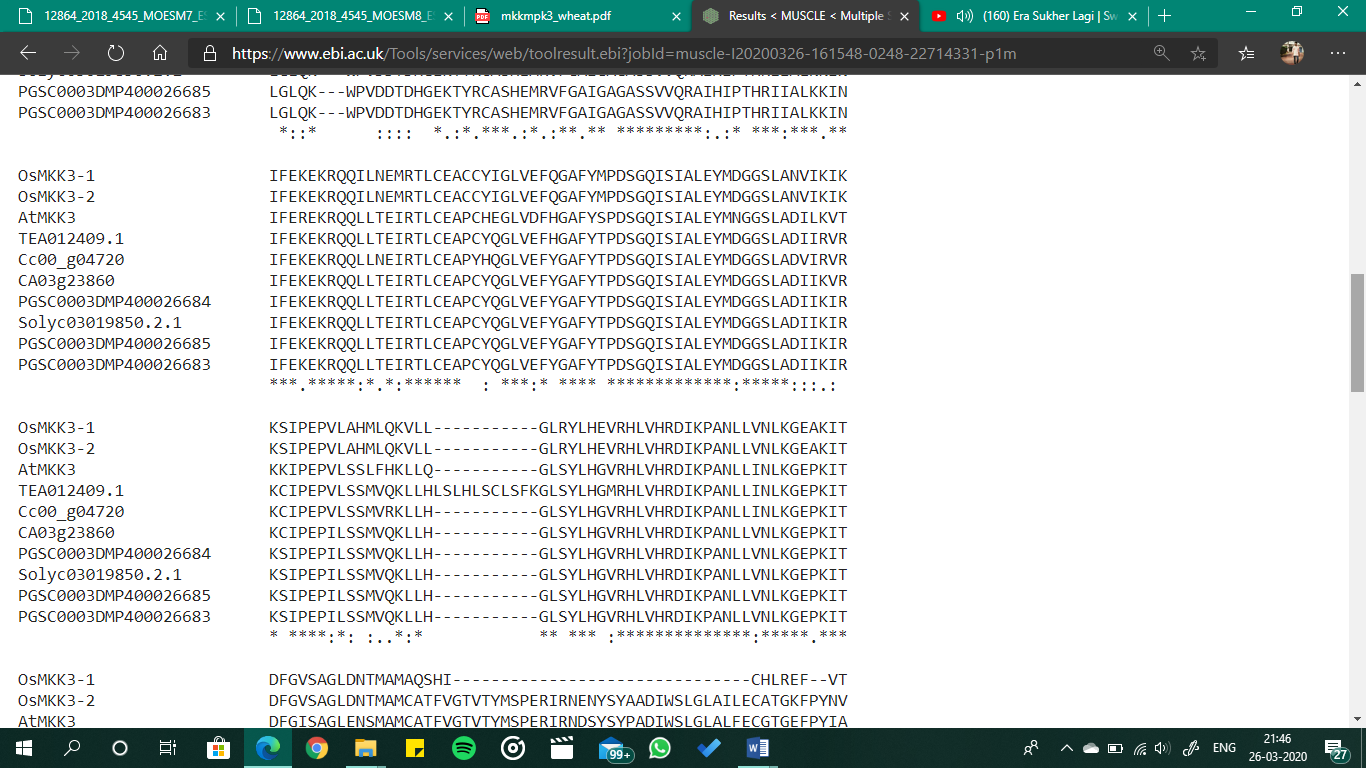


**GxxGxG**

**DΨK**


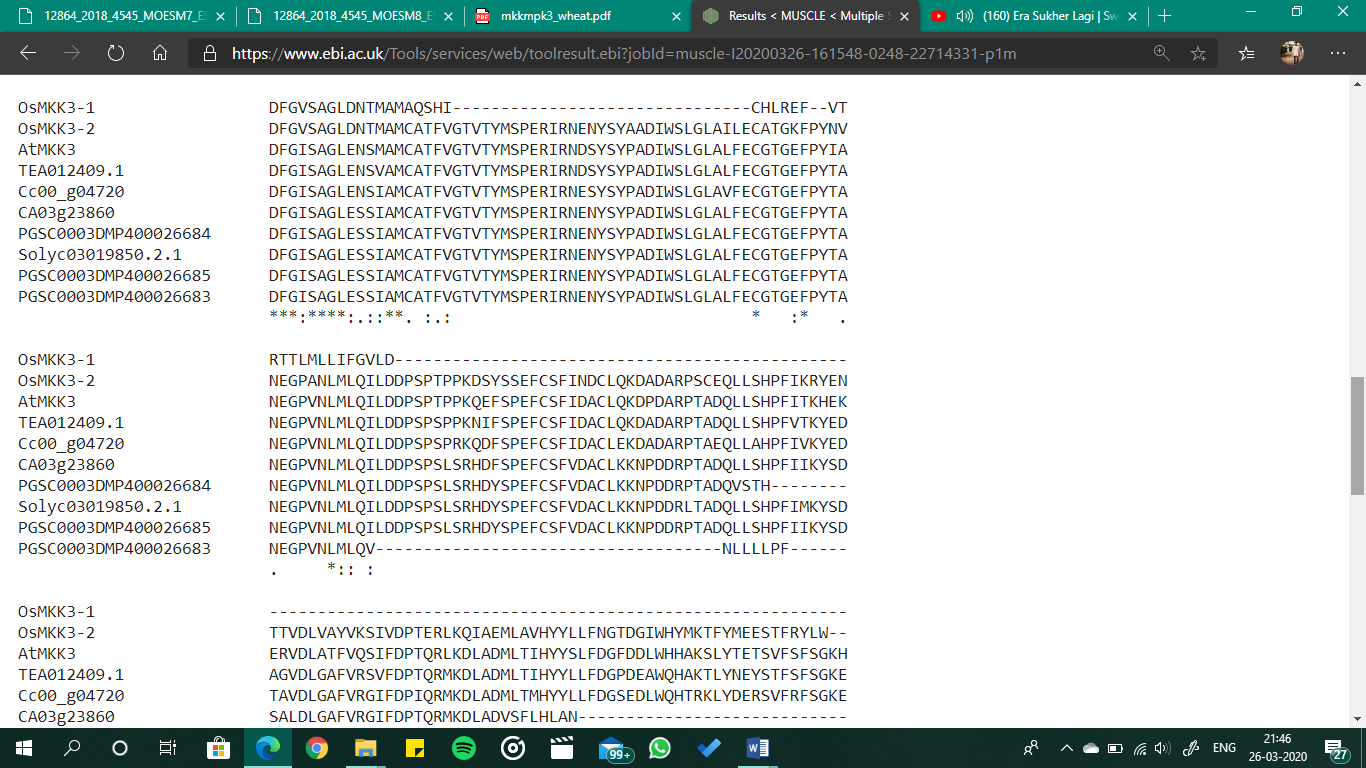

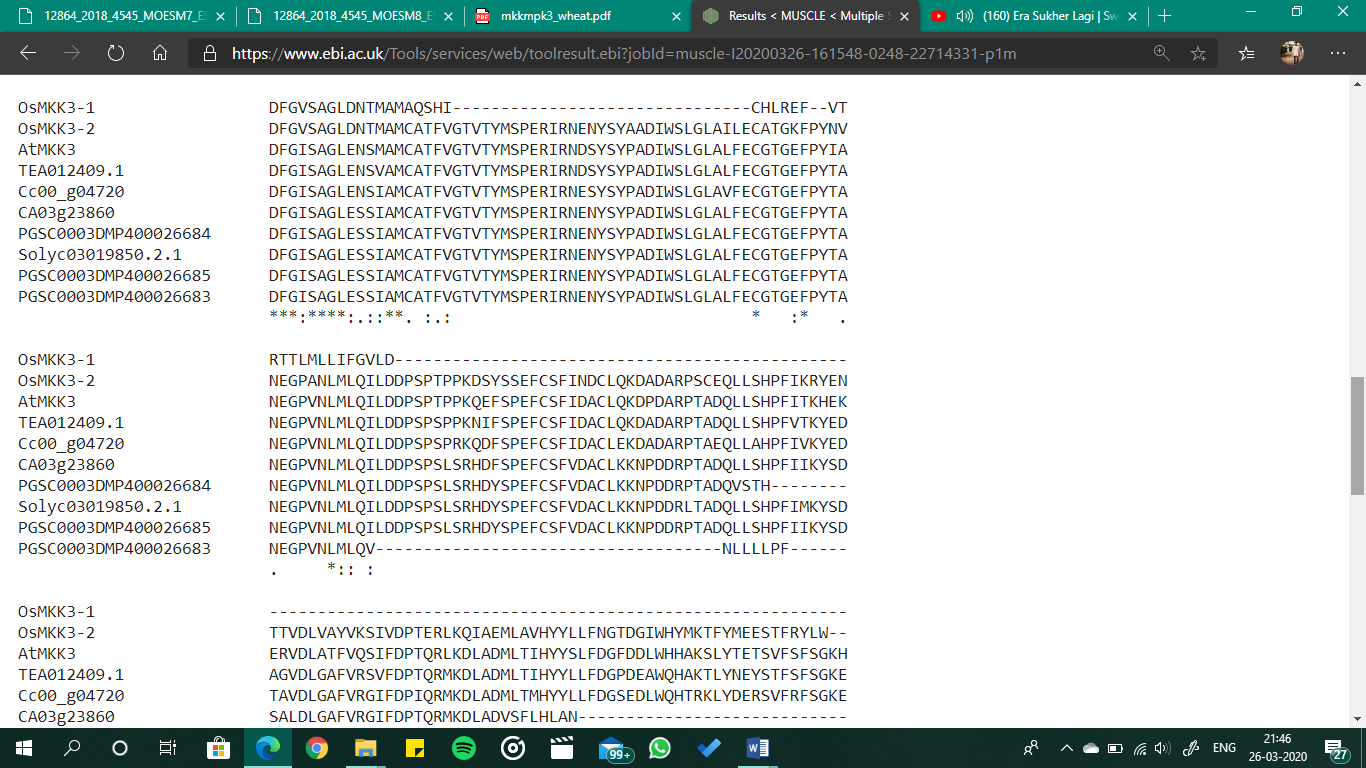

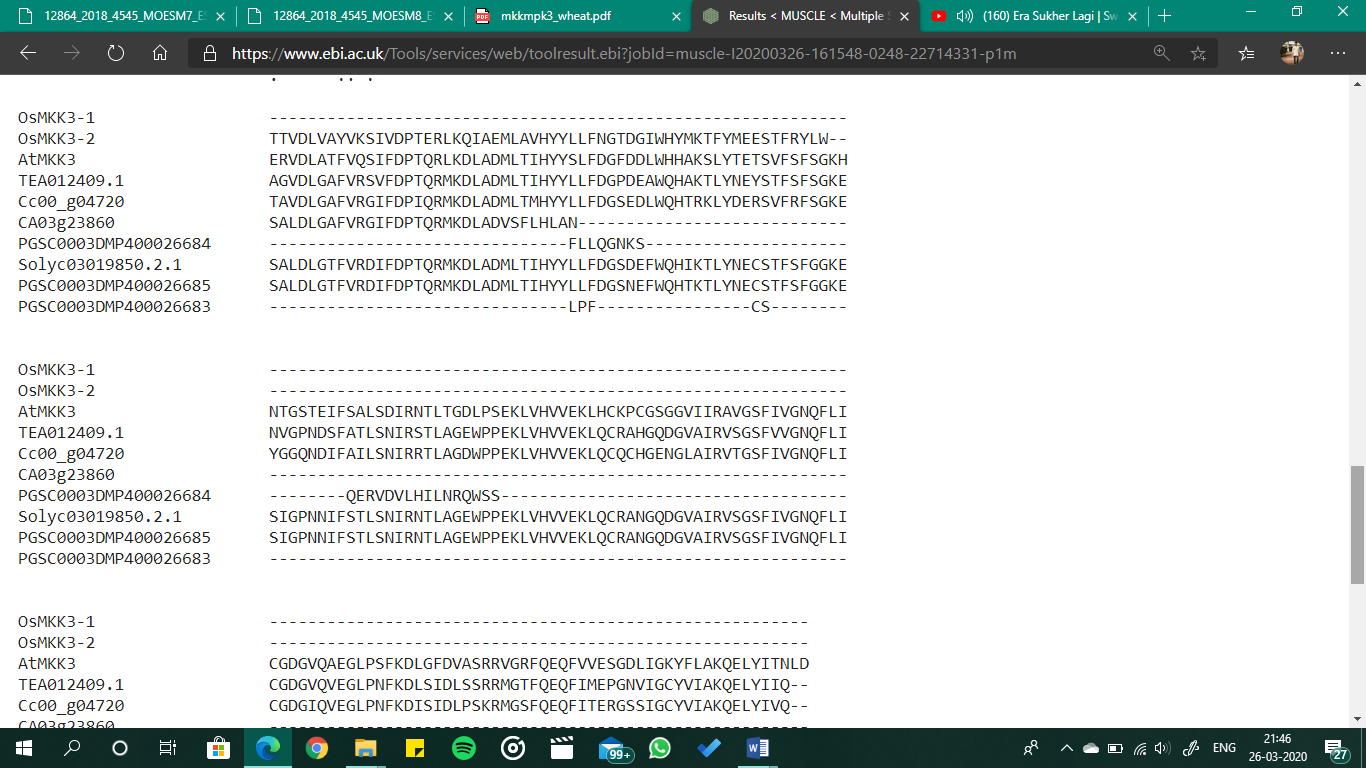

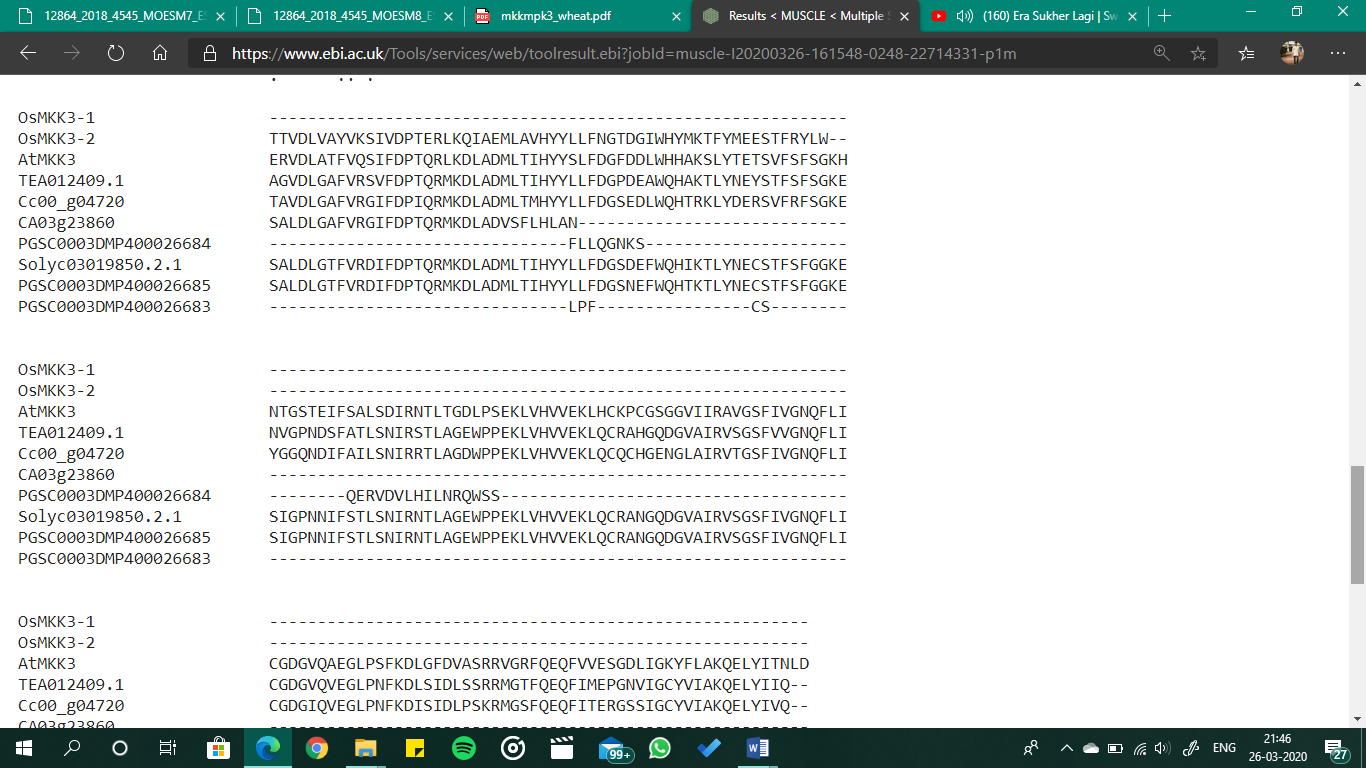

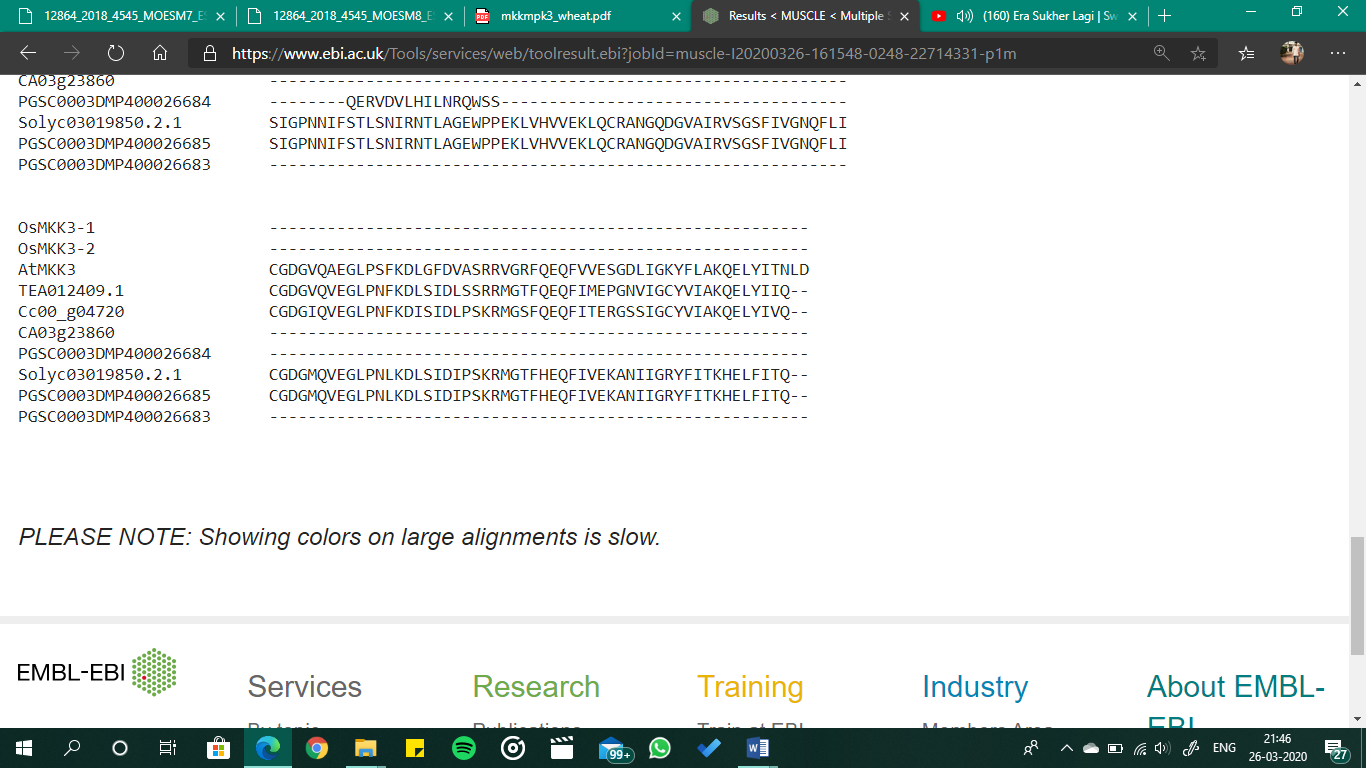


**Clade C f**


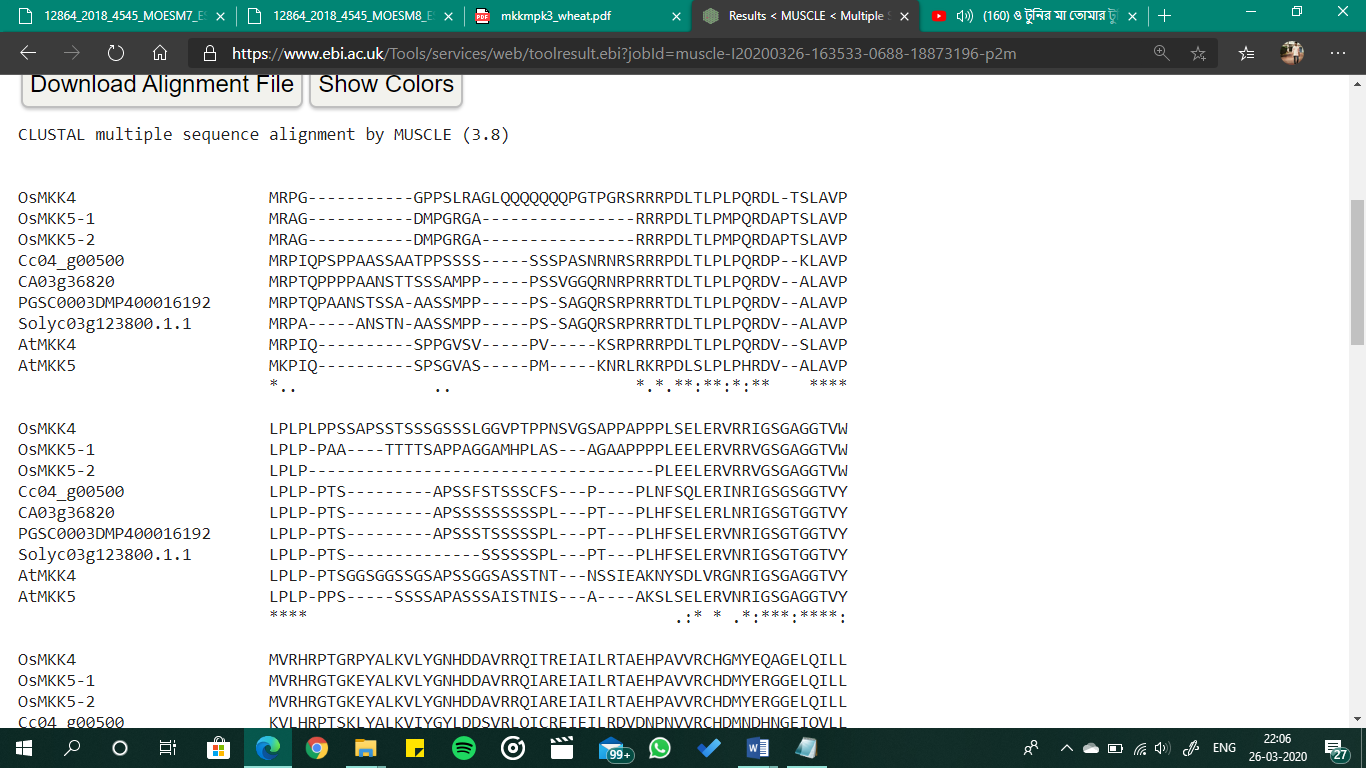

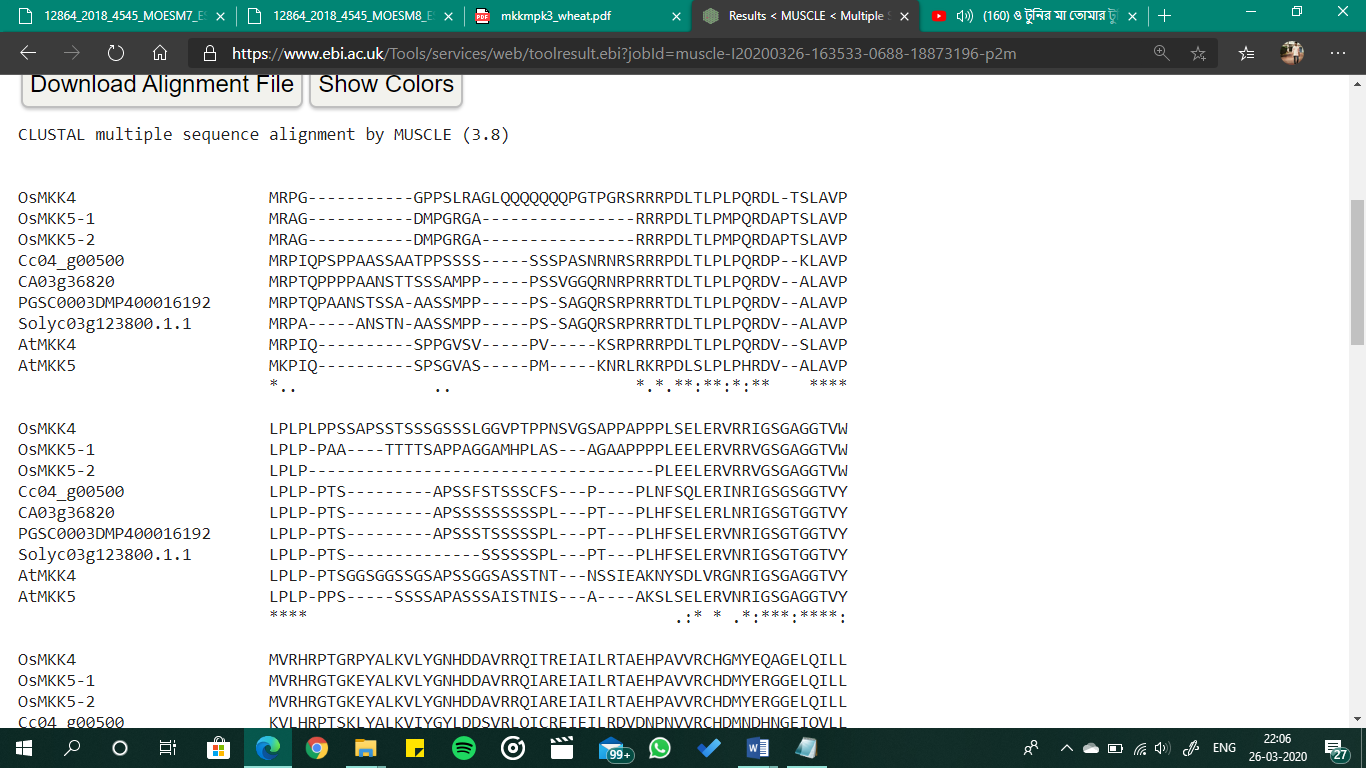

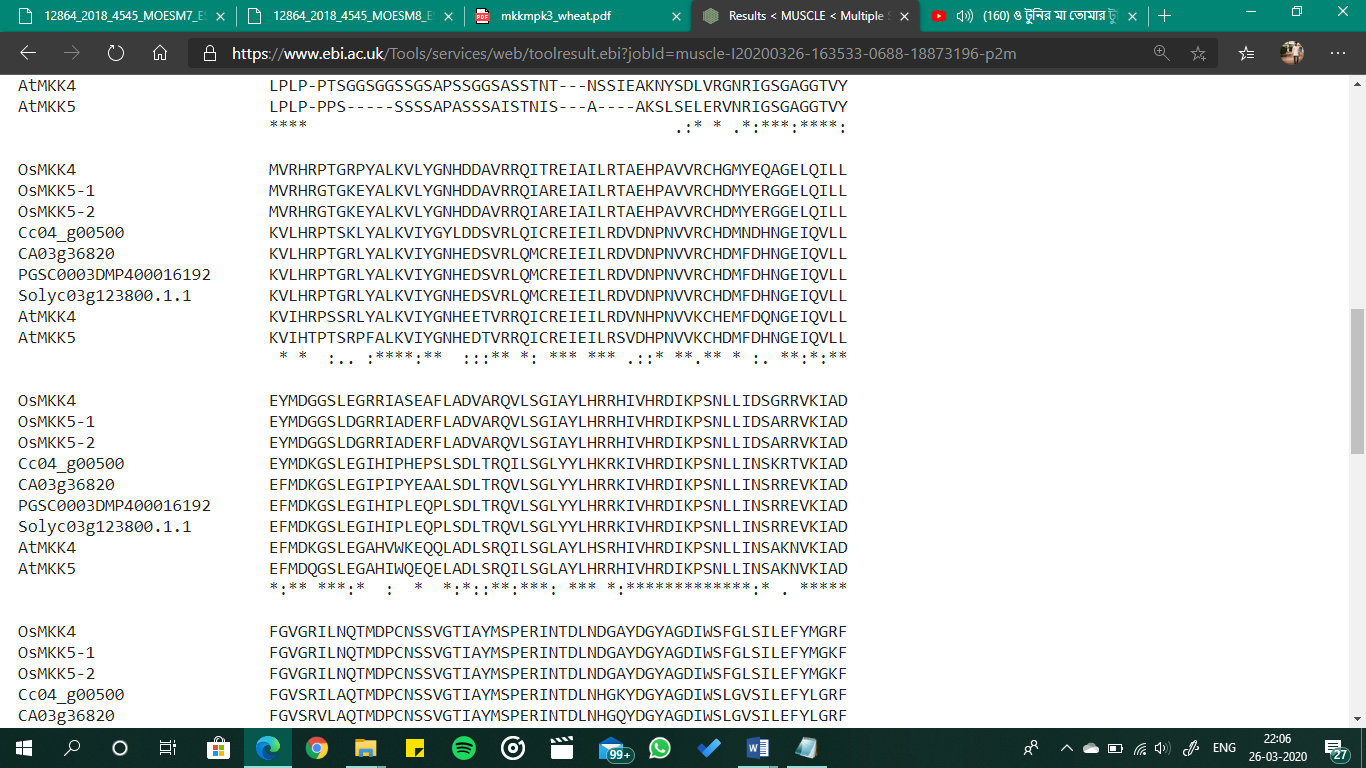

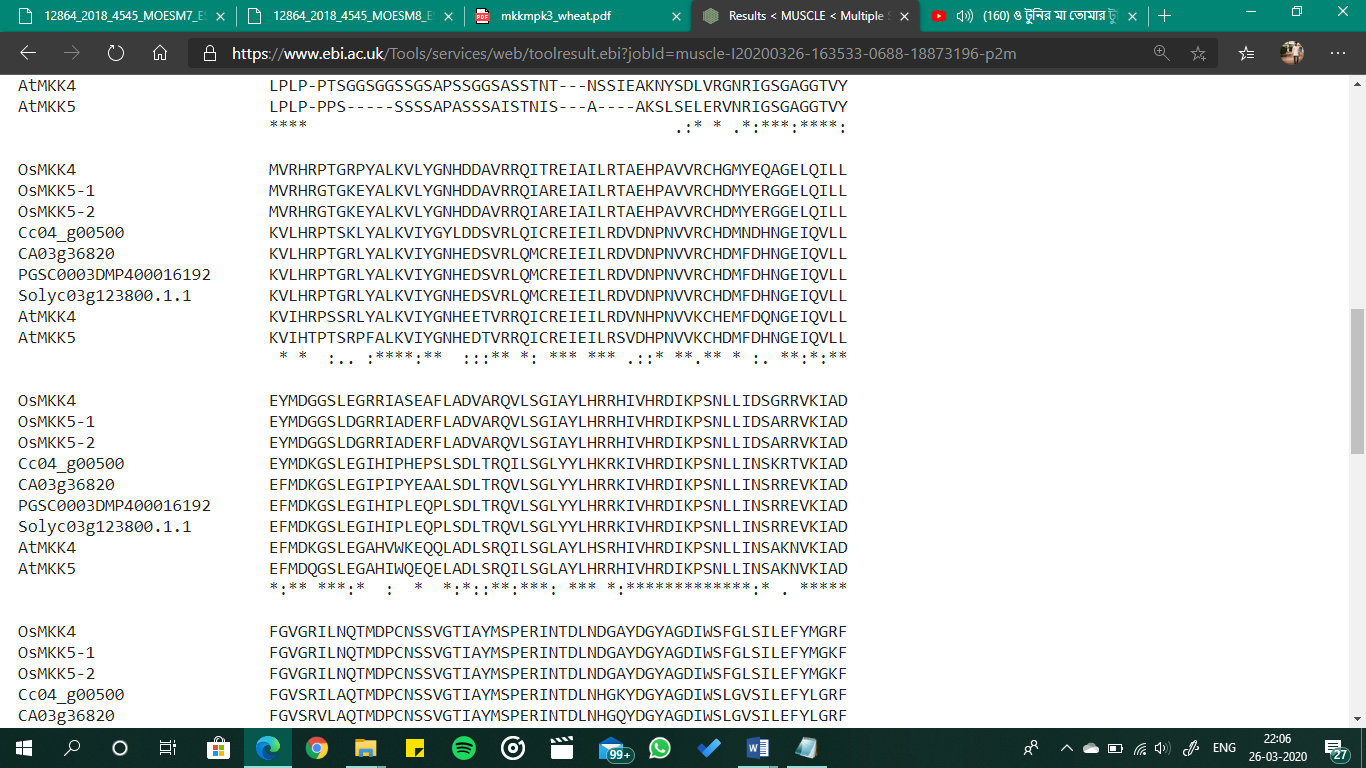

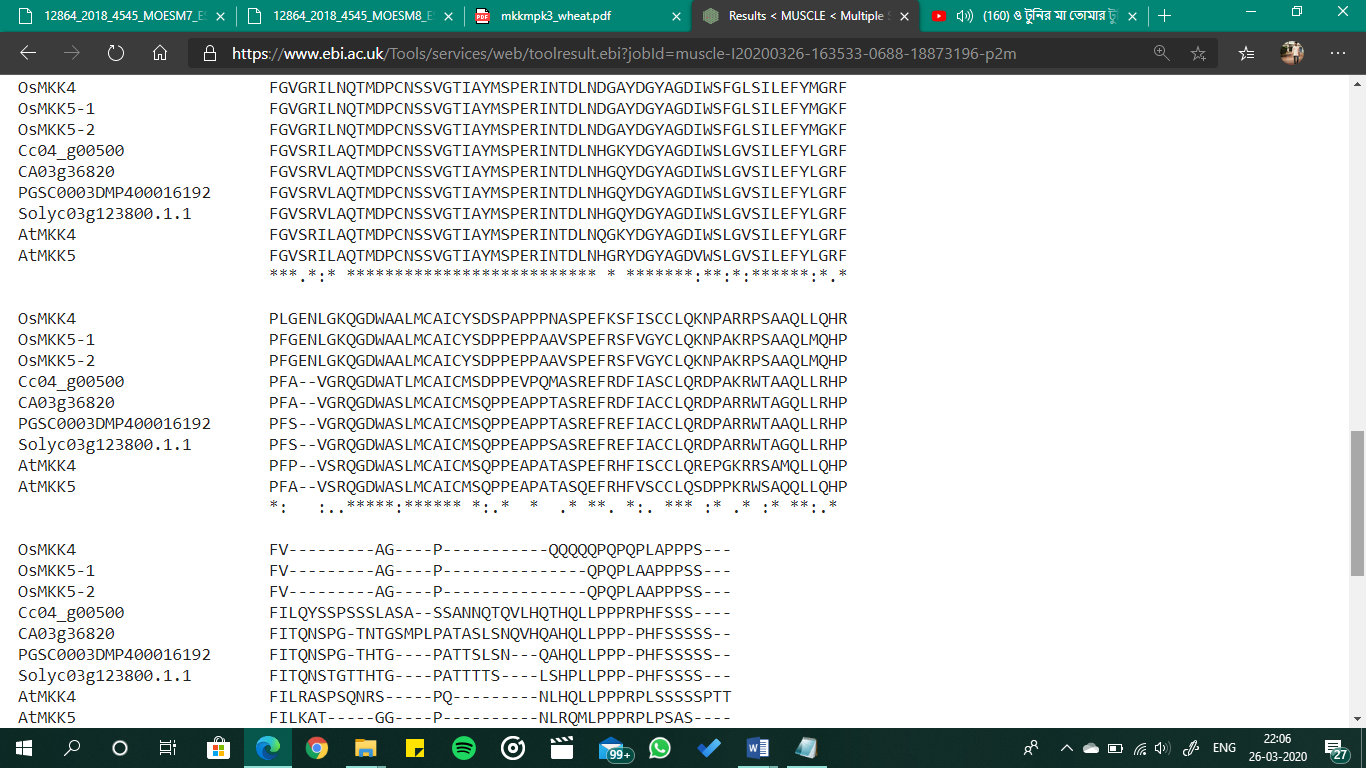

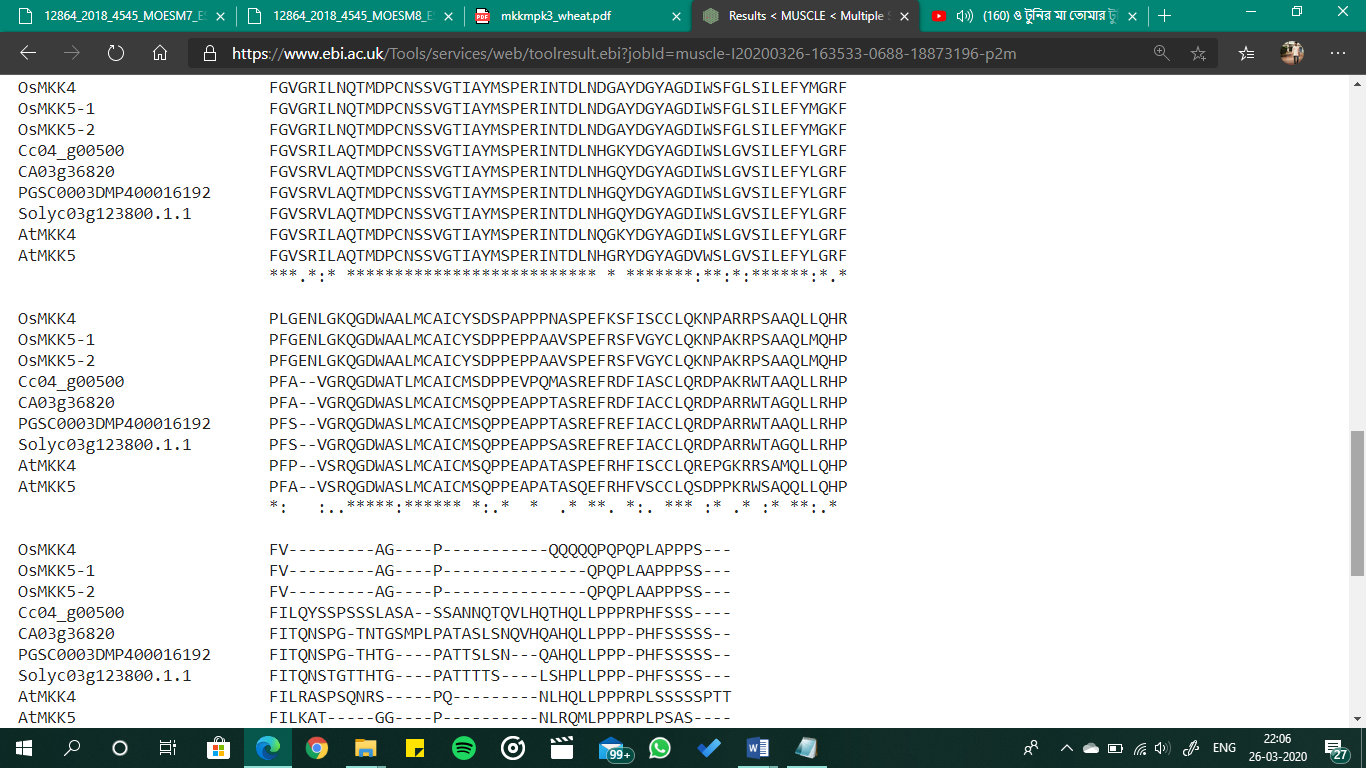

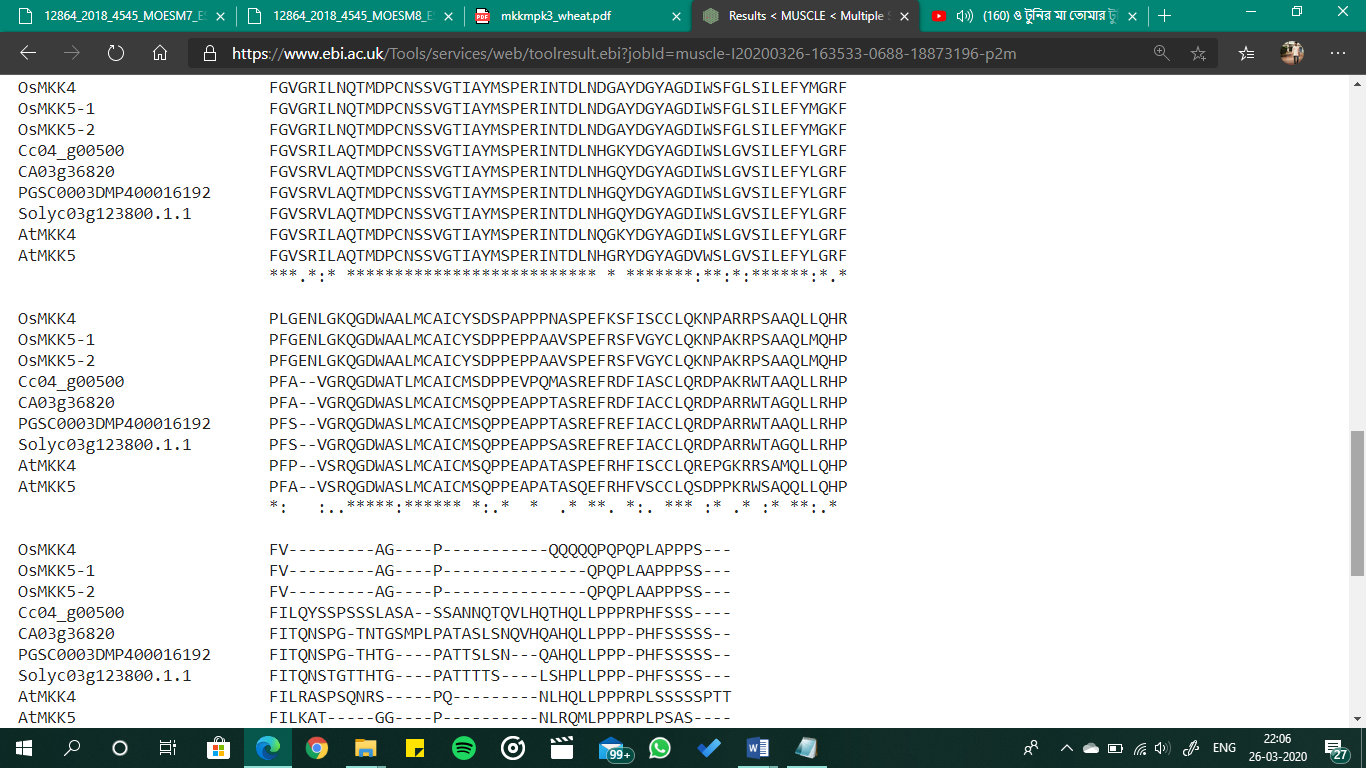


**GxGxxG**

**DΨK**

**Clade D f**


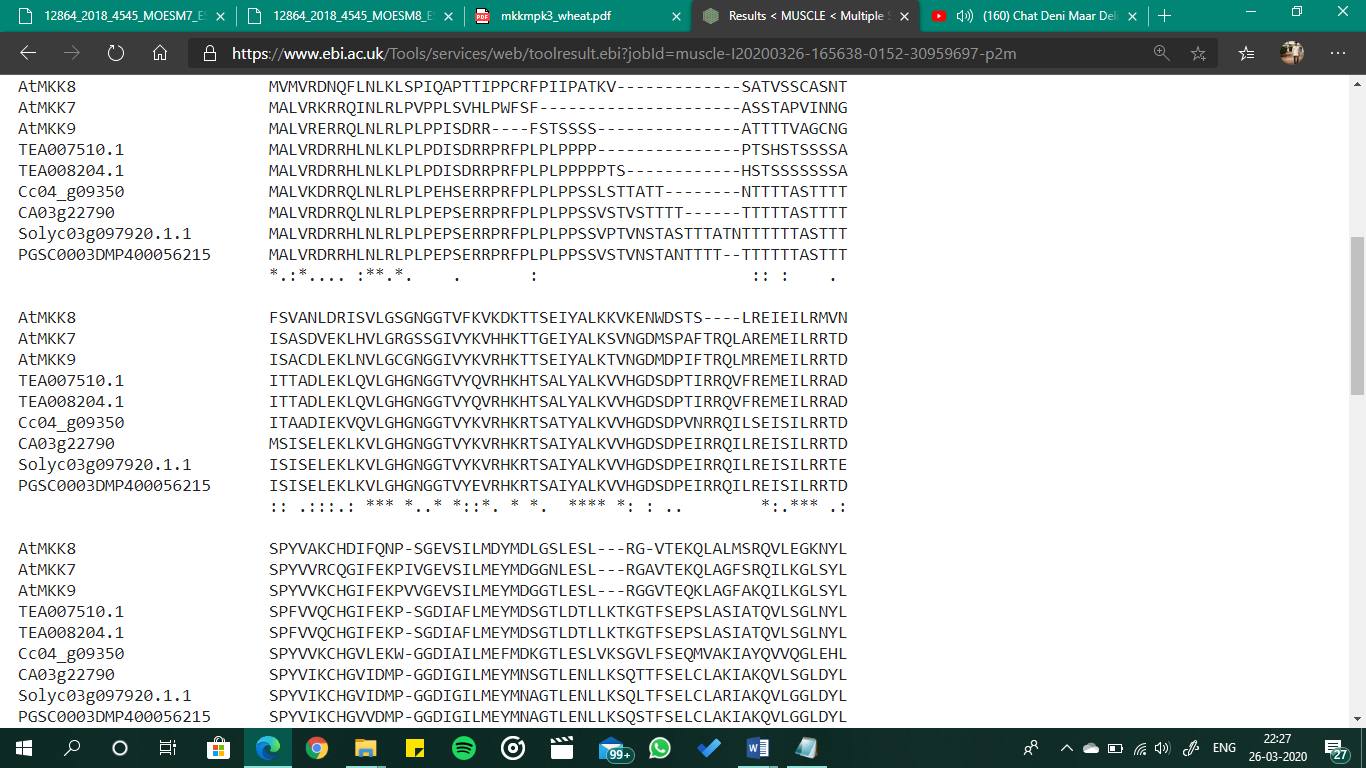

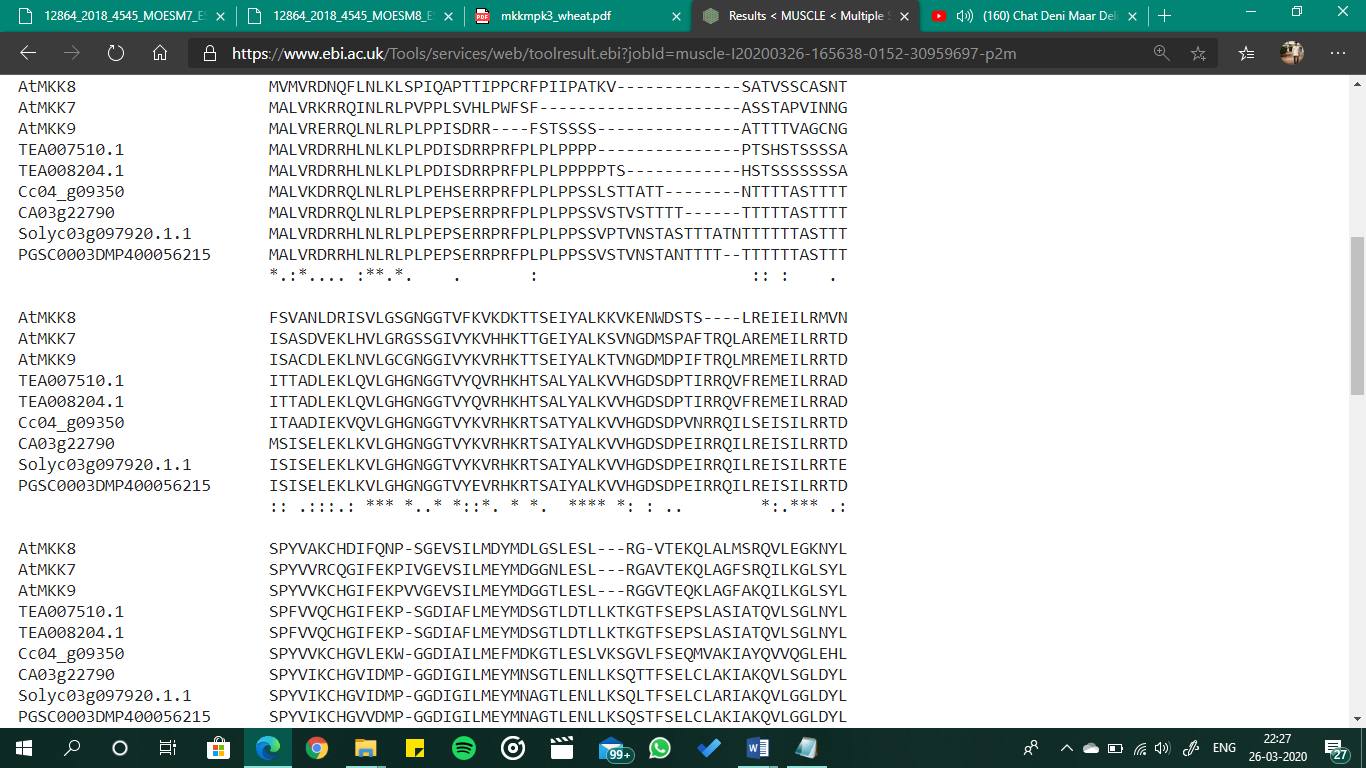

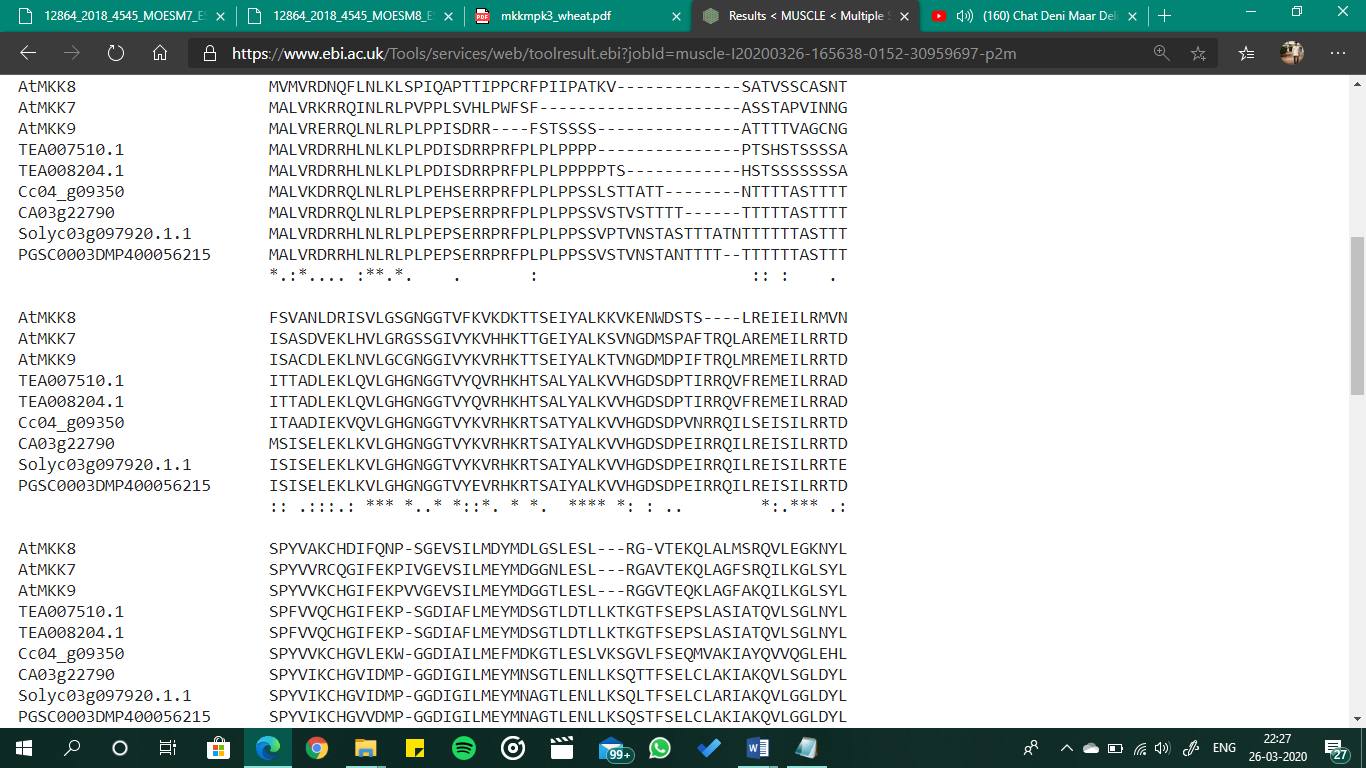

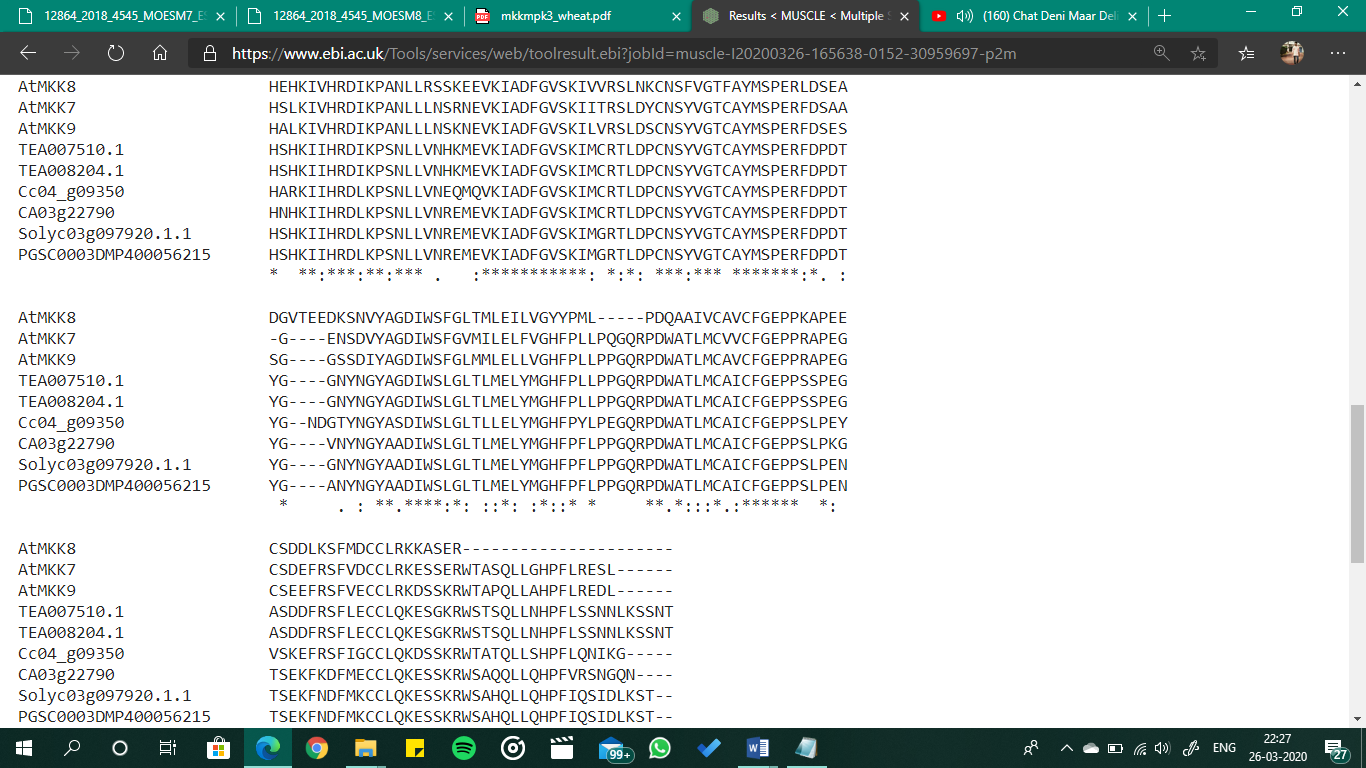

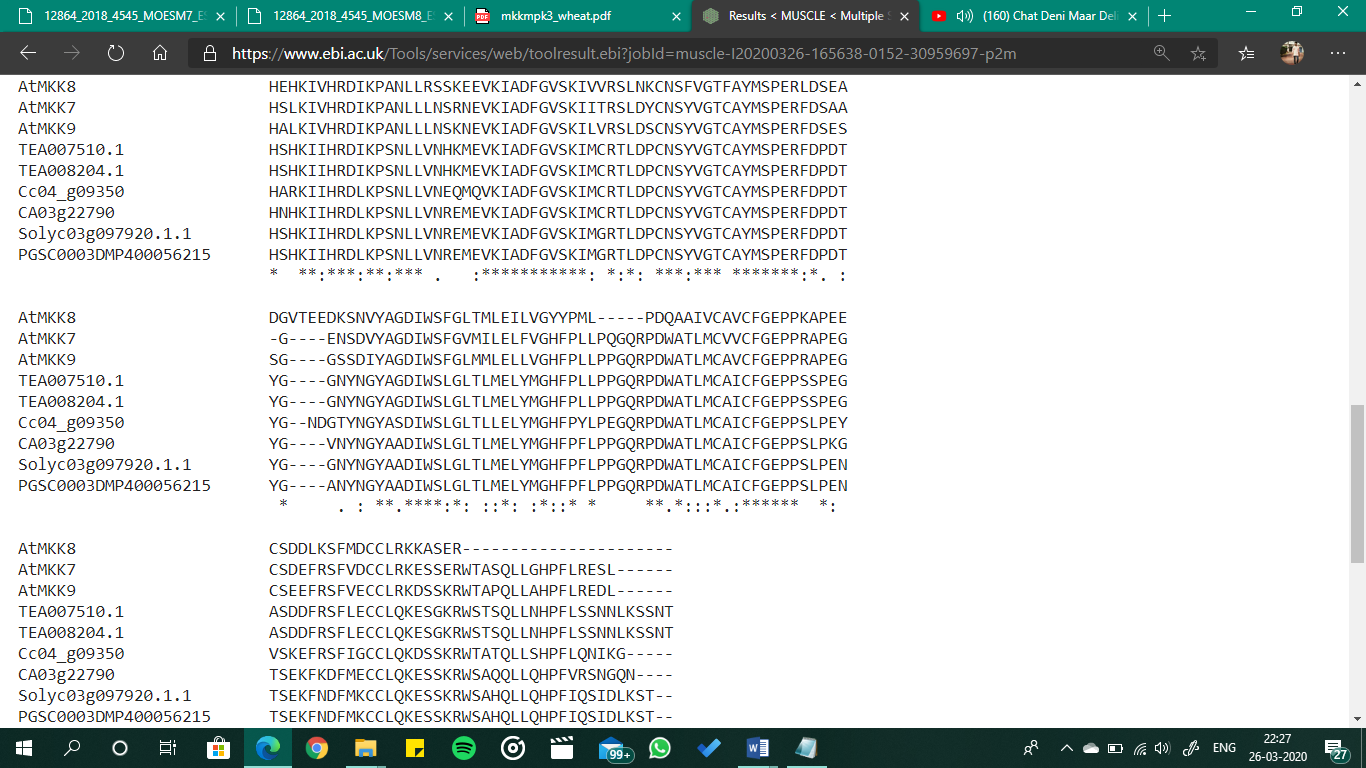

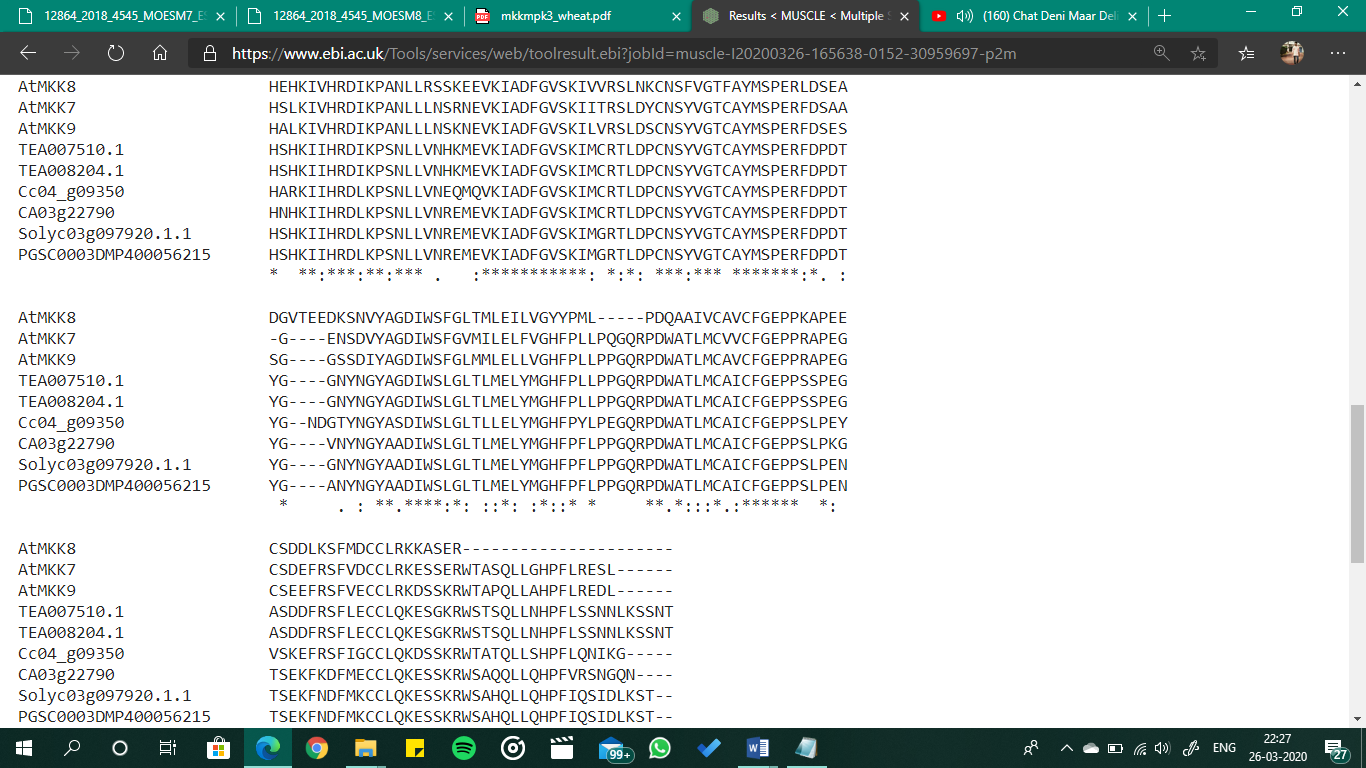


**GxGxxG**

**DΨK**

**Supplementary Fig. S8 Alignment of domains in MKKs.** All the MKK protein sequences were subjected to alignment by MUSCLE tool owing to their sequence diversities. Sequences that are highlighted are ATP binding signature, marked in blue that consists the P loop consensus sequence (GxGxxG), the catalytic C loop, marked in light red colour that consists the DΨK consensus, the activation T loop, marked in green colour and NTF2 domain marked in greyish colour. Clade C and D show sequence derivations from the S/TxxxxxS/T activation loop and are marked in a lighter shade of green colour.
